# Evaluating stably expressed genes in single cells

**DOI:** 10.1093/gigascience/giz106

**Published:** 2019-09-16

**Authors:** Yingxin Lin, Shila Ghazanfar, Dario Strbenac, Andy Wang, Ellis Patrick, David M Lin, Terence Speed, Jean Y H Yang, Pengyi Yang

**Affiliations:** 1 School of Mathematics and Statistics, University of Sydney, Sydney, NSW 2006, Australia; 2 Cancer Research UK Cambridge Institute, University of Cambridge, Li Ka Shing Centre, Robinson Way, Cambridge CB2 0RE, UK; 3 Sydney Medical School, University of Sydney, Sydney, NSW 2006, Australia; 4 Westmead Institute for Medical Research, University of Sydney, Westmead, NSW 2145, Australia; 5 Department of Biomedical Sciences, Cornell University, Ithaca, NY 14853, USA; 6 Bioinformatics Division, Walter and Eliza Hall Institute of Medical Research, 1G Royal Parade, Parkville, VIC 3052, Australia; 7 Department of Mathematics and Statistics, University of Melbourne, Melbourne, VIC 3010, Australia; 8 Computational Systems Biology Group, Children’s Medical Research Institute, University of Sydney, Westmead, NSW 2145, Australia

**Keywords:** stably expressed genes, single cells, scRNA-seq, housekeeping genes, gene expression variability

## Abstract

**Background:**

Single-cell RNA-seq (scRNA-seq) profiling has revealed remarkable variation in transcription, suggesting that expression of many genes at the single-cell level is intrinsically stochastic and noisy. Yet, on the cell population level, a subset of genes traditionally referred to as housekeeping genes (HKGs) are found to be stably expressed in different cell and tissue types. It is therefore critical to question whether stably expressed genes (SEGs) can be identified on the single-cell level, and if so, how can their expression stability be assessed? We have previously proposed a computational framework for ranking expression stability of genes in single cells for scRNA-seq data normalization and integration. In this study, we perform detailed evaluation and characterization of SEGs derived from this framework.

**Results:**

Here, we show that gene expression stability indices derived from the early human and mouse development scRNA-seq datasets and the "Mouse Atlas" dataset are reproducible and conserved across species. We demonstrate that SEGs identified from single cells based on their stability indices are considerably more stable than HKGs defined previously from cell populations across diverse biological systems. Our analyses indicate that SEGs are inherently more stable at the single-cell level and their characteristics reminiscent of HKGs, suggesting their potential role in sustaining essential functions in individual cells.

**Conclusions:**

SEGs identified in this study have immediate utility both for understanding variation and stability of single-cell transcriptomes and for practical applications such as scRNA-seq data normalization. Our framework for calculating gene stability index, "scSEGIndex," is incorporated into the scMerge Bioconductor R package (https://sydneybiox.github.io/scMerge/reference/scSEGIndex.html) and can be used for identifying genes with stable expression in scRNA-seq datasets.

## Background

A hallmark of single-cell RNA-sequencing (scRNA-seq) data has been the remarkable variation in gene transcription that occurs at the level of individual cells [1]. The high degree of variation has led to the appreciation that transcription of genes at the single-cell level is comparatively noisier than on the cell population level [2]. Indeed, a subset of genes are thought to be characterized by their stochastic expression [3]. Supporting this notion, genes were found to show transcriptional bursting, where their expression varies drastically in individual cells [4,5]. Furthermore, a large number of genes from scRNA-seq data exhibit bimodality or multimodality of non-zero expression values [6], suggesting that many of these genes may be expressed at different levels in the same and/or different cells. These phenomena illustrate that expression stochasticity is an intrinsic property of many genes on the single-cell level [7].

On the cell population level, however, a subset of genes traditionally referred to as housekeeping genes (HKGs) [8,9] are found to be stably expressed in different cell types, tissue types, and developmental stages [10]. The concept of HKGs is often related to the gene set required to maintain basic cellular functions and therefore is crucial to the understanding of the core transcriptome that is required to sustain life [11–13]. Early studies [8, 14–16] were conducted to define HKGs using serial analysis of gene expression (SAGE) or microarrays. With the advent of biotechnologies, follow-up studies using more comprehensive data sources [17, 18] and high-throughput RNA sequencing (RNA-seq) [10, 19] have refined the list of HKGs from populations of cells.

When the findings from bulk transcriptome data of cell populations and the stochasticity in gene expression observed in individual cells from scRNA-seq data are taken together, several fundamental questions arise including (i) can patterns of stably expressed genes be identified from single-cell data? And if so, (ii) how stable are they across individual cells from different tissue types and biological systems? (iii) What properties do such genes have? And (iv) how do they compare to HKGs defined from bulk transcriptome data? In the present study, we set out to answer each of these questions.

Leveraging the advances of scRNA-seq techniques [20,21], we have previously developed a computational framework to rank genes based on various properties extracted from scRNA-seq data to characterize their expression stability in individual cells [22]. These genes were subsequently used for scRNA-seq data normalization and integration. To address the questions posed above, here, we applied the proposed framework on 2 high-resolution scRNA-seq datasets in which a wide range of cell types and developmental stages were profiled in human [23] and mouse [24], and also the "Mouse Atlas" scRNA-seq dataset that comprehensively profiled across major mouse organs and tissue types [25]. We referred to the list of stably expressed genes derived from these datasets as “hSEG” and “mSEG” for human and mouse, respectively, and collectively as “SEGs." We subsequently evaluated the stability of SEGs on a collection of independent scRNA-seq datasets generated from diverse tissues and biological systems, and different sequencing protocols. Compared to HKGs previously defined using bulk microarray [15] or RNA-seq datasets [10], SEGs identified on the single-cell level are considerably more stable in all tested biological systems, demonstrating the higher resolution enabled by scRNA-seq data for identifying genes that are truly stably expressed across individual cells, and suggesting their potential roles in maintaining essential functions in individual cells.

Our analyses highlight the previously unappreciated gene stability at the single-cell level. Our computational framework, incorporated as part of the scMerge Bioconductor R package, also allows further identification and refining of SEGs in other scRNA-seq datasets. This will have broad applications in normalization [26,27] and removal of unwanted variation [22,28,29] in scRNA-seq as well as bulk sequencing datasets generated from various experiments.

## Data Description

### scRNA-seq data processing

A collection of 11 publicly available scRNA-seq datasets (Table 1) were used in this study. These datasets were downloaded from either NCBI GEO repository or the EMBL-EBI ArrayExpress repository. Fragments per kilobase of transcript per million (FPKM) values or counts per million (CPM) from their respective original publications were used to quantify full-length gene expression for datasets generated by SMARTer or SMART-Seq2 protocols. Unique molecular identifier–filtered counts were used to quantify gene expression for the InDrop dataset. Data were transformed by log_2_(*x* + 1), where *x* is the original quantification (e.g., CPM). All datasets have undergone cell-type identification using biological knowledge assisted by various clustering algorithms from their respective original publications, which we retain for evaluation purposes. For each dataset, genes with >80% missing values (zeros) were removed, with the remaining genes considered as expressed in that dataset. These filtered datasets were used for all subsequent analyses.

**Table 1:** Summary of scRNA-seq datasets used for stably expressed gene identification and/or evaluation in the present study

ID	Publication	Description	Organism	No. cell	No. class	Protocol	Purpose
E-MTAB-3929	[23]	Human development	Human	1,529	5	SMART-Seq2	Identify
GSE45719	[24]	Mouse development	Mouse	269	8	SMART-Seq2	Identify
GSE109774	[25]	Mouse atlas	Mouse	41,965	68	SMART-Seq2	Identify
GSE94820	[31]	Peripheral blood mononuclear cells	Human	1,140	5	SMART-Seq2	Evaluate
GSE75748	[32]	Pluripotent stem cells and endoderm progenitors	Human	1,018	7	SMARTer	Evaluate
GSE72056	[33]	Multicellular metastatic melanoma	Human	4,645	7	SMART-Seq2	Evaluate
GSE67835	[34]	Adult and fetal brain	Human	466	8	SMARTer	Evaluate
GSE60361	[35]	Cortex and hippocampus	Mouse	3,005	7	SMARTer	Evaluate
GSE52583	[36]	Developmental lung epithelial cells	Mouse	198	4	SMARTer	Evaluate
E-MTAB-4079	[37]	Mesoderm diversification	Mouse	1,205	4	SMART-Seq2	Evaluate
GSE84133	[38]	Pancreas inter- and intracells	Mouse	822	13	InDrop	Evaluate

## Analyses

### A computational framework for measuring gene expression stability in single cells

**Figure 1:**
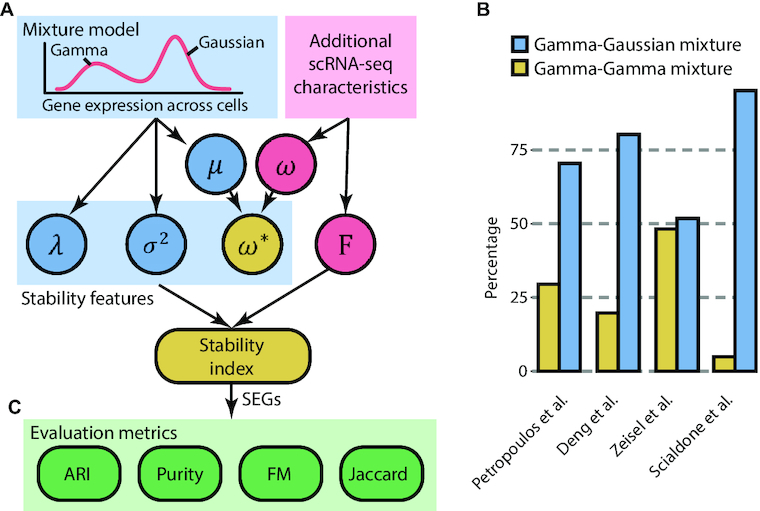
Schematic illustration of the computational framework for deriving gene stability index on the single-cell level. (A) Stability features extracted directly from the mixture model are colored in blue. Those extracted from additional scRNA-seq data characteristics are in red. The overall stability index is derived from the combination of all stability features. (B) Comparison of Gamma-Gaussian and Gamma-Gamma mixture models on 4 scRNA-seq datasets (i.e. E-MTAB-3929, GSE45719, GSE60361, E-MTAB-4079). *y*-axis represents the percentage of times a given model is selected by Bayesian information criterion. (C) Evaluation metrics used for evaluating gene expression stability in scRNA-seq datasets.

We recently proposed a mixture-modeling computational framework for characterizing gene expression stability using scRNA-seq data [22]. The original framework uses a Gamma component to fit the lower end of the distribution given the non-negative values of gene expression [30] and a Gaussian component to fit the higher end for capturing variability in cells that express a given gene (Fig. 1A). To test whether a Gamma component would be better suited for fitting the higher end of the distribution, here we compared the choices of Gamma-Gaussian and Gamma-Gamma mixture models using Bayesian information criterion. We found that the Gamma-Gaussian mixture fits consistently better than Gamma-Gamma mixture across multiple datasets (Fig. 1B).

Using the Gamma-Gaussian mixture model, we extract a set of stability features including λ, σ^2^, ω*, and the *F*-statistics, and derive a stability index for each gene on the single-cell level. The μ and σ^2^ denote the mean and variance of the Gaussian component fitted to a gene *x* across individual cells. The joint density function *f*(.) is defined as follows:

$$\begin{equation*}
f(.)=\lambda \frac{\beta ^{\alpha }}{\Gamma (\alpha )}x^{\alpha -1}e^{-\beta x}+(1-\lambda )\frac{1}{\sigma \sqrt{2\pi }}e^{-\frac{(x-\mu )^2}{2\sigma ^2}},
\end{equation*}
$$

where 0 ≤ λ ≤ 1 is the mixing proportion indicating the proportion of cells in the Gamma component in the fitted model. Genes whose expression profiles are with low mixing proportion (λ) and small variance (σ^2^) are unimodal and relatively invariant across cells and therefore more likely to be stably expressed.

The ω denotes the percentage of zeros of a gene across cells. The measured expression level for a given gene and cell may be zero due to technical dropout, stochastic expression, or no transcription occurring at all for that gene [39]. Thus, SEGs would have relatively small ω (i.e., low proportion of zeros) because they are expected to be expressed in all cells. However, genes with a low level of expression tend to have a higher proportion of zeros than highly expressed genes simply due to technical dropouts [40]. We therefore regularized the proportion of zeros (ω) of each gene based on its average expression level μ in the Gaussian component by ω* = ω · minmax(μ), where minmax(.) scales the ω* to the range of 0–1. This regularization accounts for the dropout bias towards genes with lower expression.

When predefined cell type annotation is available for a given dataset, the *F*-statistics can be used as another stability feature to select for genes in which we observe the same average gene expression across different predefined cell types. Together, genes with small λ, σ^2^, ω*, and *F*-statistic are unimodal, expressed with low variance, with relatively low percentage of zeros, and expressed similarly across all cell types, respectively, and are more likely to be stably expressed.

The expression stability index is defined for each gene by combining these 4 stability features. Specifically, genes are ranked first in increasing order with respect to λ, σ^2^, ω*, and *F*-statistics; and the ranks from each stability feature are rescaled to range from 0 to 1. The stability index for each gene is defined as the average of its scaled rankings across all 4 stability features. Thus, genes are ranked in terms of their degree of evidence towards expression stability in individual cells and can be selected by adjusting the stability index threshold. The subsequent evaluation can be conducted to assess the stability and generalization property of selected SEGs in other biological systems using various evaluation metrics (Fig. 1C).

### Genes are reproducibly ranked by their expression stability in single cells

To investigate whether some genes are inherently more stable in expression on the single-cell level, we used 3 high-resolution scRNA-seq datasets (e.g., human development, mouse development, and the mouse atlas) to quantify genes that are expressed at steady levels across different cell types, tissues, and developmental stages of human and mouse, respectively (Table 1; datasets labeled as "identify"). These datasets provide a starting point for identifying SEGs that can then be used for evaluation on various cell/tissue types and biological systems (Table 1; datasets labeled as "evaluate").

**Figure 2:**
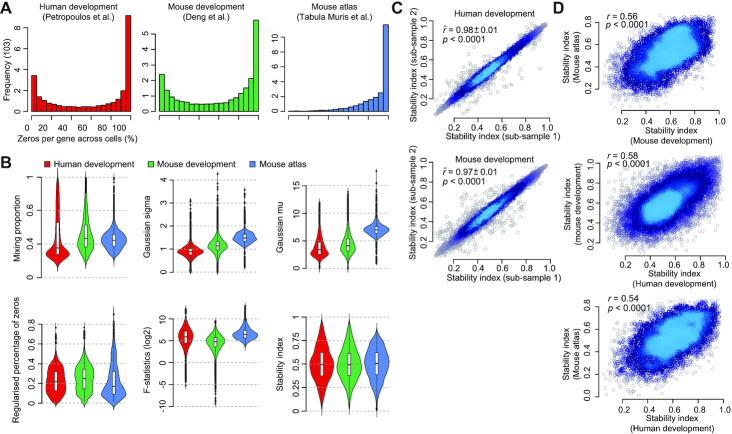
Characterizing gene stability features in single cells for human and mouse. (A) Percentage of zeros per gene across individual cells. (B) Fitted values of mixing proportion (λ), and variance (σ^2^) and mean (μ) in the Gaussian component (top panels) of the mixture model for each gene. Regularized percentage of zeros, *F*-statistics computed from predefined cell class (bottom left panel), and stability index derived for each gene (bottom right panel), respectively. (C) Scatter plot of stability index calculated from 2 random subsamplings of cells from human and mouse development datasets. Mean Pearson’s correlation coefficient and standard deviation ($\bar{r}\pm \mathrm{sd}$) were calculated from pairwise comparison of 10 repeated random subsamplings on each dataset. (D) Scatter plot and correlation of stability indices calculated from each of 3 datasets. *P*-values denote *t*-distribution test on Pearson’s correlation coefficient.

We first looked at the proportion of zeros per gene across all profiled cells in the early human and mouse development scRNA-seq datasets, respectively. We found that a large percentage of genes have >50% zero quantification across cells in both datasets (Fig. 2A), suggesting that most of the genes are transiently expressed in different cell/tissue types and during different developmental stages in both human and mouse. We observed that the distributions of stability features across the 3 scRNA-seq datasets are different (Fig. 2B). Nevertheless, our rank-based approach scales ranks of genes with respect to each stability feature and derives highly comparable stability index distributions from each dataset (Fig. 2B, bottom right panel).

We next investigated the reproducibility of the stability index by randomly sampling 80% of all cells from the human and mouse development datasets and recalculating the stability index for each subsample. We found the stability index to be highly reproducible (Fig. 2C) within a dataset with average Pearson correlation coefficients of 0.98 and 0.97. In comparison, the correlation of stability indices from the mouse development and mouse atlas datasets are much more moderate (Fig. 2D), suggesting room for further improvement when more comprehensive and deeper scRNA-seq datasets become available. We also observed that the stability indices derived for human and mouse are significantly correlated (Fig. 2D).

### Comparative analysis of SEGs identified in single cells and HKGs defined from bulk transcriptome

**Figure 3:**
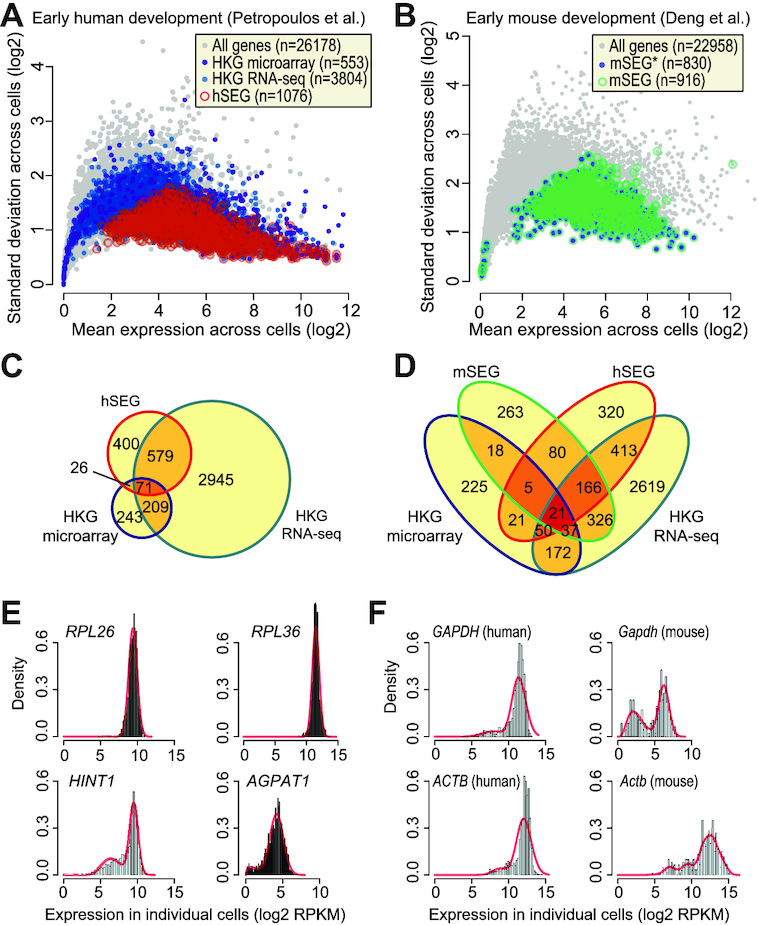
Comparison of SEGs identified on individual cell level using scRNA-seq with HKGs defined on cell population level using bulk transcriptome data. (A) Scatter plot showing mean expression (*x*-axis) and variance (*y*-axis) of each gene (gray circles) across profiled single cells. Open red circles represent SEGs identified from early human development data (hSEG) in this study whereas dark and light blue solid circles represent HKGs defined previously using bulk microarray [15] and RNA-seq data [10]. (B) Same as (A) but for SEGs identified from early mouse development data (mSEG*; light blue points) and the union of these identified from both mouse development and mouse atlas datasets (mSEG; green circles). (C) Venn diagrams showing overlaps of hSEGs and HKGs defined using bulk microarray and RNA-seq. (D) Overlap of all human and mouse gene lists. (E) Expression patterns of example genes that are defined as SEGs using scRNA-seq data but not as HKGs using bulk microarray or RNA-seq data (*RPL*26 and *RPL*36) and vice versa (*HINT* and *AGPAT*1) across individual cells. (F) Expression patterns for *GAPDH* and *ACTB* in human and mouse (*Gapdh* and *Actb*) across individual cells.

To understand the relationships of genes with stable expression in single cells with HKGs defined previously with bulk microarray [15] and RNA-seq [10], we derived a list of SEGs for human and mouse, respectively, by computing the rank percentiles of the stability index as well as the 4 stability features. Genes with a stability index rank percentile >80 as well as a reversed rank percentile >60 for each of the 4 stability features were included in the SEG list. For mouse, we took the union of the SEGs identified from the mouse development and mouse atlas datasets. This resulted in lists of 1,076 human (hSEG) and 916 mouse (mSEG) genes, respectively (Fig. 3A and B). In comparison to the HKGs defined previously using bulk transcriptomes, we found that hSEG identified on the single-cell level have significantly smaller expression variances across individual cells (Fig. 3A).

Comparing with previously defined HKGs (Fig. 3C), there were 676 common genes between our hSEG list and those defined by microarray or bulk RNA-seq. This accounts for 62% of hSEGs, a statistically significant overlap (permutation *P* < 2e−5), highlighting a high level of commonality but also uniqueness of SEGs. For the human and mouse SEG lists derived from scRNA-seq datasets, there were 272 common genes (Fig. 3D), which accounts for a significant portion of genes in both lists (25% with respect to hSEG and 30% with respect to mSEG; permutation *P* < 2e−5), in agreement with the correlation analysis (Fig. 2D), suggesting their conservation between human and mouse.

To investigate the difference between SEGs and HKGs defined by bulk transcriptomes, we inspected a few individual genes that were defined as SEGs using scRNA-seq data but not HKGs by bulk microarray or RNA-seq, and vice versa. We discovered that many ribosomal proteins (such as *RPL26* and *RPL36*) that were included in the SEG list but not in the HKG lists (Fig. 3E) showed strong unimodal expression patterns across all cells. In contrast, genes such as *HINT1* (histidine triad nucleotide-binding protein 1) and *AGPAT1* (1-acylglycerol-3-phosphate O-acyltransferase), both of which have been reported to be differentially expressed in brain tissue [41] or malignant esophageal tissues [42] compared to normal samples, were included in both microarray and RNA-seq–defined HKG lists, but not in the SEG list owing to their bimodal expression patterns across individual cells.

Finally, we examined the expression patterns of *GAPDH* and *ACTB* (Fig. 3F), genes that are commonly treated as canonical HKGs for data normalization, and observed clear bimodality in both the human and mouse data. In agreement with previous studies [10,17,26, 43], these data argue against their use as “housekeeping genes” for sample normalization.

### SEGs exhibit strong expression stability in single cells across different tissues and biological systems

**Figure 4:**
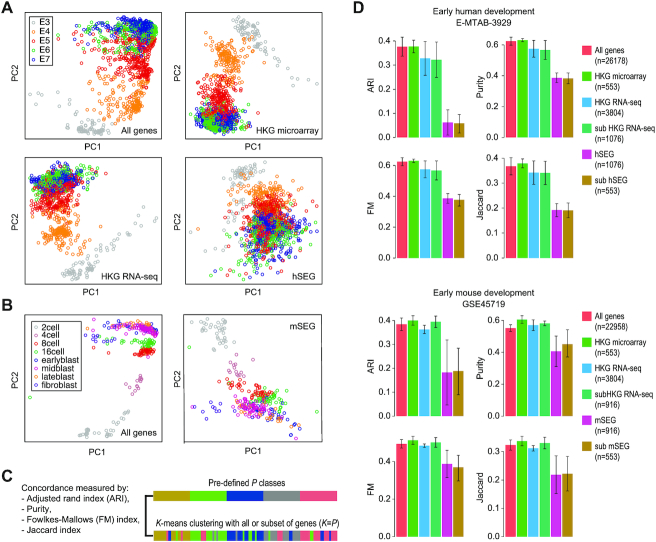
Stability of SEGs and HKGs in human and mouse development scRNA-seq datasets. (A) PCA plots generated from human development data using all expressed genes, HKGs, or hSEGs. Cells are colored by their predefined developmental stages. (B) PCA plots generated from mouse development data using all expressed genes or mSEGs. Cells are colored by their predefined types and developmental stages. (C) Schematic showing the quantification of concordance of *k*-means clustering with predefined cell classes using a panel of metrics. (D) Bar plots of concordance between *k*-means clustering and predefined cell class labels, using all expressed genes, HKGs identified from microarray and RNA-seq data, SEGs identified from this study for human (hSEGs) and mouse (mSEGs), and size-matched subset of HKGs to SEGs and vice versa.

We hypothesized that if the expression levels of the SEGs are relatively stable, they should show relatively small expression differences across the different cell types from various biological systems. We first investigated principal component analysis (PCA) plots generated from early human and mouse development data using all genes (all expressed messenger RNA [mRNA]), or subsets of genes defined for human (i.e., HKG microarray, HKG RNA-seq, and hSEG) (Fig. 4A) and mouse (i.e., mSEG) (Fig. 4B). We found that for human data there is clear separation of developmental stages in the first 2 principal components when PCA plots were created by using either all genes, or HKGs defined from microarray or RNA-seq, suggesting that genes that were expressed differentially in different developmental stages were driving the separation. In contrast, the PCA plot generated from using hSEG shows much less separation with respect to the developmental stages, suggesting that they are generally expressed at a similar level across individual cells irrespective of cell differentiation and change of developmental stages. Similar results were observed from mouse development data (Fig. 4B), where the PCA plot generated from mSEG shows less separation of cell type and development stage compared to the PCA plot generated from using all genes.

To quantify the above visual observations in human and mouse developmental datasets, we used *k*-means clustering to partition cells into 5 and 8 clusters, respectively, using all genes (all expressed mRNA) or subsets of genes defined in each list (i.e., hSEG, mSEG, HKG microarray, and HKG RNA-seq) with the hypothesis that clusters arising from using SEGs and HKGs will exhibit lower concordance with predefined cell type- and tissue-specific labels (Fig. 4C), thereby demonstrating consistent levels of expression across different cell and tissue types. To account for the size difference of the gene lists, we also created subsets of HKGs identified from RNA-seq data (sub HKG RNA-seq) to match the sizes of hSEGs and mSEGs, respectively; and subsets of hSEGs (sub hSEG) and mSEGs (sub mSEG) to match the size of HKGs identified from microarray data.

We found that *k*-means clustering outputs using SEGs derived from scRNA-seq data showed the lowest concordance to their predefined cell class labels (i.e., embryonic day of development or cell types) as quantified by the adjusted Rand index (ARI), Purity, Fowlkes-Mallows index (FM), and Jaccard index (Fig. 4D). The reduction of either list to match the other had relatively minor effect on the clustering results. These results demonstrate that SEGs are stably expressed across cells and developmental stages in the 2 scRNA-seq datasets.

To test whether the SEGs derived above are stably expressed in other cell and tissue types, we evaluated these SEGs and their subsets that matched the size of HKGs defined from microarray data on 8 datasets (Table 1), which are independent of the scRNA-seq datasets used for identifying SEGs. These additional datasets represent drastically different tissues and biological systems in both human and mouse, as well as different sequencing protocols and a wide range in the number of cells sequenced.

Similar to the above section, we quantified the clustering concordance with respect to each of their predefined cell class labels using each of the 4 concordance metrics (ARI, Purity, FM, and Jaccard) (Table 2). We found that on average, clustering using SEGs (and their subsets) gave the lowest concordance to the predefined cell type- and tissue-specific class labels in all tested datasets compared to clustering using all expressed genes or HKGs defined using bulk microarray and RNA-seq datasets. These results suggest that SEGs defined in early human and mouse development also display strong expression stability in various cell/tissue types and biological systems, and they are considerably more stable than HKGs defined using bulk transcriptome data on the single-cell level.

**Table 2:** Stability evaluation results on independent scRNA-seq datasets that profile various cell types and biological systems

	Peripheral blood mononuclear cells (human) [31]					Pluripotent stem cells and endoderm progenitors (human) [32]				
Index		HKG		SEG			HKG		SEG	
	All genes	Array	RNA-seq	n = 1,076	n = 553	All genes	Array	RNA-seq	n = 1,076	n = 553
ARI	55 ± 8	42 ± 3	38 ± 4	29 ± 6	21 ± 3	69 ± 5	58 ± 5	55 ± 6	41 ± 3	40 ± 3
Purity	69 ± 7	62 ± 2	59 ± 1	52 ± 5	48 ± 5	80 ± 4	74 ± 3	71 ± 5	59 ± 3	61 ± 4
FM	67 ± 5	56 ± 1	52 ± 3	45 ± 4	40 ± 2	75 ± 4	66 ± 4	63 ± 5	51 ± 2	50 ± 3
Jaccard	49 ± 6	39 ± 1	35 ± 2	29 ± 4	25 ± 2	60 ± 5	48 ± 4	46 ± 6	34 ± 2	33 ± 2
	Multicellular metastatic melanoma (human) [33]					Adult and fetal brain (human) [34]				
		HKG		SEG			HKG		SEG	
	All genes	Array	RNA-seq	n = 1,076	n = 553	All genes	Array	RNA-seq	n = 1,076	n = 553
ARI	31 ± 5	18 ± 2	18 ± 1	15 ± 1	15 ± 1	53 ± 7	50 ± 3	39 ± 4	36 ± 3	34 ± 3
Purity	80 ± 5	73 ± 1	74 ± 1	71 ± 1	70 ± 1	82 ± 3	76 ± 4	74 ± 3	68 ± 2	65 ± 1
FM	51 ± 3	39 ± 2	40 ± 1	37 ± 1	36 ± 1	62 ± 6	59 ± 2	50 ± 3	47 ± 3	46 ± 3
Jaccard	32 ± 2	22 ± 2	24 ± 1	21 ± 1	20 ± 0	44 ± 6	41 ± 2	33 ± 3	30 ± 3	29 ± 2
	Cortex and hippocampus (mouse) [35]					Developmental lung epithelial cells (mouse) [36]				
		HKG		SEG			HKG		SEG	
	All genes	Array	RNA-seq	n = 916	n = 553	All genes	Array	RNA-seq	n = 916	n = 553
ARI	45 ± 8	36 ± 5	31 ± 3	28 ± 3	26 ± 2	61 ± 6	55 ± 4	48 ± 2	46 ± 0	43 ± 5
Purity	72 ± 3	66 ± 1	63 ± 1	59 ± 1	58 ± 2	83 ± 4	80 ± 2	76 ± 1	75 ± 0	73 ± 3
FM	55 ± 6	49 ± 4	44 ± 3	42 ± 2	40 ± 2	72 ± 4	68 ± 3	62 ± 2	61 ± 0	59 ± 4
Jaccard	38 ± 6	32 ± 4	28 ± 2	26 ± 2	25 ± 2	56 ± 5	51 ± 3	45 ± 2	44 ± 0	42 ± 4
	Mesoderm diversification (mouse) [37]					Pancreas inter- and intracells (mouse) [38]				
		HKG		SEG			HKG		SEG	
	All genes	Array	RNA-seq	n = 916	n = 553	All genes	Array	RNA-seq	n = 916	n = 553
ARI	54 ± 2	43 ± 8	49 ± 3	31 ± 7	10 ± 7	37 ± 4	22 ± 3	23 ± 3	19 ± 2	17 ± 3
Purity	66 ± 1	62 ± 6	65 ± 1	59 ± 7	48 ± 7	89 ± 3	78 ± 3	76 ± 2	74 ± 2	71 ± 2
FM	68 ± 1	63 ± 8	67 ± 1	59 ± 7	53 ± 5	52 ± 4	38 ± 3	39 ± 3	35 ± 2	32 ± 3
Jaccard	52 ± 1	46 ± 7	50 ± 1	40 ± 8	32 ± 7	30 ± 3	20 ± 3	21 ± 3	17 ± 2	16 ± 2

All indices are within the range of [0, 1] and are multiplied by 100.

### Gene stability index derived from single cells correlates with gene sequence and structural characteristics

**Figure 5:**
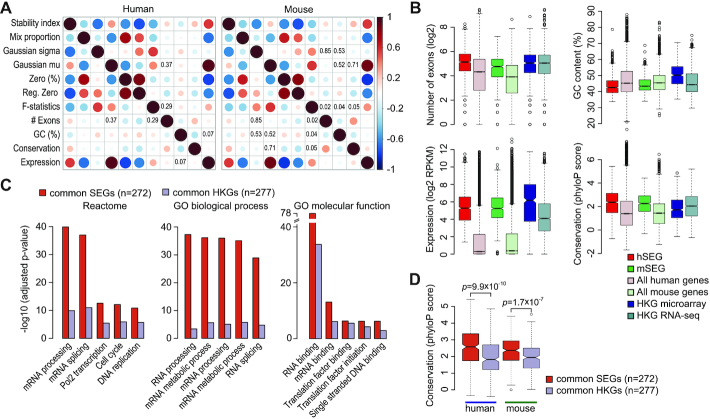
Characterization of stability index with sequence and gene characteristics. (A) Pearson correlation analyses of human and mouse gene stability features with respect to genomic structural and evolutionary gene features. *P*-values >0.001 are displayed. (B) Box plots of various gene characteristics for SEGs, HKGs, and all expressed genes. Coloured box captures lower quartile and upper quartile with median displayed as horizontal line in the middle. Dotted lines and bars represent whiskers. (C) Overrepresentation analyses of SEGs that are common between hSEG and mSEG (common SEGs); and HKGs that are common between HKG microarray and HKG RNA-seq (common HKGs), using Gene Ontology (GO) and Reactome databases. (D) Comparison of conservation for common SEGs and common HKGs in human and mouse genomes. *P*-values were calculated from a 2-sided Wilcoxon rank sum test.

To further characterize gene expression stability in single cells, we correlated the stability index and each stability feature extracted from scRNA-seq data with various gene structural and conservation features calculated from various data sources. We found that the stability index correlated positively with the number of exons in a gene, gene expression, and gene conservation, and negatively with GC content in the gene body in both human and mouse (Fig. 5A), many of which are characteristics of HKGs reported in previous studies. Consistent with this, we found that SEGs are more evolutionarily conserved [44] with higher phyloP scores. SEGs also possess more exons, in agreement with previous finding on HKGs [45], despite mouse genes on average having fewer exons than human genes. Both human and mouse SEGs appeared to have a slightly lower GC content, but, similar to previous observation on HKGs, the relation was relatively weak [46] (Fig. 5B).

Perhaps unsurprisingly, SEGs identified in this study possess similar characteristics to those observed in HKGs, indicating that they are serving essential cellular functions akin to HKGs. Supporting this, we found that multiple top-enriched Gene Ontology and Reactome terms that describe essential cellular functions are shared by common SEGs (genes overlap between hSEG and mSEG) and common HKGs (genes overlap between HKG microarray and HKG RNA-seq) (Fig. 5C) (see Methods for details). Nevertheless, common SEGs are far more enriched for most GO and Reactome terms than common HKGs defined from bulk transcriptome and also show significantly higher conservation in both human and mouse (Fig. 5D). These results indicate the higher resolution enabled by scRNA-seq data for identifying genes that are truly stably expressed across individual cells.

## Discussion

Since the emergence of high-throughput transcriptome profiling, the search for stably expressed genes (SEGs) has been a central quest in modern biology. Such genes are often thought to be essential for basic cellular functions given their relatively constant expression and activity despite changes in cell status and types. The hypothesis that such genes may serve the same housekeeping functions across various cell and tissue types has also led to their definition as “housekeeping genes” (HKGs). While the existence of true HKGs whose expression is universally constant across all cells and systems is a subject of debate [42, 47], their practical use as control genes for experimental data normalization is well appreciated.

Recent advances in single-cell transcriptome profiling using scRNA-seq have highlighted the phenomenal amount of gene expression stochasticity and heterogeneity in single cells. Compared to bulk transcriptome data that aggregate millions of cells to obtain a single gene expression measure, scRNA-seq data allows the expression dynamics of each gene within individual cells to be monitored, and therefore enables the identification of genes that are truly expressed at a steady level in individual cells across tissues and developmental stages. By modeling from large-scale scRNA-seq datasets, we quantified the relative expression stability of genes on the single-cell level. We showed that the SEGs derived based on their stability indices are considerably more stable in not only the scRNA-seq datasets from which they are identified but also independent scRNA-seq datasets that profile various cell types and biological systems.

Our analysis demonstrated that despite the high variability in single-cell gene expression, a subset of genes is inherently more stable in expression than other genes within individual cells. Their sequence and gene structural properties are strongly reminiscent of HKGs defined from bulk transcriptome, suggesting their essential roles in maintaining basic cellular functions on the individual cell level.

While a heuristic cut-off was used to select a set of SEGs, we note that the main purpose is primarily for evaluation and comparison with HKGs. The proposed framework provides a continuous stability index for each gene and therefore allows the selection of a desired number of genes based on the stability index according to specific applications. Moreover, the proposed framework can be applied in a data-dependent manner to rank genes based on their expression stability in a given scRNA-seq dataset. This relaxes the rigid binary definition of HKGs and enables a more practical definition of stable expression in different experimental contexts. Hence, the proposed method is particularly useful for defining stable or “control” genes in various scRNA-seq experiments, which is often a key step in normalizing such data [48,49]. Indeed, the utility of SEGs for scRNA-seq data normalization has already been demonstrated by our recent study on integrating multiple scRNA-seq datasets [22]. Nevertheless, the choice of dataset for deriving SEGs is important. Datasets used for SEG identification should contain normal cell types and profile heterogeneous tissues and cell types because data only containing homogeneous cell types cannot provide the foundation for identifying genes stably expressed in different tissues and cell types, and data containing abnormal cell types such as cancers may derive genes that are abnormally stable in cancers.

As mentioned above, generalizability of SEGs is dependent on the diversity of cell types profiled in a scRNA-seq experiment. Various cell atlas profiling initiatives such as the Human Cell Atlas [50] are currently under way to comprehensively characterize the transcriptome of every human cell. Information from such resources in conjunction with our computational framework will provide an even more precise assessment of gene expression stability in single cells that will enrich subsequent avenues of research including characterizing heterogeneity and stability of single-cell transcriptomes and their use for technical data normalization and standardization.

Taken together, this comprehensive evaluation study demonstrates the utility of measuring gene expression stability at the single-cell level and marks a shift in paradigm for selecting genes that are stably expressed in single cells for practical applications.

## Methods

### Evaluating the stability of gene lists

To assess the expression stability of each gene list in various cell types and biological systems, the *k*-means algorithm was used to cluster each scRNA-seq dataset to its predefined number of clusters and an array of evaluation metrics were applied to compute the concordance with respect to the predefined (“gold standard”) class labels. Evaluation metrics include the ARI, Purity, FM, and the Jaccard index.

Let $U=\lbrace u_1,u_2, ...,u_P\rbrace$ denote the true partition across *P* classes and $V=\lbrace v_1,v_2, ...,v_K\rbrace$ denote the partition produced from *k*-means clustering (*K* = *P*). Let *a* be the number of pairs of cells correctly partitioned into the same class by the clustering method; *b* be the number of pairs of cells partitioned into the same cluster but in fact belonging to different classes; *c* be the number of pairs of cells partitioned into different clusters but belonging to the same class; and *d* be the number of pairs of cells correctly partitioned into different clusters. Then the ARI [51], the Jaccard index [52], and the FM [53] can be defined as

$$\begin{eqnarray*}
\mathrm{ARI}&=&\frac{2(ad-bc)}{(a+b)(b+d)+(a+c)(c+d)};\\
\mathrm{Jaccard}&=&a/(a+b+c);\\
\mathrm{FM}&=&\sqrt{\left[a/(a+b)\right] \left[a/(a+c)\right]};
\end{eqnarray*}
$$

and the Purity [54] can be calculated as

$$\begin{equation*}
\mathrm{Purity}=\frac{1}{N} \sum \nolimits _i \max \nolimits _j |u_i \cap v_i|,
\end{equation*}
$$

where *N* is the total number of cells and *i* and *j* are the indices of clusters from clustering output *u_i_* and predefined class label *v_j_*.

For each dataset, we calculated and compared the above 4 metrics using (i) all expressed genes, (ii) HKGs defined using microarray data [15], (iii) HKGs defined using bulk RNA-seq data [10], and (iv) SEGs identified in this study. To account for potential effects of gene list length, we also generated random subsets with the same number of genes in our SEG lists first by randomly sampling from all expressed genes in the dataset, and second by randomly sampling from the HKG list defined by bulk RNA-seq. Because the *k*-means clustering algorithm is not deterministic and the random sampling process introduces variability, the above procedure was repeated 10 times to account for such variability.

### Gene properties

To characterize SEGs identified in early human and mouse development datasets, we extracted gene sequence and structural features including the number of exons and percentage GC content in the gene body for human and mouse, respectively, using biomaRt [55]. Additionally, to characterize gene evolutionary conservation, phyloP scores were downloaded from the UCSC Genome Browser for mouse (mm10) and human (hg38) genomes. Exonic bases of each gene were determined based on GENCODE Genes for human (release 26) and mouse (release 14). The set of conservation scores for each gene was averaged for each gene. We assessed the concordance of gene expression stability index and each stability feature derived from single cells with structural features, conservation scores, and their expression across all genes for human and mouse using Pearson correlation coefficients. We also compared these features for SEGs and previously defined HKGs against all expressed genes in human and mouse, respectively.

### Gene ontology enrichment analysis

To perform GO enrichment analysis, we first defined SEGs that are shared between hSEG and mSEG as “common SEGs” and HKGs that are shared between HKG microarray and HKG RNA-seq as “common HKGs." The similar numbers of common SEGs (256) and common HKGs (277) allowed us to avoid any potential gene set size bias in the enrichment analysis.

Overrepresentation of common SEGs or common HKGs was evaluated by comparing each set of genes against ontologies defined in the GO database [56] and those defined in the Reactome database [57]. Fisher’s exact test was used to assess statistical significance. Top-enriched ontologies from either common SEGs or common HKGs were combined for interpretation.

## Availability of supporting data and materials

The computational framework for calculating the gene stability index, "scSEGIndex," is deposited in the *GigaScience* GigaDB database [58].

## Availability of supporting source code and requirements

Project name: Single cell stably expressed genes

Project home page: https://sydneybiox.github.io/scMerge

Operating system(s): Platform independent

Programming language: R

License: GPL-3

## Additional files

**Supplementary information**: Supplementary Methods and Results are available via the additional file associated with this article.

**Supplementary Table S1. Lists of stably expression genes**.

giz106_GIGA-D-18-00467_Original_Submission

giz106_GIGA-D-18-00467_Revision_1

giz106_GIGA-D-18-00467_Revision_2

giz106_GIGA-D-18-00467_Revision_3

giz106_Response_to_Reviewer_Comments_Original_Submission

giz106_Response_to_Reviewer_Comments_Revision_1

giz106_Response_to_Reviewer_Comments_Revision_2

giz106_Reviewer_1_Report_Original_Submission Yong Hou -- 12/10/2018 Reviewed

giz106_Reviewer_1_Report_Revision_1 Yong Hou -- 6/10/2019 Reviewed

giz106_Reviewer_1_Report_Revision_2 Yong Hou -- 7/25/2019 Reviewed

giz106_Reviewer_2_Report_Original_Submission George Tseng -- 4/6/2019 Reviewed

giz106_Reviewer_2_Report_Revision_1 George Tseng -- 6/13/2019 Reviewed

giz106_Supplemental_Table

## Abbreviations

ARI: adjusted Rand index; EMBL-EBI: European Molecular Biology Laboratory European Bioinformatics Institute; FM: Fowlkes-Mallows index; GEO: Gene Expression Omnibus; GO: Gene Ontology; HKG: housekeeping gene; HKG microarray: housekeeping genes defined using bulk microarray; HKG RNA-seq: housekeeping genes defined using bulk RNA-seq; hSEG: stably expressed genes derived from early human developmental dataset; mRNA: messenger RNA; mSEG: stably expressed genes derived from early human developmental dataset; NCBI: National Center for Biotechnology Information; PCA: principal component analysis; SAGE: serial analysis of gene expression; scRNA-seq: single-cell RNA-sequencing; SEG: stably expressed gene; UCSC: University of California Santa Cruz.

## Competing interests

The authors declare that they have no competing interests.

## Funding

This work is supported by Australian Research Council (ARC)/Discovery Early Career Researcher Award (DE170100759) to P.Y., National Health and Medical Research Council (NHMRC)/Career Development Fellowship (1105271) to J.Y.H.Y., ARC/Discovery Project (DP170100654) grant to P.Y. and J.Y.H.Y., NHMRC/Program Grant (1054618) to T.P.S., and National Institutes of Health (NIH) R21 grant (DC015107) to D.M.L.

## Authors' Contributions

P.Y. conceived the study with input from J.Y.H.Y. All authors contributed to the design, analytics, interpretation, and the direction of the study. Y.L. and P.Y. led the analytics and A.W. led the curation of the datasets. All authors wrote, reviewed, edited, and approved the final version of the manuscript.
