# Supplementary material for: Evaluating stably expressed genes in single cells
Source: Gigascience. 2019 Sep 16;8(9):giz106. doi: 10.1093/gigascience/giz106 (PMC6748759; doi:10.1093/gigascience/giz106)
Supplement: giz106_GIGA-D-18-00467_Revision_1 [file giz106_giga-d-18-00467_revision_1.pdf]

GigaScience

Evaluating stably expressed genes in single cells

--Manuscript Draft--

**Manuscript Number:**	GIGA-D-18-00467R1
**Full Title:**	Evaluating stably expressed genes in single cells
**Article Type:**	Research
**Funding Information:**	Australian Research Council (DE170100759) — Dr Pengyi Yang<br>National Health and Medical Research Council (1105271) — Prof. Jean YH Yang<br>Australian Research Council (DP170100654) — Prof. Jean YH Yang<br>National Health and Medical Research Council (1054618) — Prof. Terence Speed
**Abstract:**	Background: Single-cell RNA-seq (scRNA-seq) profiling has revealed remarkable variation in transcription, suggesting that expression of many genes at the single-cell level are intrinsically stochastic and noisy. Yet, on cell population level, a subset of genes traditionally referred to as housekeeping genes (HKGs) are found to be stably expressed in different cell and tissue types. It is therefore critical to question whether stably expressed genes (SEGs) can be identified on the single-cell level, and if so, how their expression stability can be assessed? We have previously proposed a computational framework for ranking expression stability of genes in single cells for scRNA-seq data normalization and integration. In this study, we perform detailed evaluation and characterization of SEGs derived from this framework.<br><br>Results: Here, we show that gene expression stability indices derived from the early human and mouse development scRNA-seq datasets and the 'Mouse Atlas' dataset are reproducible and conserved across species. We demonstrate that SEGs identified from single cells based on their stability indices are considerably more stable than HKGs defined previously from cell populations across diverse biological systems. Our analyses indicate that SEGs are inherently more stable at the single-cell level and their characteristics reminiscent of HKGs, suggesting their potential role in sustaining essential functions in individual cells.<br><br>Conclusions: SEGs identified in this study have immediate utility both for understanding variation and stability of single-cell transcriptomes and for practical applications such as scRNA-seq data normalization. Our framework for calculating gene stability index, 'scSEGIndex', is incoporated into the scMerge Bioconductor R package (https://rdrr.io/bioc/scMerge/man/scSEGIndex.html) and can be used for identifying genes with stable expression in scRNA-seq datasets.
**Corresponding Author:**	Pengyi Yang<br>University of Sydney<br>Sydney, NSW AUSTRALIA
**Corresponding Author Secondary Information:**	
**Corresponding Author's Institution:**	University of Sydney
**Corresponding Author's Secondary Institution:**	
**First Author:**	Yingxin Lin
**First Author Secondary Information:**	
**Order of Authors:**	Yingxin Lin<br>Shila Ghazanfar

Powered by Editorial Manager® and ProduXion Manager® from Aries Systems Corporation

	Dario Strbenac
	Andy Wang
	Ellis Patrick
	Dave M Lin
	Terence Speed
	Jean YH Yang
	Pengyi Yang
**Order of Authors Secondary Information:**	
**Response to Reviewers:**	Reviewer Comments:

Reviewer #1:
In this manuscript, Lin and colleagues proposed a computational method to identify the stability expressed genes in single cell RNA-seq dataset. The authors downloaded two scRNA-seq dataset from the early human and mouse development, and performed their developed method, considered the mixture model Gamma-Gaussian distribution of the gene expression, to generated two SEG datasets. Then the authors did comparative analyses between the SEGs and HKGs defined previously from bulk samples, to demonstrate that SEGs are considerably more stable than HKGs. At last, the author extended the SEGs list to other eight additional datasets, and then detected some characteristics or features in SEG and comprise with those in HKGs.

In single cell transcriptome analysis, normalization and calibration are important, but difficult due to the gene expression stochasticity in single cell level. House keeping gene, rely on its stably expressed across tissues and species, always be used for normalization and calibration to reduce batch bias and background noises. SEGs proposed by the authors could be treated as a replacement of HKG for this purpose in single cell analysis. To do so, it's very important to generate a list of consistent and wildly stability expressed across different cell types, tissue, and even species. But obviously, this research serious lack this scope due to its limited samples and cell number. Moreover, for the comparative analysis between SEG and HKG on the human early development dataset, to me, the conclusion is not clarified. Because it seems that the authors selected TOP 1076 genes in SEG list, but TOP 3804 genes in HKG list previously generated in the citation (10). The huge difference of gene number maybe the most key factor in any statistical tests, so that any significant results for SEG could be overestimated.

Response: We thank this review for the insightful comments on our work. We note that, compared to the previously defined HKG lists, the human and mouse SEG lists generated from the human and mouse embryogenesis scRNA-seq datasets have been shown to be more effective for normalising and integrating multiple scRNA-seq datasets in our recently published work of scMerge (Lin et al. PNAS, 116:9775-9784, (2019) https://doi.org/10.1073/pnas.1820006116). We appreciate the criticism on the limitation of cell number in the two scRNA-seq datasets used to identify SEGs and we have now also utilised the 'mouse atlas' scRNA-seq data (SMART-Seq2) generated by the Tabula Muris Consortium (Nature 562, 367-372, 2018) for identifying SEGs in mouse.

We would like to emphasise that the purpose of this study is to evaluate the stability of genes on single-cell level as well as the proposed framework in identifying SEGs from scRNA-seq datasets. We do not suggest the current derived lists are final (albeit we have found them to be effective for normalising scRNA-seq datasets). The proposed framework (now implemented as part of the scMerge Bioconductor package (https://bioconductor.org/packages/release/bioc/html/scMerge.html)) can be applied to identify more refined SEGs from more comprehensive 'atlas' scRNA-seq datasets when they become available. This will allow increasingly high quality SEG lists to be defined/updated.

Major comments:

Powered by Editorial Manager® and ProduXion Manager® from Aries Systems Corporation

1) In comparative analysis between SEG and HKG, either increase the number of TOP SEG, or decrease HKG, to a comparable gene number in both sides. The 3804 HKGs, as described in citation (10), were generated by the criteria of reverse increasing order variance over tissues. In addition, it's important to do a future comparison between the TOP SEGs and the proposed 11 HKGs for calibration in citation (10).

Response: As suggested, we have decreased the HKG list defined using bulk RNA-seq to match our SEG lists and have also decreased the SEG lists to match the HKG list defined using bulk microarray. We found that the reduction of either list to match the other had relatively small effect on the clustering results as quantified by the four evaluation metrics. These new results are presented in Figure 4D and we have also updated all benchmark results in Table 2 using either full SEG lists or size matched lists in the revised manuscript.

We have previously tested the 11 HKGs defined in citation (10) for scRNA-seq data normalisation and integration using scMerge. The boxplots on the right side (attached in the "response letter with graphical panels") compare the effects of different gene lists for Liver data normalisation and integration (see Figure 1 and Figure 2 of Lin et al. PNAS, 116:9775-9784, (2019)). The y axis represents the F1 score of Silhouette coefficients between cell type mixing and 1-batch mixing, where higher values are more desirable. The results from using 11 HKGs are lower than using the full lists of HKGs or SEGs.

2) Expending the single cell RNAseq data as much as possible, such as tumor microenvirenment atlas, organ atlas, blood atlas, and brain atlas that published in recent years.

Response: As suggested, we have now included the 'mouse atlas' scRNA-seq dataset generated by the Tabula Muris Consortium (Nature 562, 367-372, 2018) to identify SEGs and updated the SEG list for mouse in the revised manuscript as well as all associated analyses and figure presentations. Having done this, we wish to note that this study is not intended to derive final SEG lists but to provide and evaluate the proposed analytic methods for identifying SEGs. We envisage to update the SEG lists at regular intervals as more comprehensive datasets (e.g. 'human cell atlas') become available.

3) To clarify the conclusion "The stability indices also showed relatively high correlation between human and mouse (Figure 2D), suggesting gene expression stability is conserved across species". I don't think that R=0.58 could be prove that. The overlap of hSEG and mSEG makes up about 24% and 31% in human and mouse respectively. Lacking any statistical test and directly evidences to demonstrate this conclusion.

Response: We appreciate this concern. We have now performed a permutation test by randomly selecting from all quantified human genes and overlapping them with mouse SEGs and vice versa. The distribution of the permutation (b = 50,000) are shown below (attached in the "response letter with graphical panels"). The average percentage of overlaps are around 2%. These results suggest that overlaps of 25% and 30% (based on the updated mouse SEGs) in human and mouse, respectively, are statistically significant. We have revised the manuscript to clarify this point (section "Comparative analysis of SEGs identified in…"; paragraph 2).

Minor comments:
4) Key words, "single cell; scRNA-seq; Single-cell transcriptome; Bulk transcriptome", are similar concepts regarding in the scope of this manuscript.

Response: We have updated the key words, removing “Single-cell transcriptome” and adding “Gene expression variability”.

5) List all of the SEGs in SM according stable indices.

Response: As suggested, we have now listed all SEGs according to their stable indices in supplementary table 1.

Powered by Editorial Manager® and ProduXion Manager® from Aries Systems Corporation

6) Page 4, Figure 3D. Suggest to choose gene from 11 gens listed in table 1 at citation (10) as the canonical HKG, instead of GAPDH and ACTB.

Response: We thank the reviewer for this suggestion. The purpose of Figure 3D (now Figure 3E of the revised manuscript) is to demonstrate the bimodality of some HKGs. We, of course, do not suggest all HKGs are bimodal and the choice here is for demonstrating the additional layer of information scRNA-seq data could provide.

7) Page 4, Figure 3A. It's strange that the value of log2(gene expression) could be smaller than 1.

Response: The gene expression values x (e.g. FPKM or CPM) are log-transformed as log2(x+1). Therefore, the transformed values may be smaller than 1. We have clarified this in "scRNA-seq data processing" section on page 2 of the revised manuscript.

8) Page 5, line 50-51, where is the section 3.3?

Response: We apologise for this typo. We have corrected this in the revised manuscript.

9) Page 5, line 43-45 in right half: do the calculating the payloP score on a equivalent gene number for SEG and HKG. Moreover, as Figure 5B showed the conservation of hSEG and HKG RNA-seq didn't result in that much difference.

Response: As suggested, we have now compared the conservation score (phyloP) using similar number of genes. Specifically, we used the overlap human and mouse SEGs (n=272) and the overlap HKGs from RNA-seq and microarray data (n=277). We then compared the conservation scores of these two lists of genes calculated from human and mouse genomes. We found that SEGs identified in this study are significantly more conserved compared to HKGs defined previously. We have now included these new results in Figure 5D of the revised manuscript.

10) Present more detailed results of functional analyses in the section of "Gene stability index derived from single cells correlates with gene sequence and structural characteristics", such as the results of GO enrichment, and other pathway analyses.

Response: As suggested, we have now included more enrichment analyses using GO (biological process and molecular function) and Reactome pathway database. These results are now presented in Figure 5C of the revised manuscript.

Reviewer #2:
This paper aims to identify stably expressed genes (SEGs) from single cell RNA-seq data. Conceptually, these genes are more stable genes than house-keeping genes (HKGs) detected by microarray or bulk RNA-seq data and there are many scientific merits in this paper. But there are also concerns described below.

Response:
We thank the constructive comments from this reviewer. We have addressed each of the point in detail as below.

Major:
1. Motivation and impact: Identifying house-keeping genes has its own significance scientifically but its impact is not emphasized enough to me in this paper. It is stated that the finding can improve normalization. In microarray era, house-keeping genes were indeed useful for normalizing intensity signals. But this utility is diminished in RNA-seq and scRNA-seq. Current normalization methods of scRNA-seq data (e.g. Bacher et al. and Lin et al. cited in this paper) do not utilize house-keeping genes. The authors should spend effort to explain further the general scientific impact of identified house-keeping genes.

Response: We thank the reviewer for this suggestion. We note that our work on scMerge (Lin et al. PNAS, 116:9775-9784, (2019),

https://doi.org/10.1073/pnas.1820006116) do utilises SEGs for scRNA-seq data normalisation. As suggested, we have now revised the manuscript to further highlight the utility of the SEGs identified from the proposed framework in scRNA-seq data normalisation and integration (Introduction, paragraph 4 and 5; and Discussion, paragraph 4).

2. Approach: The proposed algorithm raises concerns. (a) the selection of gamma-Gaussian model is not justified. Do real data support the model-fitting in most genes? Why the gamma tail is only on the left-end, not on the right-end? (b) Using 80 rank percentile and and minimal 60% in all criteria is an ad hoc selection. In general, there can be many ways to obtain SEGs. Is the current method better than, for example, previous methods for obtaining HKGs in bulk-RNA experiments? (c) Claiming a fixed set of house-keeping genes (a dichotomized decision) can be problematic. Providing a rank order with certain stability score is more helpful; for example, a biologist may be interested in obtaining the top 10 house-keeping genes for validation or internal control in experiment. (d) The description of w*=w\dcot minmax(\mu) is not clear.

Response: We appreciate these concerns. (a) The choice of the Gamma-Gaussian mixture model is explained in Ghazanfar et al. 2016 (https://doi.org/10.1186/s12918-016-0370-4). The reason for using a Gamma component for the left-end of the distribution is based on the non-negative values of gene expressions (log2(x+1)). To further investigate if a Gamma component will be more appropriate for the right-end of the distribution, we have now performed additional experiments comparing (1) Gamma-Gaussian mixture (in blue) and (2) Gamma-Gamma mixture (in yellow) models across four datasets using Bayesian information criterion (BIC) to determine optimal model choice. We found that in most cases Gamma-Gaussian mixture is selected according to BIC, suggesting it provides a better fit compared to Gamma-Gamma mixture (attached in the "response letter with graphical panels"). We have now incorporated these results in Figure 1B of the revised manuscript.

(b, c) We agree that using an "ad hoc" cutoff is not ideal. Nevertheless, we note that the selection of a set of SEGs is primarily for evaluation purposes and our framework does generate stability index for each gene and therefore provides a continuum ranking of all genes. We have included the stability index as well as other stability statistics of all genes in Supplementary Table 1. Biologists can select a desired number of genes based on the stability index for validation or internal control of their experiments. (d) We have now added more explanation to clarify the regularization of proportion of zeros (w*) in the revised manuscript (page 3, paragraph 3).

3. Evaluation: (1) The evaluation in Figure 3A and Figure 4 has over-fitting. The SEGs are identified from the scRNA-seq data and evaluated in the same dataset, which generated great improvement of the new SEGs compared to the old HKGs. On the other hand, the magnitude of improvement in Table 2 is significantly lower because the evaluation is performed in independent datasets. The authors should acknowledge this artifact and avoid exaggeration in the description. (2) Since there're four datasets in both human and mouse, an ultimate solution is to obtain SEGs from random subsets of data sets (subsets of one, two or three data sets) and perform evaluation of comparing SEGs and HKGs in the remaining independent datasets. The purpose is to evaluate whether combining increasing number of datasets can generate better SEGs. And if the conclusion is true, the final claimed SEGs should be obtained from combining all four datasets, in human and mouse separately.

Response: (1) We are aware of the overfitting in testing the stability of SEGs defined from the human and mouse development datasets. As suggested, we have further emphasised this in the revised manuscript to avoid any over-interpretation (e.g. section "Genes are reproducibly ranked by their expression stability in single cells"; first paragraph). (2) We appreciate the suggestion to randomly combine datasets for defining SEGs. However, because these scRNA-seq datasets are generated from using different protocols, platforms and labs and quantified different numbers of genes at different depths in their original experiments, it is challenging to consolidate across all these datasets and normalise the unwanted factors prior to identifying SEGs. Instead, in the revised manuscript, we have incorporated the 'mouse atlas' scRNA-seq data (SMART-Seq2) generated by the Tabula Muris Consortium (Nature 562, 367-372, 2018) for identifying SEGs. Furthermore, the framework for SEG identification is now

Powered by Editorial Manager® and ProduXion Manager® from Aries Systems Corporation

	implemented as 'scSEGIndex' and form part of the scMerge Bioconductor package (https://bioconductor.org/packages/release/bioc/html/scMerge.html), allowing SEGs to be updated when more comprehensive scRNA-seq atlas become available.<br><br>4. Conceptually, SEGs detected by scRNA-seq should (very likely) be subset of HKGs detected by bulk RNA experiments but the reverse is not true. In other words, SEGs from scRNA-seq should be a subset of HKGs. But the currently analysis did not demonstrate this trend. Or do I miss anything? Again, if this is true, it'll be interesting to see combining more datasets will generate better SEGs, as a subset of HKGs.<br><br>Response: We agree with the reviewer that in the ideal world, SEGs should be a subset of HKGs. In fact, the current analysis indeed shows that more than 62% of the human SEGs (i.e. 676/1076) are also found previously as HKGs using bulk RNA experiments. Given the drastic difference in biological technologies and samples, and also different computational methodologies they are derived from, we found such an overlap is quite reassuring.<br><br>5. The authors could perform further analysis on the cross-species comparison. It is only stated that there's high concordance between human and mouse SEGs (Figure 2D, r=0.58). But from the scatter plot with correlation 0.58, it is hard to claim "high concordance". It would be helpful to see if combining more datasets generates higher concordance between human and mouse. This gives some clue whether the difference comes from intrinsic difference across species or because of the need of more datasets.<br><br>Response: As suggested, we have performed additional analyses using SEG index defined from 'mouse atlas' scRNA-seq dataset. These results are included in new Figure 2D of the manuscript. In summary, we didn’t find much difference in concordance between human and mouse gene stability index calculated from using mouse and human development datasets (r=0.58) or using mouse atlas and human development datasets (r=0.54). The concordance of gene stability index calculated from mouse development dataset and mouse atlas is r=0.56. We also performed a permutation test comparing the percentages of SEGs overlapped between human and mouse with null distributions defined using human and mouse genes, respectively (see response 3 to reviewer #1). While we acknowledge the intrinsic difference among species, these results demonstrate that stability of genes in human and mouse are conserved. We have now revised the manuscript to discuss this point (page 3, paragraph 2).<br><br>Minor:<br>1. Similar to the above section (3.3)... (There is no section 3.3)<br><br>Response: We apologise for this typo. We have corrected this in the revised manuscript.<br><br>2. The magnitude in Figure 5A is not very clear. It's more helpful to show full information of p-value and statistics in supplement.<br><br>Response: As suggested, we have increased the size of Figure 5. For the correlation plots (Figure 5A), we have now also included p-value information for those that have a p-value greater than 0.001.
**Additional Information:**	
**Question**	**Response**
Are you submitting this manuscript to a special series or article collection?	No
**Experimental design and statistics**<br><br>Full details of the experimental design and statistical methods used should be given	Yes

in the Methods section, as detailed in our Minimum Standards Reporting Checklist. Information essential to interpreting the data presented should be made available in the figure legends.<br><br>Have you included all the information requested in your manuscript?	
**Resources**<br><br>A description of all resources used, including antibodies, cell lines, animals and software tools, with enough information to allow them to be uniquely identified, should be included in the Methods section. Authors are strongly encouraged to cite Research Resource Identifiers (RRIDs) for antibodies, model organisms and tools, where possible.<br><br>Have you included the information requested as detailed in our Minimum Standards Reporting Checklist?	Yes
**Availability of data and materials**<br><br>All datasets and code on which the conclusions of the paper rely must be either included in your submission or deposited in publicly available repositories (where available and ethically appropriate), referencing such data using a unique identifier in the references and in the “Availability of Data and Materials” section of your manuscript.<br><br>Have you have met the above requirement as detailed in our Minimum Standards Reporting Checklist?	Yes

Manuscript
Click here to access/download;Manuscript;main_v3.pdf
Click here to view linked References

OXFORD (GIGA)^n SCIENCE

GigaScience, 2017, 1–10

doi: xx.xxxx/xxxx
Manuscript in Preparation
Paper

PAPER

Evaluating stably expressed genes in single cells

Yingxin Lin[1], Shila Ghazanfar[1], Dario Strbenac[1], Andy Wang[1,3], Ellis Patrick[1,4], Dave Lin[5], Terence Speed[6,7], Jean YH Yang[1,*] and Pengyi Yang[1,2,*]

[1]School of Mathematics and Statistics, University of Sydney, NSW 2006, Australia and [2]Computational Systems Biology Group, Children's Medical Research Institute, University of Sydney, Westmead, NSW 2145, Australia and [3]Sydney Medical School, University of Sydney, NSW 2006, Australia and [4]Westmead Institute for Medical Research, University of Sydney, Westmead, NSW 2145, Australia and [5]Department of Biomedical Sciences, Cornell University, Ithaca, NY, 14853, USA and [6]Bioinformatics Division, Walter and Eliza Hall Institute of Medical Research, 1G Royal Parade, Parkville, VIC 3052, Australia and [7]Department of Mathematics and Statistics, University of Melbourne, Melbourne, VIC 3010, Australia

[*] pengyi.yang@sydney.edu.au; jean.yang@sydney.edu.au

Abstract

Background: Single-cell RNA-seq (scRNA-seq) profiling has revealed remarkable variation in transcription, suggesting that expression of many genes at the single-cell level are intrinsically stochastic and noisy. Yet, on cell population level, a subset of genes traditionally referred to as housekeeping genes (HKGs) are found to be stably expressed in different cell and tissue types. It is therefore critical to question whether stably expressed genes (SEGs) can be identified on the single-cell level, and if so, how their expression stability can be assessed? We have previously proposed a computational framework for ranking expression stability of genes in single cells for scRNA-seq data normalization and integration. In this study, we perform detailed evaluation and characterization of SEGs derived from this framework.
Results: Here, we show that gene expression stability indices derived from the early human and mouse development scRNA-seq datasets and the 'Mouse Atlas' dataset are reproducible and conserved across species. We demonstrate that SEGs identified from single cells based on their stability indices are considerably more stable than HKGs defined previously from cell populations across diverse biological systems. Our analyses indicate that SEGs are inherently more stable at the single-cell level and their characteristics reminiscent of HKGs, suggesting their potential role in sustaining essential functions in individual cells.
Conclusions: SEGs identified in this study have immediate utility both for understanding variation and stability of single-cell transcriptomes and for practical applications such as scRNA-seq data normalization. Our framework for calculating gene stability index, 'scSEGIndex', is incoporated into the scMerge Bioconductor R package (https://rdrr.io/bioc/scMerge/man/scSEGIndex.html) and can be used for identifying genes with stable expression in scRNA-seq datasets.

Key words: Stably expressed genes; Single cells; scRNA-seq; Housekeeping genes; Gene expression variability

Background

A hallmark of single-cell RNA-seq (scRNA-seq) data has been the remarkable variation in gene transcription that occurs at the level of individual cells [1]. The high degree of variation has led to the appreciation that transcription of genes at the single-cell level are comparatively noisier than on the cell population level [2]. Indeed, a subset of genes are thought to be

Compiled on: May 23, 2019.
Draft manuscript prepared by the author.

Table 1. Summary of scRNA-seq datasets utilized for stably expressed gene identification and/or evaluation in this study.

ID	Publication	Description	Organism	# cell	# class	Protocol	Purpose
E-MTAB-3929	[20]	Human development	Human	1529	5	SMART-Seq2	identify
GSE45719	[21]	Mouse development	Mouse	269	8	SMART-Seq2	identify
GSE109774	[22]	Mouse atlas	Mouse	41965	68	SMART-Seq2	identify
GSE94820	[23]	Peripheral blood mononuclear cells	Human	1140	5	SMART-Seq2	evaluate
GSE75748	[24]	PSCs and endoderm progenitors	Human	1018	7	SMARTer	evaluate
GSE72056	[25]	Multicellular metastatic melanoma	Human	4645	7	SMART-Seq2	evaluate
GSE67835	[26]	Adult and fetal brain	Human	466	8	SMARTer	evaluate
GSE60361	[27]	Cortex and hippocampus	Mouse	3005	7	SMARTer	evaluate
GSE52583	[28]	Developmental lung epithelial cells	Mouse	198	4	SMARTer	evaluate
E-MTAB-4079	[29]	Mesoderm diversification	Mouse	1205	4	SMART-Seq2	evaluate
GSE84133	[30]	Pancreas inter- and intra-cells	Mouse	822	13	InDrop	evaluate

characterized by their stochastic expression [3]. Supporting this notion, genes were found to show transcriptional bursting where their expression varies drastically in individual cells [4, 5]. Furthermore, a large number of genes from scRNA-seq data exhibit bimodality or multimodality of non-zero expression values [6], suggesting that many of these genes may be expressed at different levels in the same and/or different cells. These phenomena illustrate that expression stochasticity is an intrinsic property of many genes on the single-cell level [7].

On the cell population level, however, a subset of genes traditionally referred to as housekeeping genes (HKGs) [8, 9] are found to be stably expressed in different cell types, tissue types and developmental stages [10]. The concept of HKGs is often related to the gene set required to maintain basic cellular functions and therefore is crucial to the understanding of the core transcriptome that is required to sustain life [11, 12, 13]. Early studies such as those by [14], [15], [8], and [16] were conducted to define HKGs using serial analysis of gene expression (SAGE) or microarrays. With the advent of biotechnologies, follow-up studies using more comprehensive data sources such as those by [17] and [18], and high-throughput RNA sequencing (RNA-seq) by [10] and [19], have refined the list of HKGs from populations of cells.

Taken together, the findings from bulk transcriptome data of cell populations and the stochasticity in gene expression observed in individual cells from scRNA-seq data, several fundamental questions arise including (i) Can patterns of stably expressed genes be identified from single cell data? And if so, (ii) how stable are they across individual cells from different tissue types and biological systems? (iii) What properties do such genes have? And (iv) how do they compare to HKGs defined from bulk transcriptome data? In this study, we set out to answer each of these questions.

Leveraging the advances of scRNA-seq techniques [31, 32], we have previously developed a computational framework to rank genes based on various properties extracted from scRNA-seq data to characterize their expression stability in individual cells [33]. These genes were subsequently utilized for scRNA-seq data normalization and integration. To address the questions posed above, here, we applied the proposed framework on two high-resolution scRNA-seq datasets in which a wide range of cell types and developmental stages were profiled in human [20] and mouse [21], and also the 'Mouse Atlas' scRNA-seq dataset that comprenhensively profiled across major mouse orgains and tissue types [22]. We referred to the list of stably expressed genes derived from these datasets as "hSEG" and "mSEG" for human and mouse respectively, and collectively as "SEGs". We subsequently evaluated the stability of SEGs on a collection of independent scRNA-seq datasets generated from diverse tissues and biological systems, and different sequencing protocols. Compared to HKGs previously defined using bulk microarray [16] or RNA-seq datasets [10], SEGs identified on the single-cell level are considerably more stable in all tested biological systems, demonstrating the higher resolution enabled by scRNA-seq data for identifying genes that are truly stably expressed across individual cells, and suggesting their potential roles in maintaining essential functions in individual cells.

Our analyses highlight the previously unappreciated gene stability at the single-cell level. Our computational framework, incoporated as part of the scMerge Bioconductor R package (https://rdrr.io/bioc/scMerge/man/scSEGIndex.html), also allows further identification and refining of SEGs in other scRNA-seq datasets. This will have broad applications in normalization [34, 35] and removal of unwanted variation [36, 37, 33] in scRNA-seq as well as bulk sequencing datasets generated from various experiments.

Data Description

scRNA-seq data processing

A collection of 11 publicly available scRNA-seq datasets (Table 1) were utilized in this study. These datasets were downloaded from either NCBI GEO repository or the EMBL-EBI ArrayExpress repository. Fragments per kilobase of transcript per million (FPKM) values or counts per million (CPM) from their respective original publications were used to quantify full length gene expression for datasets generated by SMARTer or SMART-Seq2 protocols. UMI-filtered counts were used to quantify gene expression for the InDrop dataset. Data were transformed by $\log_2(x + 1)$ where x is the original quantification (e.g. CPM). All datasets have undergone cell-type identification using biological knowledge assisted by various clustering algorithms from their respective original publications which we retain for evaluation purposes. For each dataset, genes with more than 80% missing values (zeros) were removed, with the remaining genes considered as expressed in that dataset. These filtered datasets were used for all subsequent analyses.

Analyses

A computational framework for measuring gene expression stability in single cells

We recently proposed a mixture modelling computational framework for characterising gene expression stability using scRNA-seq data [33]. The orignal framework uses a Gamma component to fit the lower end of the distribution given the non-negative values of gene expression [38] and a Gaussian component to fit the higher end for capturing varibility in cells that express a given gene (Figure 1A). To

test if a Gamma component would be better suited for fitting the higher end of the distribution, here we compared the choices of Gamma-Gaussian and Gamma-Gamma mixture models using Bayesian information criterion (BIC). We found that the Gamma-Gaussian mixture fits consistently better than Gamma-Gamma mixture across multiple datasets (Figure 1B).

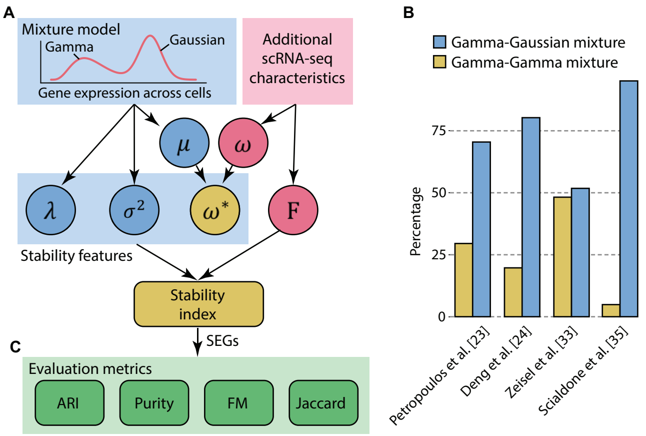

Figure 1. Schematic illustration of the computational framework for deriving gene stability index on the single-cell level. (A) Stability features extracted directly from the mixture model are colored in blue. Those extracted from additional scRNA-seq data characteristics are in red. The overall stability index is derived from the combination of all stability features. (B) Comparison of Gamma-Gaussian and Gamma-Gamma mixture models on four scRNA-seq datasets. *y*-axis represents the percentage of times a given model is selected by Bayesian information criterion (BIC). (C) Evaluation metrics used for evaluating gene expression stability in scRNA-seq datasets.

Using the Gamma-Gaussian mixture model, we extract a set of stability features including λ, σ^2, ω^*, and the F-statistics, and derive a stability index for each gene on the single-cell level. The μ and σ^2 denote the mean and variance of the Gaussian component fitted to a gene x across individual cells. The joint density function $f(.)$ is defined as follows:

$$f(.) = \lambda \frac{\beta^\alpha}{\Gamma(\alpha)} x^{\alpha-1} e^{-\beta x} + (1-\lambda) \frac{1}{\sigma\sqrt{2\pi}} e^{-\frac{(x-\mu)^2}{2\sigma^2}}$$

where $0 \leq \lambda \leq 1$ is the mixing proportion indicating the proportion of cells in the Gamma component in the fitted model. Genes whose expression profiles are with low mixing proportion (λ) and small variance (σ^2) are unimodal and relatively invariant across cells and therefore more likely to be stably expressed.

The ω denotes the percentage of zeros of a gene across cells. The measured expression level for a given gene and cell may be zero due to technical dropout, stochastic expression, or no transcription occurring at all for that gene [39]. Thus, SEG would have relatively small ω (i.e. low proportion of zeros), since they are expected to be expressed in all cells. However, lowly expressed genes tend to have a higher proportion of zeros than highly expressed genes simply due to technical dropouts [40]. We therefore regularized the proportion of zeros (ω) of each gene based on its average expression level μ in the Gaussian component by $\omega^* = \omega \cdot \text{minmax}(\mu)$, where minmax(.) scales the ω^* to the range of 0 to 1. This regularization accounts for the dropout bias towards genes with lower expression.

When pre-defined cell type annotation is available for a given dataset, the F-statistics can be utilized as another stability feature to select for genes in which we observe the same average gene expression across different pre-defined cell types. Together, genes with small λ, σ^2, ω^* and F-statistic are unimodal, expressed with low variance, with relatively low percentage of zeros, and expressed similarly across all cell types, respectively, and are more likely to be stably expressed.

The expression stability index is defined for each gene by combining these four stability features. Specifically, genes are ranked first in increasing order with respect to λ, σ^2, ω^* and F-statistics; and the ranks from each stability features are rescaled to range from 0 to 1. The stability index for each gene is defined as the average of its scaled rankings across all four stability features. Thus, genes are ranked in terms of their degree of evidence towards expression stability in individual cells and can be selected by adjusting the stability index threshold. The subsequent evaluation can be conducted to assess the stability and generalization property of selected SEGs in other biological systems using various evaluation metrics (Figure 1C).

Genes are reproducibly ranked by their expression stability in single cells

To investigate if some genes are inherently more stable in expression on the single-cell level, we utilized three high-resolution scRNA-seq datasets (e.g. human development, mouse development, and the mouse atlas) to quantify genes that are expressed at steady level across different cell types, tissues, and developmental stages of human and mouse, respectively (Table 1; datasets labeled as 'identify'). These datasets provide a starting point for identifying SEGs that can then be used for evaluation on various cell/tissue types and biological systems (Table 1; datasets labeled as 'evaluate').

We first looked at the proportion of zeros per gene across all profiled cells in the early human and mouse development scRNA-seq datasets respectively. We found that a large percentage of genes have more than 50% zero quantification across cells in both datasets (Figure 2A), suggesting most of the genes are transiently expressed in different cell/tissue types and during different developmental stages in both human and mouse. We observed that the distributions of stability features across the three scRNA-seq datasets are different (Figure 2B). Nevertheless, our rank-based approach scales ranks of genes with respect to each stability feature and derives highly comparable stability index distributions from each dataset (Figure 2B, bottom right panel).

We next investigated the reproducibility of the stability index by randomly sampling 80% of all cells from the human and mouse development datasets and re-calculating the stability index for each sub-sample. We found the stability index to be highly reproducible (Figure 2C) within a dataset with average Pearson correlation coefficients of 0.98 and 0.97. In comparison, the correlation of stability indices from the mouse development and mouse altas datasets are much moderate (Figure 2D), suggesting room for further improvement when more comrenhsensive and deeper scRNA-seq datasets become available. We also observed that the stability indices derived for human and mouse are significantly correlated (Figure 2D).

Comparative analysis of SEGs identified in single cells and HKGs defined from bulk transcriptome

To understand the relationships of genes with stable expression in single cells with HKGs defined previously with bulk microarray [16] and RNA-seq [10], we derived a list of SEGs for human and mouse respectively by computing the rank percentiles of stability index as well as the four stability features. Genes with a stability index rank percentile above 80 as well as a reversed rank percentile above 60 for each of the four stability features were included in the SEG list. For mouse, we took the union of the SEGs identified from the mouse develop-

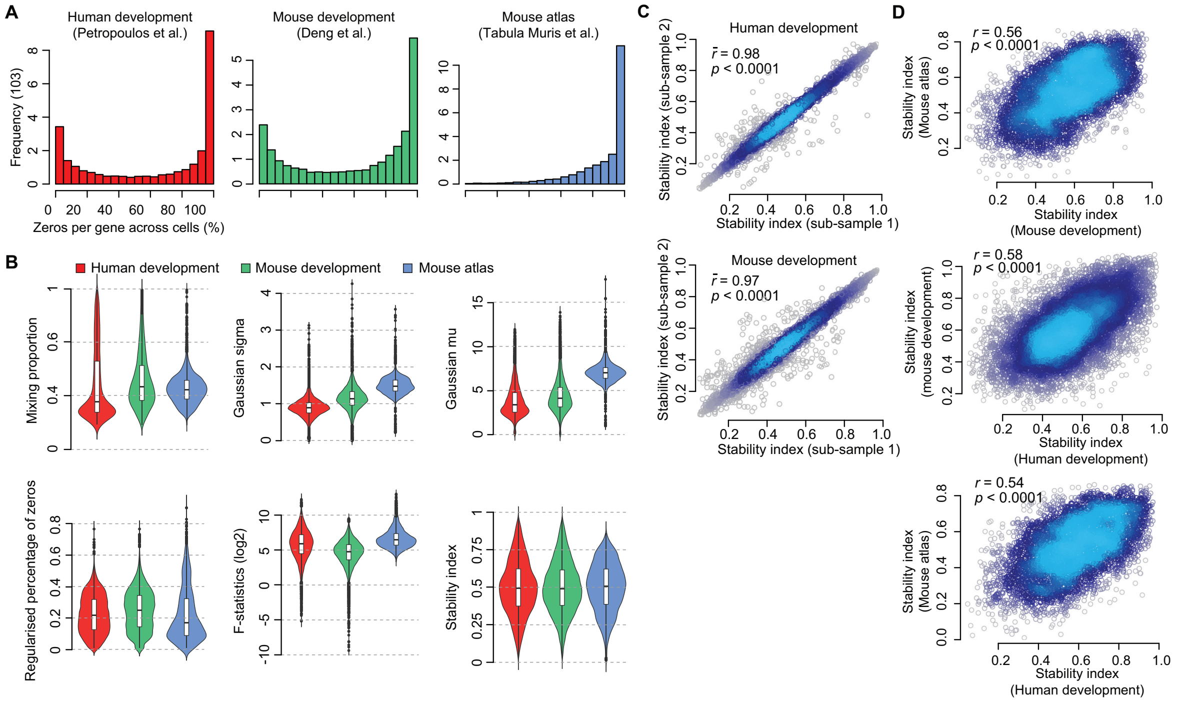

Figure 2. Characterizing gene stability features in single cells for human and mouse. (A) Percentage of zeros per gene across individual cells. (B) Fitted values of mixing proportion (λ), and variance (σ^2) and mean (μ) in the Gaussian component (top panels) of the mixture model for each gene. Regularized percentage of zeros, F-statistics computed from pre-defined cell class (bottom left panel) and stability index derived for each gene (bottom right panel), respectively. (C) Scatter plot of stability index calculated from two random sub-sampling of cells from human and mouse development datasets. Mean Pearson's correlation coefficient ($\bar{r}$) were calculated from pairwise comparison of 10 repeated random sub-sampling on each dataset. (D) Scatter plot and correlation of stability indics calculated from each of three dataset. p-values denote t-distribution test on Pearson's correlation coefficient.

ment and mouse atlas datasets. This resulted in lists of 1076 human (hSEG) and 916 mouse (mSEG) genes, respectively (Figure 3A and B). In comparison to the HKGs defined previously using bulk transcriptomes, we found that hSEG identified on the single-cell level have significantly smaller expression variances across individual cells (Figure 3A).

Comparing with previously defined HKGs (Figure 3C), there were 676 common genes between our hSEG list and those defined by microarray or bulk RNA-seq. This accounts for 62% of hSEGs, a statistically significant overlap (permutation $p < 2e-5$), highlighting a high level of commonality but also uniqueness of SEGs. For the human and mouse SEG lists derived from scRNA-seq datasets, there were 272 common genes (Figure 3D) which accounts for a significant portion of genes in both lists (25% w.r.t hSEG and 30% w.r.t mSEG; permutation $p < 2e-5$), in agreement with the correlation analysis (Figure 2D), suggesting their conservation between human and mouse.

To investigate the difference between SEGs and HKGs defined by bulk transcriptomes, we inspected a few individual genes that were defined as SEGs using scRNA-seq data but not HKGs by bulk microarray or RNA-seq, and *vice versa*. We discovered that many ribosomal proteins (such as *RPL26* and *RPL36*) that were included in the SEG list but not in the HKG lists (Figure 3E) showed strong unimodal expression patterns across all cells. In contrast, genes such as *HINT1* (Histidine triad nucleotide-binding protein 1) and *AGPAT1* (1-Acylglycerol-3-Phosphate O-Acyltransferase), both of which have been reported to be differentially expressed in brain tissue [42] or malignant oesophageal tissues [43] compared to normal samples, were included in both microarray and RNA-seq defined HKG lists, but not in SEG list due to their bimodal expression patterns across individual cells.

Finally, we examined the expression patterns of *GAPDH* and *ACTB* (Figure 3F), genes which are commonly treated as canonical HKGs for data normalization, and observed clear bimodality in both the human and mouse data. In agreement with previous studies [10, 17, 34, 44], these data argue against their usage as "housekeeping genes" for sample normalization.

SEGs exhibit strong expression stability in single cells across different tissues and biological systems

We hypothesized that if the expression levels of the SEGs are relatively stable, they should show relatively small expression differences across the different cell types from various biological systems. We first investigated principal component analysis (PCA) plots generated from early human and mouse development data using all genes (all expressed mRNA), or subsets of genes defined for human (i.e. HKG microarray, HKG RNA-seq, and hSEG) (Figure 4A) and mouse (i.e. mSEG) (Figure 4B). We found that for human data there is clear separation of developmental stages in the first two principal components when PCA plots were created by using either all genes, and HKGs defined from microarray or RNA-seq, suggesting genes that were expressed differentially in different developmental stages were driving the separation. In contrast, the PCA plot generated from using hSEG show much less separation with respect to the developmental stages, suggesting they are generally expressed at a similar level across individual cells irrespective to cell differentiation and change of developmental stages. Similar results were observed from mouse development data (Figure 4B) where PCA plot generated from mSEG show less cell type and development stage separation compared to PCA plot generated from using all genes.

To quantify the above visual observations in human and mouse developmental datasets, we utilized k-means clustering to partition cells into five and eight clusters respectively, using all genes (all expressed mRNA) or subsets of genes defined in each list (i.e. hSEG, mSEG, HKG microarray and HKG RNA-

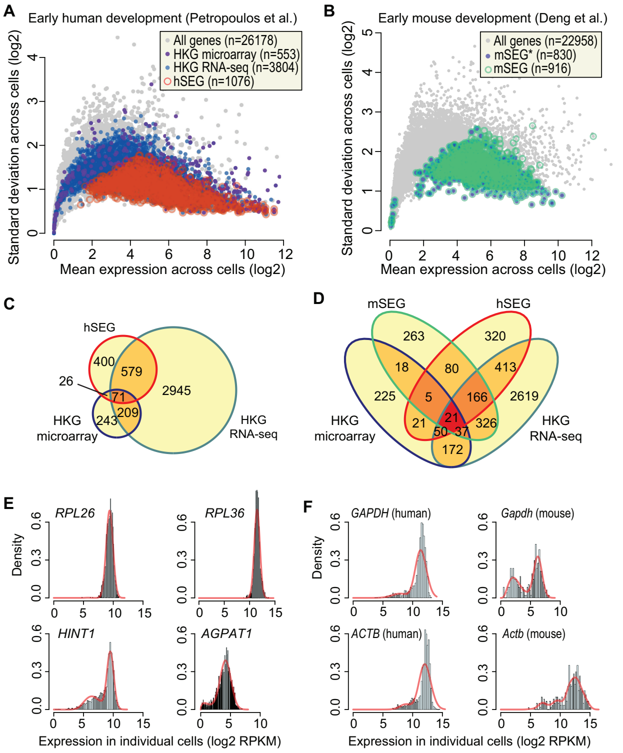

Figure 3. Comparison of SEGs identified on individual cell level using scRNA-seq with HKGs defined on cell population level using bulk transcriptome data. (A) Scatter plot showing mean expression (x-axis) and variance (y-axis) of each gene (gray circles) across profiled single cells. Open red circles represent SEGs identified from early human development data (hSEG) in this study whereas dark and light blue solid circles represent HKGs defined previously using bulk microarray [16] and RNA-seq data [10]. (B) Same as (A) but for SEGs identified from early mouse development data (mSEG*; light blue points) and the union of these identified from both mouse development and mouse altas datasets (mSEG; green circles). (C) Venn diagrams showing overlaps of hSEGs and HKGs defined using bulk microarray and RNA-seq. (D) Overlap of all human and mouse gene lists (E). (E) Expression patterns of example genes that are defined as SEGs using scRNA-seq data but not as HKGs using bulk microarray or RNA-seq data (*RPL*26 and *RPL*36) and vice versa (*HINT* and *AGPAT*1) across individual cells. (F) Expression patterns for *GAPDH* and *ACTB* in human and mouse (*Gapdh* and *Actb*) across individual cells.

seq) with the hypothesis that clusters arising from using SEGs and HKGs will exhibit lower concordance with pre-defined cell type- and tissue-specific labels (Figure 4C), thereby demonstrating consistent levels of expression across different cell and tissue types. To account for the size difference of the gene lists, we also created subsets of HKGs identified from RNA-seq data (sub HKG RNA-seq) to match the sizes of hSEGs and mSEGs, respectively; and subsets of hSEGs (sub hSEG) and mSEGs (sub mSEG) to match the size of HKGs identified from microarray data.

We found that k-means clustering outputs using SEGs derived from scRNA-seq data showed the lowest concordance to their pre-defined cell class labels (i.e. embryonic day of development or cell types) as quantified by the adjusted rand index (ARI), Purity, Fowlkes-Mallows index (FM), and Jaccard index (Figure 4D). The reduction of either list to match the other had relatively minor effect on the clustering results. These results demonstrate that SEGs are stably expressed across cells and developmental stages in the two scRNA-seq datasets.

To test whether SEGs derived above are stably expressed in other cell and tissue types, we evaluated these SEGs and their subsets that matched the size of HKGs defined from microarray data on eight datasets (Table 1) which are independent of the scRNA-seq datasets used for identifying SEGs. These additional datasets represent drastically different tissues and biological systems in both human and mouse, as well as different sequencing protocols and a wide range in the number of cells sequenced.

Similar to the above section, we quantified the clustering concordance with respect to each of their pre-defined cell class labels using each of the four concordance metrics (ARI, Purity, FM, and Jaccard) (Table 2). We found that on average, clustering using SEGs (and their subsets) gave the lowest concordance to the pre-defined cell type- and tissue-specific class labels in all tested datasets compared to clustering using all expressed genes or HKGs defined using bulk microarray and RNA-seq datasets. These results suggest that SEGs defined in early human and mouse development also display strong expression stability in various cell/tissue types and biological systems, and they are considerably more stable than HKGs defined using bulk transcriptome data on the single-cell level.

Gene stability index derived from single cells correlates with gene sequence and structural characteristics

To further characterize gene expression stability in single cells, we correlated the stability index and each stability feature extracted from scRNA-seq data with various gene structural and conservation features calculated from various data sources. We found that the stability index correlated positively with the number of exons in a gene, gene expression, and gene conservation, and negatively with GC-content in the gene body in both human and mouse (Figure 5A), many of which are characteristics of HKGs reported in previous studies. Consistent with this, we found SEGs are more evolutionarily conserved [45] with higher phyloP scores. SEGs also possess more exons, in agreement with previous finding on HKGs [46], despite mouse genes on average having fewer exons than human genes. Both human and mouse SEGs appeared to have a slightly lower GC-content but, similar to previous observation on HKGs, the relation was relatively weak [47] (Figure 5B).

Perhaps unsurprisingly, SEGs identified in this study possess similar characteristics to those observed in HKGs, indicating that they are serving essential cellular functions akin to HKGs. Supporting this, we found that multiple top-enriched Gene Ontology and Reactome terms that describe essential cellular functions are shared by common SEGs (genes overlap between hESG and mSEG) and common HKGs (genes overlap between HKG microarray and HKG RNA-seq) (Figure 5C) (see Methods for details). Nevertheless, common SEGs are far more enriched for most GO and Reactome terms than common HKGs defined from bulk transcriptome and also show significantly higher conservation in both human and mouse (Figure 5D). These results indicate the higher resolution enabled by scRNA-seq data for identifying genes that are truly stably expressed across individual cells.

Discussion

Since the emergence of high-throughput transcriptome profiling, the search for stably expressed genes (SEGs) has been a central quest in modern biology. Such genes are often thought to be essential for basic cellular functions given their relatively constant expression and activity despite changes in cell status and types. The hypothesis that such genes may serve the same housekeeping functions across various cell and tissue types has also led to their definition as "housekeeping genes" (HKGs). While the existence of true HKGs whose expression are universally constant across all cells and systems is a subject of debate [43, 48], their practical usage as control genes for experimental

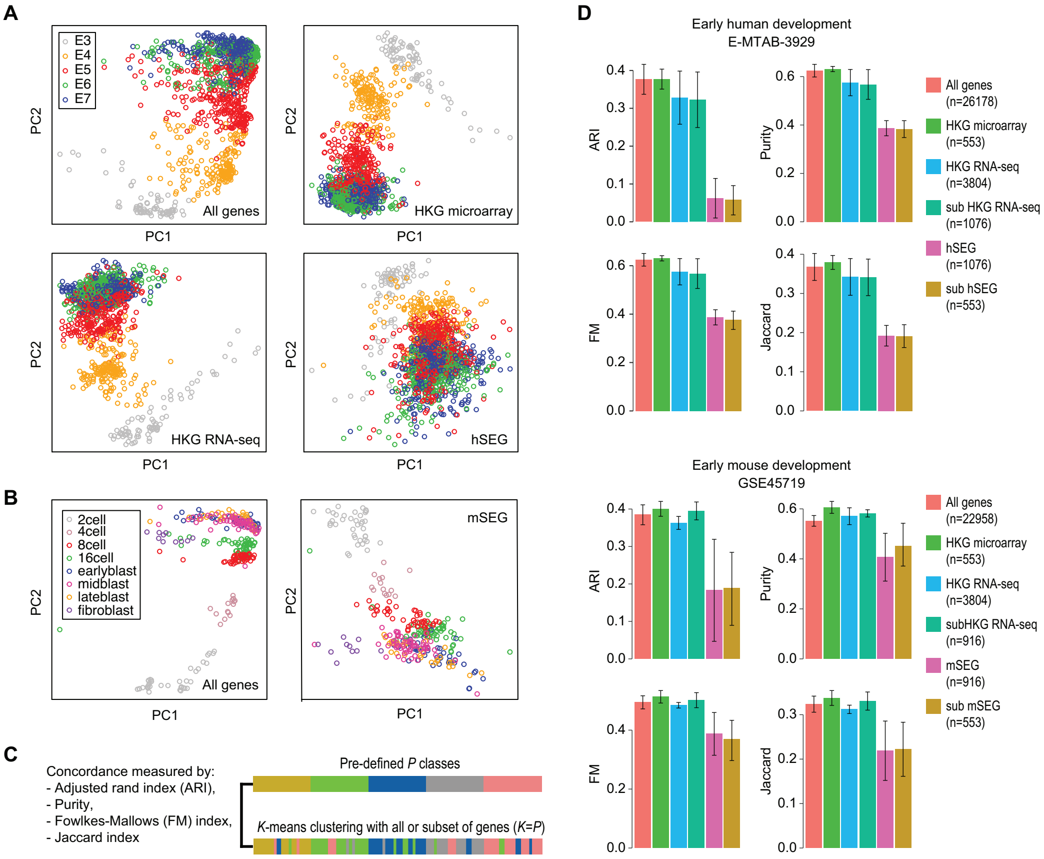

Figure 4. Stability of SEGs and HKGs in human and mouse development scRNA-seq datasets. (A) PCA plots generated from human development data using all expressed genes, HKGs, or hSEGs. Cells are colored by their pre-defined developmental stages. (B) PCA plots generated from mouse development data using all expressed genes or mSEGs. Cells are colored by their pre-defined types and developmental stages. (C) Schematic showing the quantification of concordance of *k*-means clustering with pre-defined cell classes using a panel of metrics. (D) Barplots of concordance between *k*-means clustering and pre-defined cell class labels, using all expressed genes, HKGs identified from microarray and RNA-seq data, SEGs identified from this study for human (hSEGs) and mouse (mSEGs), and size matched subset of HKGs to SEGs and vice versa.

Table 2. Stability evaluation results on independent scRNA-seq datasets that profile various cell types and biological systems. All indices are within the range of [0, 1] and are multiplied by 100.

	Peripheral blood mononuclear cells (human); [23]					PSCs and endoderm progenitors (human); [24]				
	All genes	HKG		SEG		All genes	HKG		SEG	
		Array	RNA-seq	n=1076	n=553		Array	RNA-seq	n=1076	n=553
ARI	55±8	42±3	38±4	29±6	21±3	69±5	58±5	55±6	41±3	40±3
Purity	69±7	62±2	59±1	52±5	48±5	80±4	74±3	71±5	59±3	61±4
FM	67±5	56±1	52±3	45±4	40±2	75±4	66±4	63±5	51±2	50±3
Jaccard	49±6	39±1	35±2	29±4	25±2	60±5	48±4	46±6	34±2	33±2
	Multicellular metastatic melanoma (human); [25]					Adult and fetal brain (human); [26]				
	All genes	HKG		SEG		All genes	HKG		SEG	
		Array	RNA-seq	n=1076	n=553		Array	RNA-seq	n=1076	n=553
ARI	31±5	18±2	18±1	15±1	15±1	53±7	50±3	39±4	36±3	34±3
Purity	80±5	73±1	74±1	71±1	70±1	82±3	76±4	74±3	68±2	65±1
FM	51±3	39±2	40±1	37±1	36±1	62±6	59±2	50±3	47±3	46±3
Jaccard	32±2	22±2	24±1	21±1	20±0	44±6	41±2	33±3	30±3	29±2
	Cortex and hippocampus (mouse); [27]					Developmental lung epithelial cells (mouse); [28]				
	All genes	HKG		SEG		All genes	HKG		SEG	
		Array	RNA-seq	n=916	n=553		Array	RNA-seq	n=916	n=553
ARI	45±8	36±5	31±3	28±3	26±2	61±6	55±4	48±2	46±0	43±5
Purity	72±3	66±1	63±1	59±1	58±2	83±4	80±2	76±1	75±0	73±3
FM	55±6	49±4	44±3	42±2	40±2	72±4	68±3	62±2	61±0	59±4
Jaccard	38±6	32±4	28±2	26±2	25±2	56±5	51±3	45±2	44±0	42±4
	Mesoderm diversification (mouse); [29]					Pancreas inter- and intra-cells (mouse); [30]				
	All genes	HKG		SEG		All genes	HKG		SEG	
		Array	RNA-seq	n=916	n=553		Array	RNA-seq	n=916	n=553
ARI	54±2	43±8	49±3	31±7	10±7	37±4	22±3	23±3	19±2	17±3
Purity	66±1	62±6	65±1	59±7	48±7	89±3	78±3	76±2	74±2	71±2
FM	68±1	63±8	67±1	59±7	53±5	52±4	38±3	39±3	35±2	32±3
Jaccard	52±1	46±7	50±1	40±8	32±7	30±3	20±3	21±3	17±2	16±2

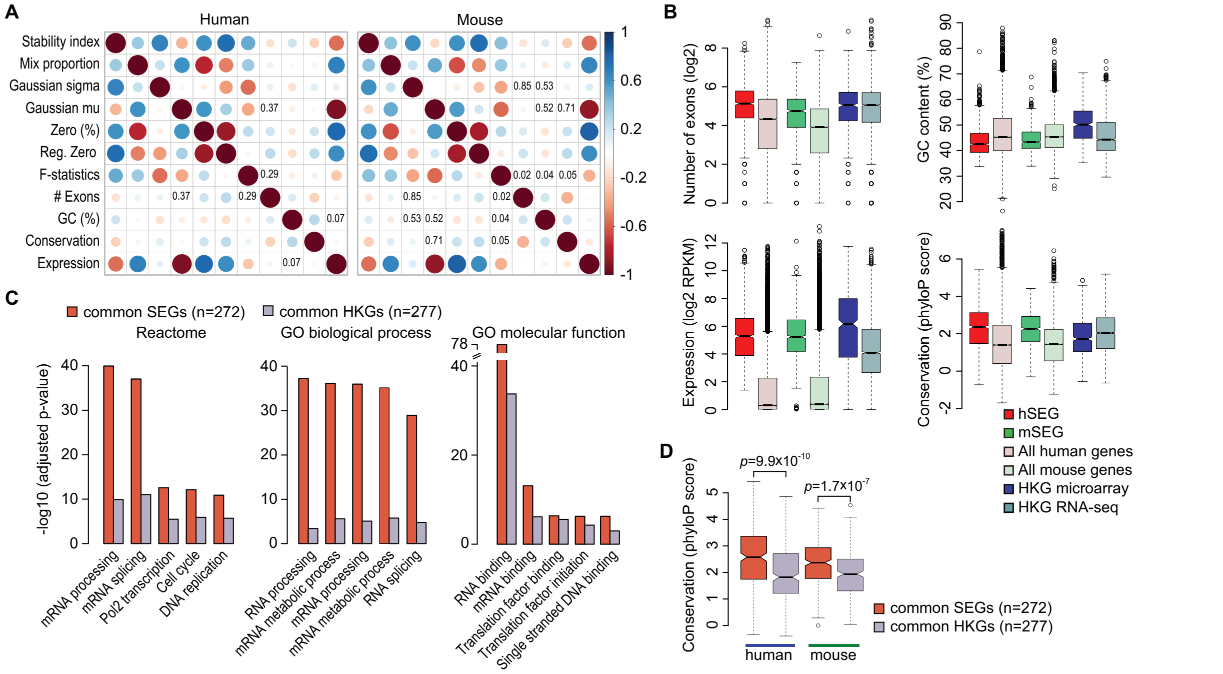

Figure 5. Characterization of stability index with sequence and gene characteristics. (A) Pearson correlation analyses of human and mouse gene stability features with respect to genomic structural and evolutional gene features. p-values greater than 0.001 are displayed. (B) Boxplots of various gene characteristics for SEGs, HKGs and all expressed genes. (C) Over-representation analyses of SEGs that are common between hESG and mSEG (common SEGs); and HKGs that are common between HKG microarray and HKG RNA-seq (common HKGs), using Gene Ontology (GO) and Reactome databases. (D) Comparison of conservation for common SEGs and common HKGs in human and mouse genomes.

data normalization is well appreciated.

Recent advances in single-cell transcriptome profiling using scRNA-seq have highlighted the phenomenal amount of gene expression stochasticity and heterogeneity in single cells. Compared to bulk transcriptome data that aggregate millions of cells to obtain a single gene expression measure, scRNA-seq data allows the expression dynamics of each gene within individual cells to be monitored, and therefore enables the identification of genes that are truly expressed at a steady level in individual cells across tissues and developmental stages. By modeling from large-scale scRNA-seq datasets, we quantified the relative expression stability of genes on the single-cell levels. We showed that the SEGs derived based on their stability indices are considerably more stable in not only the scRNA-seq datasets from which they are identified but also independent scRNA-seq datasets that profiles various cell types and biological systems.

Our analysis demonstrated that despite the high variability in single-cell gene expression, a subset of genes is inherently more stable in expression than other genes within individual cells. Their sequence and gene structural properties are strongly reminiscent of HKGs defined from bulk transcriptome, suggesting their essential roles in maintaining basic cellular functions on the individual cell level.

The proposed framework can be applied in a data dependent manner to rank genes based on their expression stability in a given scRNA-seq dataset. This relaxes the rigid binary definition of HKGs and enable a more practical definition of stable expression in different experimental contexts. Hence, the proposed method is particularly useful for defining stable or "control" genes in various scRNA-seq experiment, which is often a key step in normalizing such data [49, 50]. Indeed, the utility of SEGs on scRNA-seq data normalization has already been demonstrated by our recent study on integrating multiple scRNA-seq datasets [33].

The generalizability of SEGs is dependent on the diversity of cell types profiled in a scRNA-seq experiment. Various cell atlas profiling initiatives such as the Human Cell Atlas (https://www.humancellatlas.org) is currently under way to comprehensively characterize the transcriptome of every human cell. Information from such resources in conjunction with our computational framework will provide an even more precise assessment of gene expression stability in single cells that will enrich subsequent avenues of research including characterizing heterogeneity and stability of single-cell transcriptomes and their use for technical data normalization and standardization.

Taken together, this comprehensive evaluation study demonstrates the utility of measuring gene expression stability at the single-cell level and marks a shift in paradigm for selecting genes that are stably expressed in single cells for practical applications.

Methods

Evaluating the stability of gene lists

To assess the expression stability of each gene list in various cell types and biological systems, the k-means algorithm was utilized to cluster each scRNA-seq data to its pre-defined number of clusters and an array of evaluation metrics were applied to compute the concordance with respect to the pre-defined ("gold standard") class labels. Evaluation metrics include the adjusted Rand index (ARI), Purity, the Fowlkes-Mallows index (FM) and the Jaccard index.

Let $U = \{u_1, u_2, \dots, u_P\}$ denote the true partition across P classes and $V = \{v_1, v_2, \dots, v_K\}$ denote the partition produced from k-means clustering ($K = P$). Let a be the number of pairs of cells correctly partitioned into the same class by the clustering method; b be the number of pairs of cells partitioned into the same cluster but in fact belong to different classes; c be the number of pairs of cells partitioned into different clusters but belongs to the same class; and d be the number of

pairs of cells correctly partitioned into different clusters. Then the Adjusted Rand Index [51], the Jaccard index [52], and the Fowlkes-Mallows index [53] can be defined as

$$\text{ARI} = \frac{2(ad - bc)}{(a+b)(b+d) + (a+c)(c+d)};$$

$$\text{Jaccard} = a/(a+b+c);$$

$$\text{FM} = \sqrt{\left(a/(a+b)\right)\left(a/(a+c)\right)};$$

and the Purity [54] can be calculated as

$$\text{Purity} = \frac{1}{N}\sum_i \max_j |u_i \cap v_j|$$

where N is the total number of cells, i and j are the indices of clusters from clustering output u_i and pre-defined class label v_j.

For each dataset, we calculated and compared the above four metrics using (i) all expressed genes, (ii) HKGs defined using microarray data [16], (iii) HKGs defined using bulk RNA-seq data [10], and (iv) SEGs identified in this study. In order to account for potential effects of gene list length, we also generated random subsets with the same number of genes in our SEG lists first by randomly sampling from all expressed genes in the dataset, and second by randomly sampling from the HKG list defined by bulk RNA-seq. Since the k-means clustering algorithm is not deterministic and the random sampling process introduces variability, the above procedure was repeated 10 times to account for such variability.

Gene properties

To characterize SEGs identified in early human and mouse development datasets, we extracted gene sequence and structural features including the number of exons and percentage GC content in the gene body for human and mouse, respectively, using the biomaRt [55]. Additionally, to characterize gene evolutionary conservation, phyloP scores were downloaded from the UCSC Genome Browser for mouse (mm10) and human (hg38) genomes. Exonic bases of each gene were determined based on GENCODE Genes for human (release 26) and mouse (release 14). The set of conservation scores for each gene was averaged for each gene. We assessed the concordance of gene expression stability index and each stability feature derived from single cells with structural features, conservation scores, and their expression across all genes for human and mouse using Pearson correlation coefficients. We also compared these features for SEGs and previously defined HKGs against all expressed genes in human and mouse, respectively.

Gene ontology enrichment analysis

To perform gene ontology enrichment analysis, we first defined SEGs that are shared between hSEG and mSEG as “common SEGs" and HKGs that are shared between HKG microarray and HKG RNA-seq as “common HKGs". The similar numbers of common SEGs (256) and common HKGs (277) allowed us to avoid the gene-set size bias in the enrichment analysis.

Over-representation of common SEGs or common HKGs was evaluated by comparing each set of genes against ontologies defined in Gene Ontology database [56] and those defined in Reactome database [?]. Fisher’s exact test was used to assess statistical significance. Top-enriched ontologies from either common SEGs or common HKGs were combined for interpretation.

Availability of supporting data and materials

The datasets generated and/or analyzed during the current study are available in either the NCBI GEO repository or the EMBL-EBI ArrayExpress repository (Table 1). The computational framework for calculating gene stability index, ‘scSEGIndex’, is available from (https://rdrr.io/bioc/scMerge/man/scSEGIndex.html).

Declarations

List of abbreviations

scRNA-seq: Single-cell RNA-seq; HKGs: housekeeping genes; SEGs: stably expressed genes; SAGE: serial analysis of gene expression; hSEG: stably expressed genes derived from early human developmental dataset; mSEG: stably expressed genes derived from early human developmental dataset; HKG microarray: housekeeping genes defined using bulk microarray; HKG RNA-seq: housekeeping genes defined using bulk RNA-seq; PCA: principal component analysis; ARI: adjusted rand index; FM: Fowlkes-Mallows index.

Consent for publication

Not applicable

Competing Interests

The author(s) declare that they have no competing interests.

Funding

This work is supported by Australian Research Council (ARC)/Discovery Early Career Researcher Award (DE170100759) to P.Y., National Health and Medical Research Council (NHMRC)/Career Development Fellowship (1105271) to J.Y.H.Y., ARC/Discovery Project (DP170100654) grant to P.Y. and J.Y.H.Y., and NHMRC/Program Grant (1054618) to T.P.S.

Author’s Contributions

PY conceived the study with input from JYHY. All authors contributed to the design, analytics, interpretation and the direction of the study. YL and PY lead the analytics and AYW lead the curation of the datasets. All authors wrote, reviewed, edited, and approved the final version of the manuscript.

Acknowledgements

The authors thank their colleagues at the School of Mathematics and Statistics, The University of Sydney, and Prof. Ze-Guang Han and Dr. Xianbin Su at Shanghai Jiao Tong University for informative discussion and valuable feedback.

References

1. Martinez-Jimenez CP, Eling N, Chen HC, Vallejos CA, Kolodziejczyk AA, Connor F, et al. Aging increases cell-to-cell transcriptional variability upon immune stimulation. Science 2017;355(6332):1433–1436.
2. Marinov GK, Williams BA, McCue K, Schroth GP, Gertz J, Myers RM, et al. From single-cell to cell-pool transcriptomes: stochasticity in gene expression and RNA splicing. Genome Research 2014;24(3):496–510.
3. Kolodziejczyk AA, Kim JK, Svensson V, Marioni JC, Teichmann SA. The technology and biology of single-cell RNA sequencing. Molecular Cell 2015;58(4):610–620.
4. Suter DM, Molina N, Gatfield D, Schneider K, Schibler U, Naef F. Mammalian genes are transcribed with widely different bursting kinetics. Science 2011;332(6028):472–474.
5. Fukaya T, Lim B, Levine M. Enhancer control of transcriptional bursting. Cell 2016;166(2):358–368.
6. Shalek AK, Satija R, Adiconis X, Gertner RS, Gaublomme JT, Raychowdhury R, et al. Single-cell transcriptomics reveals bimodality in expression and splicing in immune cells. Nature 2013;498(7453):236.
7. Kærn M, Elston TC, Blake WJ, Collins JJ. Stochasticity in gene expression: from theories to phenotypes. Nature Reviews Genetics 2005;6(6):451.
8. Hsiao LL, Dangond F, Yoshida T, Hong R, Jensen RV, Misra J, et al. A compendium of gene expression in normal human tissues reveals tissue-selective genes and distinct expression patterns of housekeeping genes. Physiological Genomics 2001;.
9. Butte AJ, Dzau VJ, Glueck SB. Further defining housekeeping, or "maintenance," genes Focus on "A compendium of gene expression in normal human tissues". Physiological Genomics 2001;7(2):95–96.
10. Eisenberg E, Levanon EY. Human housekeeping genes, revisited. Trends in Genetics 2013;29(10):569–574.
11. Koonin EV. Comparative genomics, minimal gene-sets and the last universal common ancestor. Nature Reviews Microbiology 2003;1(2):127.
12. Gil R, Silva FJ, Peretó J, Moya A. Determination of the core of a minimal bacterial gene set. Microbiology and Molecular Biology Reviews 2004;68(3):518–537.
13. Glass JI, Assad-Garcia N, Alperovich N, Yooseph S, Lewis MR, Maruf M, et al. Essential genes of a minimal bacterium. Proceedings of the National Academy of Sciences 2006;103(2):425–430.
14. Velculescu VE, Madden SL, Zhang L, Lash AE, Yu J, Rago C, et al. Analysis of human transcriptomes. Nature Genetics 1999;23(4):387.
15. Warrington J, Nair A, Mahadevappa M, Tsyganskaya M. Comparison of human adult and fetal expression and identification of 535 housekeeping/maintenance genes. Physiological Genomics 2000;2(3):143–147.
16. Eisenberg E, Levanon EY. Human housekeeping genes are compact. Trends in Genetics 2003;19(7):362–365.
17. De Jonge HJ, Fehrmann RS, de Bont ES, Hofstra RM, Gerbens F, Kamps WA, et al. Evidence based selection of housekeeping genes. PloS One 2007;2(9):e898.
18. Zhu J, He F, Song S, Wang J, Yu J. How many human genes can be defined as housekeeping with current expression data? BMC Genomics 2008;9(1):172.
19. Ramsköld D, Wang ET, Burge CB, Sandberg R. An abundance of ubiquitously expressed genes revealed by tissue transcriptome sequence data. PLoS Computational Biology 2009;5(12):e1000598.
20. Petropoulos S, Edsgärd D, Reinius B, Deng Q, Panula SP, Codeluppi S, et al. Single-cell RNA-seq reveals lineage and X chromosome dynamics in human preimplantation embryos. Cell 2016;165(4):1012–1026.
21. Deng Q, Ramsköld D, Reinius B, Sandberg R. Single-cell RNA-seq reveals dynamic, random monoallelic gene expression in mammalian cells. Science 2014;343(6167):193–196.
22. Consortium TM, et al. Single-cell transcriptomics of 20 mouse organs creates a Tabula Muris. Nature 2018;562:367–372.
23. Villani AC, Satija R, Reynolds G, Sarkizova S, Shekhar K, Fletcher J, et al. Single-cell RNA-seq reveals new types of human blood dendritic cells, monocytes, and progenitors. Science 2017;356(6335):eaah4573.
24. Chu LF, Leng N, Zhang J, Hou Z, Mamott D, Vereide DT, et al. Single-cell RNA-seq reveals novel regulators of human embryonic stem cell differentiation to definitive endoderm. Genome Biology 2016;17(1):173.
25. Tirosh I, Izar B, Prakadan SM, Wadsworth MH, Treacy D, Trombetta JJ, et al. Dissecting the multicellular ecosystem of metastatic melanoma by single-cell RNA-seq. Science 2016;352(6282):189–196.
26. Darmanis S, Sloan SA, Zhang Y, Enge M, Caneda C, Shuer LM, et al. A survey of human brain transcriptome diversity at the single cell level. Proceedings of the National Academy of Sciences 2015;112(23):7285–7290.
27. Zeisel A, Muñoz-Manchado AB, Codeluppi S, Lönnerberg P, La Manno G, Juréus A, et al. Cell types in the mouse cortex and hippocampus revealed by single-cell RNA-seq. Science 2015;347(6226):1138–1142.
28. Treutlein B, Brownfield DG, Wu AR, Neff NF, Mantalas GL, Espinoza FH, et al. Reconstructing lineage hierarchies of the distal lung epithelium using single-cell RNA-seq. Nature 2014;509(7500):371.
29. Scialdone A, Tanaka Y, Jawaid W, Moignard V, Wilson NK, Macaulay IC, et al. Resolving early mesoderm diversification through single-cell expression profiling. Nature 2016;535(7611):289.
30. Baron M, Veres A, Wolock SL, Faust AL, Gaujoux R, Vetere A, et al. A single-cell transcriptomic map of the human and mouse pancreas reveals inter-and intra-cell population structure. Cell Systems 2016;3(4):346–360.
31. Tang F, Barbacioru C, Wang Y, Nordman E, Lee C, Xu N, et al. mRNA-Seq whole-transcriptome analysis of a single cell. Nature Methods 2009;6(5):377.
32. Jaitin DA, Kenigsberg E, Keren-Shaul H, Elefant N, Paul F, Zaretsky I, et al. Massively parallel single-cell RNA-seq for marker-free decomposition of tissues into cell types. Science 2014;343(6172):776–779.
33. Lin Y, Ghazanfar S, Wang KYX, Gagnon-Bartsch JA, Lo KK, Su X, et al. scMerge leverages factor analysis, stable expression, and pseudoreplication to merge multiple single-cell RNA-seq datasets. Proceedings of the National Academy of Sciences 2019;116(20):9775–9784. https://www.pnas.org/content/116/20/9775.
34. Thellin O, Zorzi W, Lakaye B, De Borman B, Coumans B, Hennen G, et al. Housekeeping genes as internal standards: use and limits. Journal of Biotechnology 1999;75(2-3):291–295.
35. Robinson MD, Oshlack A. A scaling normalization method for differential expression analysis of RNA-seq data. Genome Biology 2010;11(3):R25.
36. Risso D, Ngai J, Speed TP, Dudoit S. Normalization of RNA-seq data using factor analysis of control genes or samples. Nature Biotechnology 2014;32(9):896.
37. Gagnon-Bartsch JA, Speed TP. Using control genes to correct for unwanted variation in microarray data. Biostatistics 2012;13(3):539–552.
38. Ghazanfar S, Bisogni AJ, Ormerod JT, Lin DM, Yang JY. Integrated single cell data analysis reveals cell specific net-

works and novel coactivation markers. BMC Systems Biology 2016;10(5):127.
39. Kharchenko PV, Silberstein L, Scadden DT. Bayesian approach to single-cell differential expression analysis. Nature Methods 2014;11(7):740.
40. Bhargava V, Head SR, Ordoukhanian P, Mercola M, Subramaniam S. Technical variations in low-input RNA-seq methodologies. Scientific reports 2014;4:3678.
41. Cockburn K, Rossant J. Making the blastocyst: lessons from the mouse. The Journal of clinical investigation 2010;120(4):995–1003.
42. Varadarajulu J, Schmitt A, Falkai P, Alsaif M, Turck CW, Martins-de Souza D. Differential expression of HINT1 in schizophrenia brain tissue. European Archives of Psychiatry and Clinical Neuroscience 2012;262(2):167–172.
43. Rubie C, Kempf K, Hans J, Su T, Tilton B, Georg T, et al. Housekeeping gene variability in normal and cancerous colorectal, pancreatic, esophageal, gastric and hepatic tissues. Molecular and Cellular Probes 2005;19(2):101–109.
44. Suzuki T, Higgins P, Crawford D, et al. Control selection for RNA quantitation. Biotechniques 2000;29(2):332–337.
45. Zhang L, Li WH. Mammalian housekeeping genes evolve more slowly than tissue-specific genes. Molecular Biology and Evolution 2004;21(2):236–239.
46. Zhu J, He F, Hu S, Yu J. On the nature of human housekeeping genes. Trends in Genetics 2008;24(10):481–484.
47. Sémon M, Mouchiroud D, Duret L. Relationship between gene expression and GC-content in mammals: statistical significance and biological relevance. Human Molecular Genetics 2004;14(3):421–427.
48. Arukwe A. Toxicological housekeeping genes: do they really keep the house? Environmental Science & Technology 2006;40(24):7944–7949.
49. Bacher R, Chu LF, Leng N, Gasch AP, Thomson JA, Stewart RM, et al. SCnorm: robust normalization of single-cell RNA-seq data. Nature Methods 2017;14(6):584.
50. Lun AT, Bach K, Marioni JC. Pooling across cells to normalize single-cell RNA sequencing data with many zero counts. Genome Biology 2016;17(1):75.
51. Rand WM. Objective criteria for the evaluation of clustering methods. Journal of the American Statistical Association 1971;66(336):846–850.
52. Milligan GW, Cooper MC. A study of the comparability of external criteria for hierarchical cluster analysis. Multivariate Behavioral Research 1986;21(4):441–458.
53. Fowlkes EB, Mallows CL. A method for comparing two hierarchical clusterings. Journal of the American Statistical Association 1983;78(383):553–569.
54. Amigó E, Gonzalo J, Artiles J, Verdejo F. A comparison of extrinsic clustering evaluation metrics based on formal constraints. Information Retrieval 2009;12(4):461–486.
55. Durinck S, Moreau Y, Kasprzyk A, Davis S, De Moor B, Brazma A, et al. BioMart and Bioconductor: a powerful link between biological databases and microarray data analysis. Bioinformatics 2005;21(16):3439–3440.
56. Consortium GO. Expansion of the Gene Ontology knowledgebase and resources. Nucleic Acids Research 2016;45(D1):D331–D338.

Supplementary Table 1

Click here to access/download
Supplementary Material
Supplementary Table 1.xlsx

Response letter including graphical panels

Click here to access/download
Supplementary Material
Response_v3.docx

Personal Cover Click here to access/download;Personal Cover;CoverLetter.pdf

THE UNIVERSITY OF SYDNEY

School of Mathematics and Statistics

Faculty of Science,
Carslaw Building F07,
NSW 2006 AUSTRALIA
Telephone: +61 2 9351 3012
Fax: + 61 2 9351 4533
Email: pengyi.yang@sydney.edu.au

21 May 2019

Dear Editor Zhou,

Please find enclosed our revised manuscript (**GIGA-D-18-00467**).

We are delighted that our manuscript "Evaluating stably expressed genes in single cells" is of interest to *GigaScience*. In light of the comments and suggestions from the two reviewers, we have performed additional experiments and analyses and thoroughly address each of all comments to the best of our ability. These new analyses have allowed us to significantly strengthen the conclusions and improve the scientific values of the revised manuscript.

We hope that our point-by-point response enclosed with the submission adequately addresses any remaining questions. Thank you sincerely for considering our work for publication.

Yours faithfully,

Pengyi Yang and Jean Yang
(on behalf of all authors)
