# Supplementary material for: Evaluating stably expressed genes in single cells
Source: Gigascience. 2019 Sep 16;8(9):giz106. doi: 10.1093/gigascience/giz106 (PMC6748759; doi:10.1093/gigascience/giz106)
Supplement: giz106_GIGA-D-18-00467_Revision_2 [file giz106_giga-d-18-00467_revision_2.pdf]

GigaScience

Evaluating stably expressed genes in single cells

--Manuscript Draft--

**Manuscript Number:**	GIGA-D-18-00467R2
**Full Title:**	Evaluating stably expressed genes in single cells
**Article Type:**	Research
**Funding Information:**	Australian Research Council (DE170100759) — Dr Pengyi Yang<br>National Health and Medical Research Council (1105271) — Prof. Jean YH Yang<br>Australian Research Council (DP170100654) — Prof. Jean YH Yang<br>National Health and Medical Research Council (1054618) — Prof. Terence Speed
**Abstract:**	Background: Single-cell RNA-seq (scRNA-seq) profiling has revealed remarkable variation in transcription, suggesting that expression of many genes at the single-cell level are intrinsically stochastic and noisy. Yet, on the cell population level, a subset of genes traditionally referred to as housekeeping genes (HKGs) are found to be stably expressed in different cell and tissue types. It is therefore critical to question whether stably expressed genes (SEGs) can be identified on the single-cell level, and if so, how can their expression stability be assessed? We have previously proposed a computational framework for ranking expression stability of genes in single cells for scRNA-seq data normalization and integration. In this study, we perform detailed evaluation and characterization of SEGs derived from this framework.<br><br>Results: Here, we show that gene expression stability indices derived from the early human and mouse development scRNA-seq datasets and the 'Mouse Atlas' dataset are reproducible and conserved across species. We demonstrate that SEGs identified from single cells based on their stability indices are considerably more stable than HKGs defined previously from cell populations across diverse biological systems. Our analyses indicate that SEGs are inherently more stable at the single-cell level and their characteristics reminiscent of HKGs, suggesting their potential role in sustaining essential functions in individual cells.<br><br>Conclusions: SEGs identified in this study have immediate utility both for understanding variation and stability of single-cell transcriptomes and for practical applications such as scRNA-seq data normalization. Our framework for calculating gene stability index, 'scSEGIndex', is incorporated into the scMerge Bioconductor R package (https://rdrr.io/bioc/scMerge/man/scSEGIndex.html) and can be used for identifying genes with stable expression in scRNA-seq datasets.
**Corresponding Author:**	Pengyi Yang<br>University of Sydney<br>Sydney, NSW AUSTRALIA
**Corresponding Author Secondary Information:**	
**Corresponding Author's Institution:**	University of Sydney
**Corresponding Author's Secondary Institution:**	
**First Author:**	Yingxin Lin
**First Author Secondary Information:**	
**Order of Authors:**	Yingxin Lin
	Shila Ghazanfar

Powered by Editorial Manager® and ProduXion Manager® from Aries Systems Corporation

	Dario Strbenac
	Andy Wang
	Ellis Patrick
	Dave M Lin
	Terence Speed
	Jean YH Yang
	Pengyi Yang
**Order of Authors Secondary Information:**	
**Response to Reviewers:**	Reviewer Comments:

Reviewer #1:
In this manuscript #GIGA-D-18-00467R1, authors applied a computational framework that they previous proposed to identify stably expressed genes (SEG) mainly from three scRNA-seq dataset (one from human and two from mouse). Authors did comparison analysis to prove that the SEGs defined by scRNA-seq are more stable than previously reported housekeeping(HSK) genes from bulk microarray or RNA-seq. Defining a credible SEGs is an interesting topic and important for normalization of scRNA-seq. Here are still some comments for this manuscript.

Major comments
1. It is obvious that the SEGs are different generated from different scRNA-seq datasets. As figure 3b showed, 830 and 916 mSEGs were identified from early mouse development data and union of mouse development and mouse atlas data respectively. It will be better to generate a more reliable SEGs by using several datasets. Or at least assess how different the SEGs from different dataset.

Response: We appreciate the suggestion to use several datasets for deriving SEGs. However, since different scRNA-seq datasets are often generated from using different protocols, platforms and quantified different numbers of genes at different depths in their original experiments, it is challenging to consolidate across multiple datasets and normalise the unwanted factors prior to identifying SEGs. More importantly, datasets used for SEG identification should contain normal cell types and profile heterogenous tissues and cell types because data only contain homogeneous cell types cannot provide the foundation for identifying genes stably expressed in different tissues/cell types, and data contain abnormal cell types such as cancers may derive genes that are abnormally stable in cancers. Therefore, it seems to us that it is preferable to limit the number of datasets and instead choose those that comprehensively profile normal tissues/cell types and developmental stages. We have noted this in the fourth paragraph in the discussion section of the revised manuscript.

To demonstrate the above point, we have now also used our framework to identify "hSEGs" from Multicellular metastatic melanoma dataset (see Table 1 of the manuscript). While this dataset contains a relatively large number of cells, it has resulted a much different stability index compared to those derived from human development dataset as shown in the scatter plot (attached in the "response letter with graphical panels"). When tested for stability using the evaluation dataset (Adult and fetal brain), these "hSEGs" derived from melanoma tissues are not better than using all genes and much less effective compare to those derived from human development dataset as can be seen in table below (attached in the "response letter with graphical panels").

2. In the section "Comparative analysis of SEGs identified in single cells and HKGs defined from bulk transcriptome", that the author did comparison between SEGs and HKGs only based on SEGs generating dataset is not enough to support the conclusion. Other evaluate datasets mentioned in table 1 should also be used to do the comparison.

Response: As we have demonstrated above, datasets that profile diverse tissue types and developmental stages are best suited for deriving SEGs. We hope this reviewer

	agrees with us that the comparative analysis of SEGs and HKGs should be performed using SEGs derived from the three SEG identification datasets. Drawing conclusions from using more restricted profiling datasets (e.g. peripheral blood) and/or datasets that profiling abnormal tissues (e.g. melanoma) may lead to incorrect conclusions.<br><br>3.It will be better to explain the selection criteria of SEG more clear, like why set the cutoff of 80 and 60.<br><br>Response: The selection of a set of SEGs is primarily for evaluation purposes and our framework provides a continuum ranking of all genes by generating a stability index. As suggested, we have now noted this in the revised manuscript (Discussion section, paragraph 4).<br><br>Minor comments<br>1.There should be randomly sampling for several times in the analysis of figure 2C, so the range of the r should be mentioned in manuscript.<br><br>Response: As suggested, we have included the standard deviation of correlation from multiple sampling in figure 2C and updated the figure legend.<br><br>2.There are some incorrect spellings. Like figure legend of figure 3B "altas" should be "atlas"; (E), at the end figure legend of figure 3D should be deleted.<br><br>Response: We thank the reviewer for identifying these typos. We have corrected them.<br><br>3.It needs to label the test approach used in figure 5D.<br><br>Response: As suggested, we have stated the test approach in the legend of Figure 5D in the revised manuscript.<br><br>4. There is some problem with the format of reference 57.<br><br>Response: We have rectified this in the revised manuscript.
**Additional Information:**	
**Question**	**Response**
Are you submitting this manuscript to a special series or article collection?	No
**Experimental design and statistics**<br><br>Full details of the experimental design and statistical methods used should be given in the Methods section, as detailed in our Minimum Standards Reporting Checklist. Information essential to interpreting the data presented should be made available in the figure legends.<br><br>Have you included all the information requested in your manuscript?	Yes
**Resources**<br><br>A description of all resources used,	Yes

including antibodies, cell lines, animals and software tools, with enough information to allow them to be uniquely identified, should be included in the Methods section. Authors are strongly encouraged to cite Research Resource Identifiers (RRIDs) for antibodies, model organisms and tools, where possible.<br><br>Have you included the information requested as detailed in our Minimum Standards Reporting Checklist?	
**Availability of data and materials**<br><br>All datasets and code on which the conclusions of the paper rely must be either included in your submission or deposited in publicly available repositories (where available and ethically appropriate), referencing such data using a unique identifier in the references and in the "Availability of Data and Materials" section of your manuscript.<br><br>Have you have met the above requirement as detailed in our Minimum Standards Reporting Checklist?	Yes

Powered by Editorial Manager® and ProduXion Manager® from Aries Systems Corporation

Manuscript

Click here to access/download;Manuscript;main_v4_SG.pdf

Click here to view linked References

OXFORD (GIGA)^n SCIENCE

GigaScience, 2017, 1–10

doi: xx.xxxx/xxxx
Manuscript in Preparation
Paper

PAPER

Evaluating stably expressed genes in single cells

Yingxin Lin[1], Shila Ghazanfar[1,8], Dario Strbenac[1], Andy Wang[1,3], Ellis Patrick[1,4], David M Lin[5], Terence Speed[6,7], Jean YH Yang[1,*] and Pengyi Yang[1,2,*]

[1]School of Mathematics and Statistics, University of Sydney, NSW 2006, Australia and [2]Computational Systems Biology Group, Children's Medical Research Institute, University of Sydney, Westmead, NSW 2145, Australia and [3]Sydney Medical School, University of Sydney, NSW 2006, Australia and [4]Westmead Institute for Medical Research, University of Sydney, Westmead, NSW 2145, Australia and [5]Department of Biomedical Sciences, Cornell University, Ithaca, NY, 14853, USA and [6]Bioinformatics Division, Walter and Eliza Hall Institute of Medical Research, 1G Royal Parade, Parkville, VIC 3052, Australia and [7]Department of Mathematics and Statistics, University of Melbourne, Melbourne, VIC 3010, Australia and [8]Cancer Research UK Cambridge Institute, University of Cambridge, Li Ka Shing Centre, Robinson Way, Cambridge CB2 0RE, United Kingdom

* pengyi.yang@sydney.edu.au; jean.yang@sydney.edu.au

Abstract

Background: Single-cell RNA-seq (scRNA-seq) profiling has revealed remarkable variation in transcription, suggesting that expression of many genes at the single-cell level are intrinsically stochastic and noisy. Yet, on the cell population level, a subset of genes traditionally referred to as housekeeping genes (HKGs) are found to be stably expressed in different cell and tissue types. It is therefore critical to question whether stably expressed genes (SEGs) can be identified on the single-cell level, and if so, how can their expression stability be assessed? We have previously proposed a computational framework for ranking expression stability of genes in single cells for scRNA-seq data normalization and integration. In this study, we perform detailed evaluation and characterization of SEGs derived from this framework.
Results: Here, we show that gene expression stability indices derived from the early human and mouse development scRNA-seq datasets and the 'Mouse Atlas' dataset are reproducible and conserved across species. We demonstrate that SEGs identified from single cells based on their stability indices are considerably more stable than HKGs defined previously from cell populations across diverse biological systems. Our analyses indicate that SEGs are inherently more stable at the single-cell level and their characteristics reminiscent of HKGs, suggesting their potential role in sustaining essential functions in individual cells.
Conclusions: SEGs identified in this study have immediate utility both for understanding variation and stability of single-cell transcriptomes and for practical applications such as scRNA-seq data normalization. Our framework for calculating gene stability index, 'scSEGIndex', is incorporated into the scMerge Bioconductor R package (https://rdrr.io/bioc/scMerge/man/scSEGIndex.html) and can be used for identifying genes with stable expression in scRNA-seq datasets.

Key words: Stably expressed genes; Single cells; scRNA-seq; Housekeeping genes; Gene expression variability

Background

A hallmark of single-cell RNA-seq (scRNA-seq) data has been the remarkable variation in gene transcription that occurs at

Compiled on: June 25, 2019.
Draft manuscript prepared by the author.

Table 1. Summary of scRNA-seq datasets utilized for stably expressed gene identification and/or evaluation in this study.

ID	Publication	Description	Organism	# cell	# class	Protocol	Purpose
E-MTAB-3929	[20]	Human development	Human	1529	5	SMART-Seq2	identify
GSE45719	[21]	Mouse development	Mouse	269	8	SMART-Seq2	identify
GSE109774	[22]	Mouse atlas	Mouse	41965	68	SMART-Seq2	identify
GSE94820	[23]	Peripheral blood mononuclear cells	Human	1140	5	SMART-Seq2	evaluate
GSE75748	[24]	PSCs and endoderm progenitors	Human	1018	7	SMARTer	evaluate
GSE72056	[25]	Multicellular metastatic melanoma	Human	4645	7	SMART-Seq2	evaluate
GSE67835	[26]	Adult and fetal brain	Human	466	8	SMARTer	evaluate
GSE60361	[27]	Cortex and hippocampus	Mouse	3005	7	SMARTer	evaluate
GSE52583	[28]	Developmental lung epithelial cells	Mouse	198	4	SMARTer	evaluate
E-MTAB-4079	[29]	Mesoderm diversification	Mouse	1205	4	SMART-Seq2	evaluate
GSE84133	[30]	Pancreas inter- and intra-cells	Mouse	822	13	InDrop	evaluate

the level of individual cells [1]. The high degree of variation has led to the appreciation that transcription of genes at the single-cell level are comparatively noisier than on the cell population level [2]. Indeed, a subset of genes are thought to be characterized by their stochastic expression [3]. Supporting this notion, genes were found to show transcriptional bursting where their expression varies drastically in individual cells [4, 5]. Furthermore, a large number of genes from scRNA-seq data exhibit bimodality or multimodality of non-zero expression values [6], suggesting that many of these genes may be expressed at different levels in the same and/or different cells. These phenomena illustrate that expression stochasticity is an intrinsic property of many genes on the single-cell level [7].

On the cell population level, however, a subset of genes traditionally referred to as housekeeping genes (HKGs) [8, 9] are found to be stably expressed in different cell types, tissue types and developmental stages [10]. The concept of HKGs is often related to the gene set required to maintain basic cellular functions and therefore is crucial to the understanding of the core transcriptome that is required to sustain life [11, 12, 13]. Early studies such as those by [14], [15], [8], and [16] were conducted to define HKGs using serial analysis of gene expression (SAGE) or microarrays. With the advent of biotechnologies, follow-up studies using more comprehensive data sources such as those by [17] and [18], and high-throughput RNA sequencing (RNA-seq) by [10] and [19], have refined the list of HKGs from populations of cells.

Taken together, the findings from bulk transcriptome data of cell populations and the stochasticity in gene expression observed in individual cells from scRNA-seq data, several fundamental questions arise including (i) can patterns of stably expressed genes be identified from single cell data? And if so, (ii) how stable are they across individual cells from different tissue types and biological systems? (iii) What properties do such genes have? And (iv) how do they compare to HKGs defined from bulk transcriptome data? In this study, we set out to answer each of these questions.

Leveraging the advances of scRNA-seq techniques [31, 32], we have previously developed a computational framework to rank genes based on various properties extracted from scRNA-seq data to characterize their expression stability in individual cells [33]. These genes were subsequently utilized for scRNA-seq data normalization and integration. To address the questions posed above, here, we applied the proposed framework on two high-resolution scRNA-seq datasets in which a wide range of cell types and developmental stages were profiled in human [20] and mouse [21], and also the 'Mouse Atlas' scRNA-seq dataset that comprehensively profiled across major mouse organs and tissue types [22]. We referred to the list of stably expressed genes derived from these datasets as "hSEG" and "mSEG" for human and mouse respectively, and collectively as "SEGs". We subsequently evaluated the stability of SEGs on a collection of independent scRNA-seq datasets generated from diverse tissues and biological systems, and different sequencing protocols. Compared to HKGs previously defined using bulk microarray [16] or RNA-seq datasets [10], SEGs identified on the single-cell level are considerably more stable in all tested biological systems, demonstrating the higher resolution enabled by scRNA-seq data for identifying genes that are truly stably expressed across individual cells, and suggesting their potential roles in maintaining essential functions in individual cells.

Our analyses highlight the previously unappreciated gene stability at the single-cell level. Our computational framework, incorporated as part of the scMerge Bioconductor R package (https://rdrr.io/bioc/scMerge/man/scSEGIndex.html), also allows further identification and refining of SEGs in other scRNA-seq datasets. This will have broad applications in normalization [34, 35] and removal of unwanted variation [36, 37, 33] in scRNA-seq as well as bulk sequencing datasets generated from various experiments.

Data Description

scRNA-seq data processing

A collection of 11 publicly available scRNA-seq datasets (Table 1) were utilized in this study. These datasets were downloaded from either NCBI GEO repository or the EMBL-EBI ArrayExpress repository. Fragments per kilobase of transcript per million (FPKM) values or counts per million (CPM) from their respective original publications were used to quantify full length gene expression for datasets generated by SMARTer or SMART-Seq2 protocols. UMI-filtered counts were used to quantify gene expression for the InDrop dataset. Data were transformed by $\log_2(x + 1)$ where x is the original quantification (e.g. CPM). All datasets have undergone cell-type identification using biological knowledge assisted by various clustering algorithms from their respective original publications which we retain for evaluation purposes. For each dataset, genes with more than 80% missing values (zeros) were removed, with the remaining genes considered as expressed in that dataset. These filtered datasets were used for all subsequent analyses.

Analyses

A computational framework for measuring gene expression stability in single cells

We recently proposed a mixture modeling computational framework for characterizing gene expression stability using scRNA-seq data [33]. The original framework uses a

Gamma component to fit the lower end of the distribution given the non-negative values of gene expression [38] and a Gaussian component to fit the higher end for capturing variability in cells that express a given gene (Figure 1A). To test if a Gamma component would be better suited for fitting the higher end of the distribution, here we compared the choices of Gamma-Gaussian and Gamma-Gamma mixture models using Bayesian information criterion (BIC). We found that the Gamma-Gaussian mixture fits consistently better than Gamma-Gamma mixture across multiple datasets (Figure 1B).

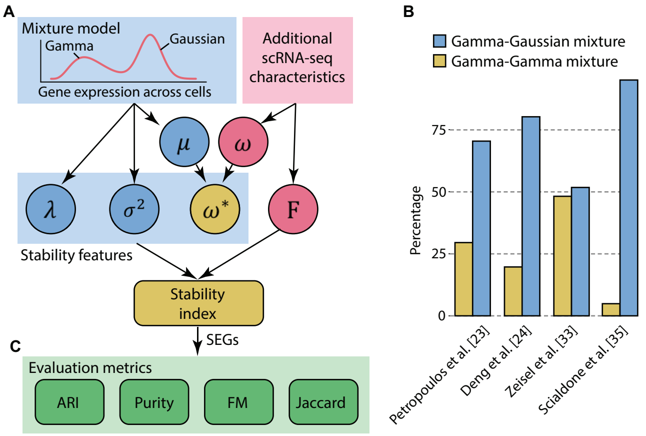

Figure 1. Schematic illustration of the computational framework for deriving gene stability index on the single-cell level. (A) Stability features extracted directly from the mixture model are colored in blue. Those extracted from additional scRNA-seq data characteristics are in red. The overall stability index is derived from the combination of all stability features. (B) Comparison of Gamma-Gaussian and Gamma-Gamma mixture models on four scRNA-seq datasets. *y*-axis represents the percentage of times a given model is selected by Bayesian information criterion (BIC). (C) Evaluation metrics used for evaluating gene expression stability in scRNA-seq datasets.

Using the Gamma-Gaussian mixture model, we extract a set of stability features including λ, σ^2, ω^*, and the F-statistics, and derive a stability index for each gene on the single-cell level. The μ and σ^2 denote the mean and variance of the Gaussian component fitted to a gene x across individual cells. The joint density function $f(.)$ is defined as follows:

$$f(.) = \lambda \frac{\beta^{\alpha}}{\Gamma(\alpha)} x^{\alpha-1} e^{-\beta x} + (1-\lambda) \frac{1}{\sigma\sqrt{2\pi}} e^{-\frac{(x-\mu)^2}{2\sigma^2}}$$

where $0 \leq \lambda \leq 1$ is the mixing proportion indicating the proportion of cells in the Gamma component in the fitted model. Genes whose expression profiles are with low mixing proportion (λ) and small variance (σ^2) are unimodal and relatively invariant across cells and therefore more likely to be stably expressed.

The ω denotes the percentage of zeros of a gene across cells. The measured expression level for a given gene and cell may be zero due to technical dropout, stochastic expression, or no transcription occurring at all for that gene [39]. Thus, SEG would have relatively small ω (i.e. low proportion of zeros), since they are expected to be expressed in all cells. However, lowly expressed genes tend to have a higher proportion of zeros than highly expressed genes simply due to technical dropouts [40]. We therefore regularized the proportion of zeros (ω) of each gene based on its average expression level μ in the Gaussian component by $\omega^* = \omega \cdot \text{minmax}(\mu)$, where minmax(.) scales the ω^* to the range of 0 to 1. This regularization accounts for the dropout bias towards genes with lower expression.

When pre-defined cell type annotation is available for a given dataset, the F-statistics can be utilized as another stability feature to select for genes in which we observe the same average gene expression across different pre-defined cell types. Together, genes with small λ, σ^2, ω^* and F-statistic are unimodal, expressed with low variance, with relatively low percentage of zeros, and expressed similarly across all cell types, respectively, and are more likely to be stably expressed.

The expression stability index is defined for each gene by combining these four stability features. Specifically, genes are ranked first in increasing order with respect to λ, σ^2, ω^* and F-statistics; and the ranks from each stability features are rescaled to range from 0 to 1. The stability index for each gene is defined as the average of its scaled rankings across all four stability features. Thus, genes are ranked in terms of their degree of evidence towards expression stability in individual cells and can be selected by adjusting the stability index threshold. The subsequent evaluation can be conducted to assess the stability and generalization property of selected SEGs in other biological systems using various evaluation metrics (Figure 1C).

Genes are reproducibly ranked by their expression stability in single cells

To investigate if some genes are inherently more stable in expression on the single-cell level, we utilized three high-resolution scRNA-seq datasets (e.g. human development, mouse development, and the mouse atlas) to quantify genes that are expressed at steady levels across different cell types, tissues, and developmental stages of human and mouse, respectively (Table 1; datasets labeled as 'identify'). These datasets provide a starting point for identifying SEGs that can then be used for evaluation on various cell/tissue types and biological systems (Table 1; datasets labeled as 'evaluate').

We first looked at the proportion of zeros per gene across all profiled cells in the early human and mouse development scRNA-seq datasets respectively. We found that a large percentage of genes have more than 50% zero quantification across cells in both datasets (Figure 2A), suggesting most of the genes are transiently expressed in different cell/tissue types and during different developmental stages in both human and mouse. We observed that the distributions of stability features across the three scRNA-seq datasets are different (Figure 2B). Nevertheless, our rank-based approach scales ranks of genes with respect to each stability feature and derives highly comparable stability index distributions from each dataset (Figure 2B, bottom right panel).

We next investigated the reproducibility of the stability index by randomly sampling 80% of all cells from the human and mouse development datasets and re-calculating the stability index for each sub-sample. We found the stability index to be highly reproducible (Figure 2C) within a dataset with average Pearson correlation coefficients of 0.98 and 0.97. In comparison, the correlation of stability indices from the mouse development and mouse atlas datasets are much more moderate (Figure 2D), suggesting room for further improvement when more comprehensive and deeper scRNA-seq datasets become available. We also observed that the stability indices derived for human and mouse are significantly correlated (Figure 2D).

Comparative analysis of SEGs identified in single cells and HKGs defined from bulk transcriptome

To understand the relationships of genes with stable expression in single cells with HKGs defined previously with bulk microarray [16] and RNA-seq [10], we derived a list of SEGs for human and mouse respectively by computing the rank percentiles of stability index as well as the four stability features.

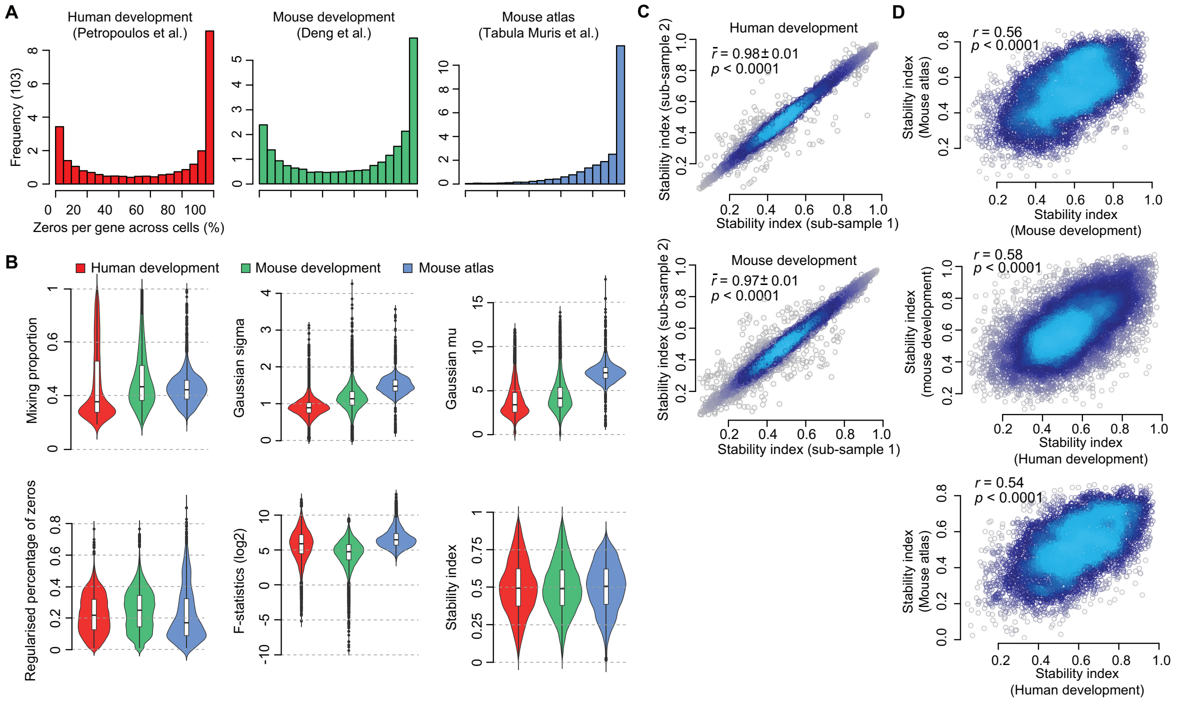

Figure 2. Characterizing gene stability features in single cells for human and mouse. (A) Percentage of zeros per gene across individual cells. (B) Fitted values of mixing proportion (λ), and variance (σ^2) and mean (μ) in the Gaussian component (top panels) of the mixture model for each gene. Regularized percentage of zeros, *F*-statistics computed from pre-defined cell class (bottom left panel) and stability index derived for each gene (bottom right panel), respectively. (C) Scatter plot of stability index calculated from two random sub-sampling of cells from human and mouse development datasets. Mean Pearson's correlation coefficient and standard deviation ($\bar{r} \pm sd$) were calculated from pairwise comparison of 10 repeated random sub-sampling on each dataset (D) Scatter plot and correlation of stability indices calculated from each of three datasets. *p*-values denote *t*-distribution test on Pearson's correlation coefficient.

Genes with a stability index rank percentile above 80 as well as a reversed rank percentile above 60 for each of the four stability features were included in the SEG list. For mouse, we took the union of the SEGs identified from the mouse development and mouse atlas datasets. This resulted in lists of 1,076 human (hSEG) and 916 mouse (mSEG) genes, respectively (Figure 3A and B). In comparison to the HKGs defined previously using bulk transcriptomes, we found that hSEG identified on the single-cell level have significantly smaller expression variances across individual cells (Figure 3A).

Comparing with previously defined HKGs (Figure 3C), there were 676 common genes between our hSEG list and those defined by microarray or bulk RNA-seq. This accounts for 62% of hSEGs, a statistically significant overlap (permutation $p < 2e-5$), highlighting a high level of commonality but also uniqueness of SEGs. For the human and mouse SEG lists derived from scRNA-seq datasets, there were 272 common genes (Figure 3D) which accounts for a significant portion of genes in both lists (25% w.r.t. hSEG and 30% w.r.t. mSEG; permutation $p < 2e-5$), in agreement with the correlation analysis (Figure 2D), suggesting their conservation between human and mouse.

To investigate the difference between SEGs and HKGs defined by bulk transcriptomes, we inspected a few individual genes that were defined as SEGs using scRNA-seq data but not HKGs by bulk microarray or RNA-seq, and *vice versa*. We discovered that many ribosomal proteins (such as *RPL26* and *RPL36*) that were included in the SEG list but not in the HKG lists (Figure 3E) showed strong unimodal expression patterns across all cells. In contrast, genes such as *HINT1* (Histidine triad nucleotide-binding protein 1) and *AGPAT1* (1-Acylglycerol-3-Phosphate O-Acyltransferase), both of which have been reported to be differentially expressed in brain tissue [41] or malignant oesophageal tissues [42] compared to normal samples, were included in both microarray and RNA-seq defined HKG lists, but not in SEG list due to their bimodal expression patterns across individual cells.

Finally, we examined the expression patterns of *GAPDH* and *ACTB* (Figure 3F), genes which are commonly treated as canonical HKGs for data normalization, and observed clear bimodality in both the human and mouse data. In agreement with previous studies [10, 17, 34, 43], these data argue against their usage as "housekeeping genes" for sample normalization.

SEGs exhibit strong expression stability in single cells across different tissues and biological systems

We hypothesized that if the expression levels of the SEGs are relatively stable, they should show relatively small expression differences across the different cell types from various biological systems. We first investigated principal component analysis (PCA) plots generated from early human and mouse development data using all genes (all expressed mRNA), or subsets of genes defined for human (i.e. HKG microarray, HKG RNA-seq, and hSEG) (Figure 4A) and mouse (i.e. mSEG) (Figure 4B). We found that for human data there is clear separation of developmental stages in the first two principal components when PCA plots were created by using either all genes, and HKGs defined from microarray or RNA-seq, suggesting genes that were expressed differentially in different developmental stages were driving the separation. In contrast, the PCA plot generated from using hSEG show much less separation with respect to the developmental stages, suggesting they are generally expressed at a similar level across individual cells irrespective to cell differentiation and change of developmental stages. Similar results were observed from mouse development data (Figure 4B) where the PCA plot generated from mSEG show less separation of cell type and development stage compared to PCA plot generated from using all genes.

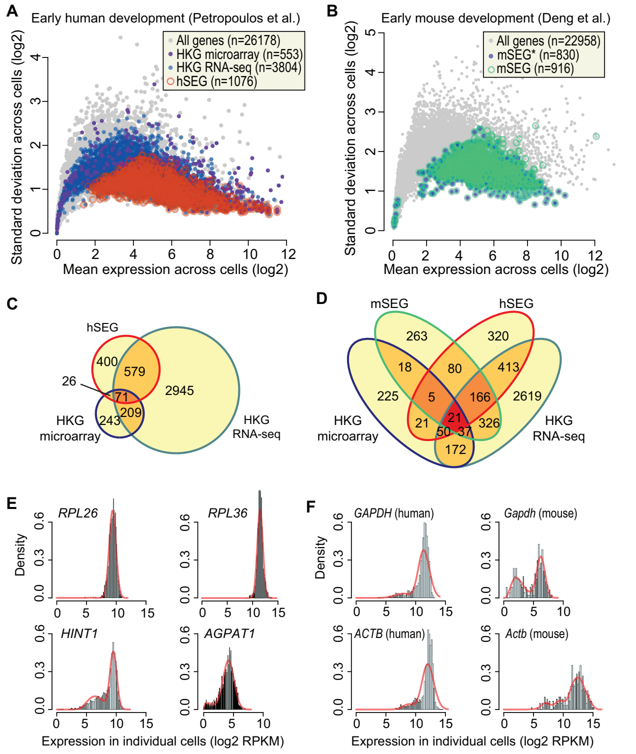

Figure 3. Comparison of SEGs identified on individual cell level using scRNA-seq with HKGs defined on cell population level using bulk transcriptome data. (A) Scatter plot showing mean expression (x-axis) and variance (y-axis) of each gene (gray circles) across profiled single cells. Open red circles represent SEGs identified from early human development data (hSEG) in this study whereas dark and light blue solid circles represent HKGs defined previously using bulk microarray [16] and RNA-seq data [10]. (B) Same as (A) but for SEGs identified from early mouse development data (mSEG*; light blue points) and the union of these identified from both mouse development and mouse atlas datasets (mSEG; green circles). (C) Venn diagrams showing overlaps of hSEGs and HKGs defined using bulk microarray and RNA-seq. (D) Overlap of all human and mouse gene lists. (E) Expression patterns of example genes that are defined as SEGs using scRNA-seq data but not as HKGs using bulk microarray or RNA-seq data (*RPL*26 and *RPL*36) and vice versa (*HINT* and *AGPAT*1) across individual cells. (F) Expression patterns for *GAPDH* and *ACTB* in human and mouse (*Gapdh* and *Actb*) across individual cells.

To quantify the above visual observations in human and mouse developmental datasets, we utilized k-means clustering to partition cells into five and eight clusters respectively, using all genes (all expressed mRNA) or subsets of genes defined in each list (i.e. hSEG, mSEG, HKG microarray and HKG RNA-seq) with the hypothesis that clusters arising from using SEGs and HKGs will exhibit lower concordance with pre-defined cell type- and tissue-specific labels (Figure 4C), thereby demonstrating consistent levels of expression across different cell and tissue types. To account for the size difference of the gene lists, we also created subsets of HKGs identified from RNA-seq data (sub HKG RNA-seq) to match the sizes of hSEGs and mSEGs, respectively; and subsets of hSEGs (sub hSEG) and mSEGs (sub mSEG) to match the size of HKGs identified from microarray data.

We found that k-means clustering outputs using SEGs derived from scRNA-seq data showed the lowest concordance to their pre-defined cell class labels (i.e. embryonic day of development or cell types) as quantified by the adjusted rand index (ARI), Purity, Fowlkes-Mallows index (FM), and Jaccard index (Figure 4D). The reduction of either list to match the other had relatively minor effect on the clustering results. These results demonstrate that SEGs are stably expressed across cells and developmental stages in the two scRNA-seq datasets.

To test whether SEGs derived above are stably expressed in other cell and tissue types, we evaluated these SEGs and their subsets that matched the size of HKGs defined from microarray data on eight datasets (Table 1) which are independent of the scRNA-seq datasets used for identifying SEGs. These additional datasets represent drastically different tissues and biological systems in both human and mouse, as well as different sequencing protocols and a wide range in the number of cells sequenced.

Similar to the above section, we quantified the clustering concordance with respect to each of their pre-defined cell class labels using each of the four concordance metrics (ARI, Purity, FM, and Jaccard) (Table 2). We found that on average, clustering using SEGs (and their subsets) gave the lowest concordance to the pre-defined cell type- and tissue-specific class labels in all tested datasets compared to clustering using all expressed genes or HKGs defined using bulk microarray and RNA-seq datasets. These results suggest that SEGs defined in early human and mouse development also display strong expression stability in various cell/tissue types and biological systems, and they are considerably more stable than HKGs defined using bulk transcriptome data on the single-cell level.

Gene stability index derived from single cells correlates with gene sequence and structural characteristics

To further characterize gene expression stability in single cells, we correlated the stability index and each stability feature extracted from scRNA-seq data with various gene structural and conservation features calculated from various data sources. We found that the stability index correlated positively with the number of exons in a gene, gene expression, and gene conservation, and negatively with GC-content in the gene body in both human and mouse (Figure 5A), many of which are characteristics of HKGs reported in previous studies. Consistent with this, we found SEGs are more evolutionarily conserved [44] with higher phyloP scores. SEGs also possess more exons, in agreement with previous finding on HKGs [45], despite mouse genes on average having fewer exons than human genes. Both human and mouse SEGs appeared to have a slightly lower GC-content but, similar to previous observation on HKGs, the relation was relatively weak [46] (Figure 5B).

Perhaps unsurprisingly, SEGs identified in this study possess similar characteristics to those observed in HKGs, indicating that they are serving essential cellular functions akin to HKGs. Supporting this, we found that multiple top-enriched Gene Ontology and Reactome terms that describe essential cellular functions are shared by common SEGs (genes overlap between hSEG and mSEG) and common HKGs (genes overlap between HKG microarray and HKG RNA-seq) (Figure 5C) (see Methods for details). Nevertheless, common SEGs are far more enriched for most GO and Reactome terms than common HKGs defined from bulk transcriptome and also show significantly higher conservation in both human and mouse (Figure 5D). These results indicate the higher resolution enabled by scRNA-seq data for identifying genes that are truly stably expressed across individual cells.

Discussion

Since the emergence of high-throughput transcriptome profiling, the search for stably expressed genes (SEGs) has been a central quest in modern biology. Such genes are often thought to be essential for basic cellular functions given their relatively constant expression and activity despite changes in cell status and types. The hypothesis that such genes may serve the same

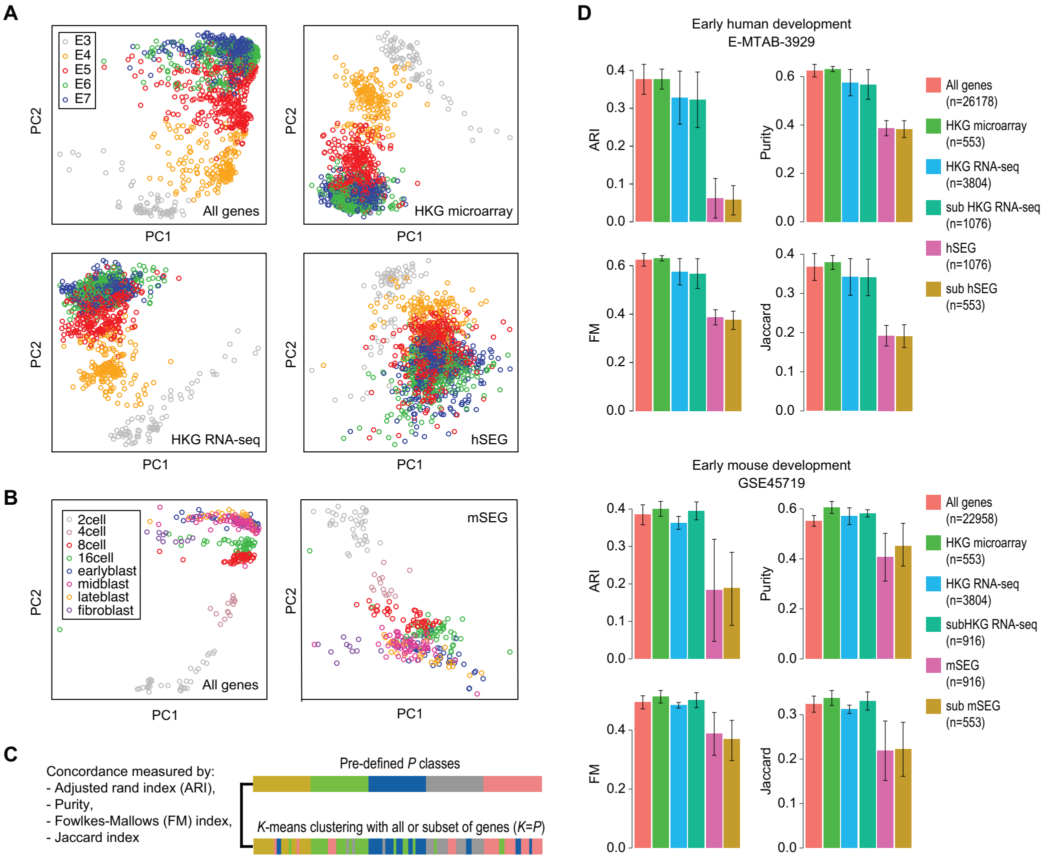

Figure 4. Stability of SEGs and HKGs in human and mouse development scRNA-seq datasets. (A) PCA plots generated from human development data using all expressed genes, HKGs, or hSEGs. Cells are colored by their pre-defined developmental stages. (B) PCA plots generated from mouse development data using all expressed genes or mSEGs. Cells are colored by their pre-defined types and developmental stages. (C) Schematic showing the quantification of concordance of *k*-means clustering with pre-defined cell classes using a panel of metrics. (D) Barplots of concordance between *k*-means clustering and pre-defined cell class labels, using all expressed genes, HKGs identified from microarray and RNA-seq data, SEGs identified from this study for human (hSEGs) and mouse (mSEGs), and size matched subset of HKGs to SEGs and vice versa.

Table 2. Stability evaluation results on independent scRNA-seq datasets that profile various cell types and biological systems. All indices are within the range of [0, 1] and are multiplied by 100.

	Peripheral blood mononuclear cells (human); [23]					PSCs and endoderm progenitors (human); [24]				
	All genes	HKG		SEG		All genes	HKG		SEG	
		Array	RNA-seq	n=1076	n=553		Array	RNA-seq	n=1076	n=553
ARI	55±8	42±3	38±4	29±6	21±3	69±5	58±5	55±6	41±3	40±3
Purity	69±7	62±2	59±1	52±5	48±5	80±4	74±3	71±5	59±3	61±4
FM	67±5	56±1	52±3	45±4	40±2	75±4	66±4	63±5	51±2	50±3
Jaccard	49±6	39±1	35±2	29±4	25±2	60±5	48±4	46±6	34±2	33±2
	Multicellular metastatic melanoma (human); [25]					Adult and fetal brain (human); [26]				
	All genes	HKG		SEG		All genes	HKG		SEG	
		Array	RNA-seq	n=1076	n=553		Array	RNA-seq	n=1076	n=553
ARI	31±5	18±2	18±1	15±1	15±1	53±7	50±3	39±4	36±3	34±3
Purity	80±5	73±1	74±1	71±1	70±1	82±3	76±4	74±3	68±2	65±1
FM	51±3	39±2	40±1	37±1	36±1	62±6	59±2	50±3	47±3	46±3
Jaccard	32±2	22±2	24±1	21±1	20±0	44±6	41±2	33±3	30±3	29±2
	Cortex and hippocampus (mouse); [27]					Developmental lung epithelial cells (mouse); [28]				
	All genes	HKG		SEG		All genes	HKG		SEG	
		Array	RNA-seq	n=916	n=553		Array	RNA-seq	n=916	n=553
ARI	45±8	36±5	31±3	28±3	26±2	61±6	55±4	48±2	46±0	43±5
Purity	72±3	66±1	63±1	59±1	58±2	83±4	80±2	76±1	75±0	73±3
FM	55±6	49±4	44±3	42±2	40±2	72±4	68±3	62±2	61±0	59±4
Jaccard	38±6	32±4	28±2	26±2	25±2	56±5	51±3	45±2	44±0	42±4
	Mesoderm diversification (mouse); [29]					Pancreas inter- and intra-cells (mouse); [30]				
	All genes	HKG		SEG		All genes	HKG		SEG	
		Array	RNA-seq	n=916	n=553		Array	RNA-seq	n=916	n=553
ARI	54±2	43±8	49±3	31±7	10±7	37±4	22±3	23±3	19±2	17±3
Purity	66±1	62±6	65±1	59±7	48±7	89±3	78±3	76±2	74±2	71±2
FM	68±1	63±8	67±1	59±7	53±5	52±4	38±3	39±3	35±2	32±3
Jaccard	52±1	46±7	50±1	40±8	32±7	30±3	20±3	21±3	17±2	16±2

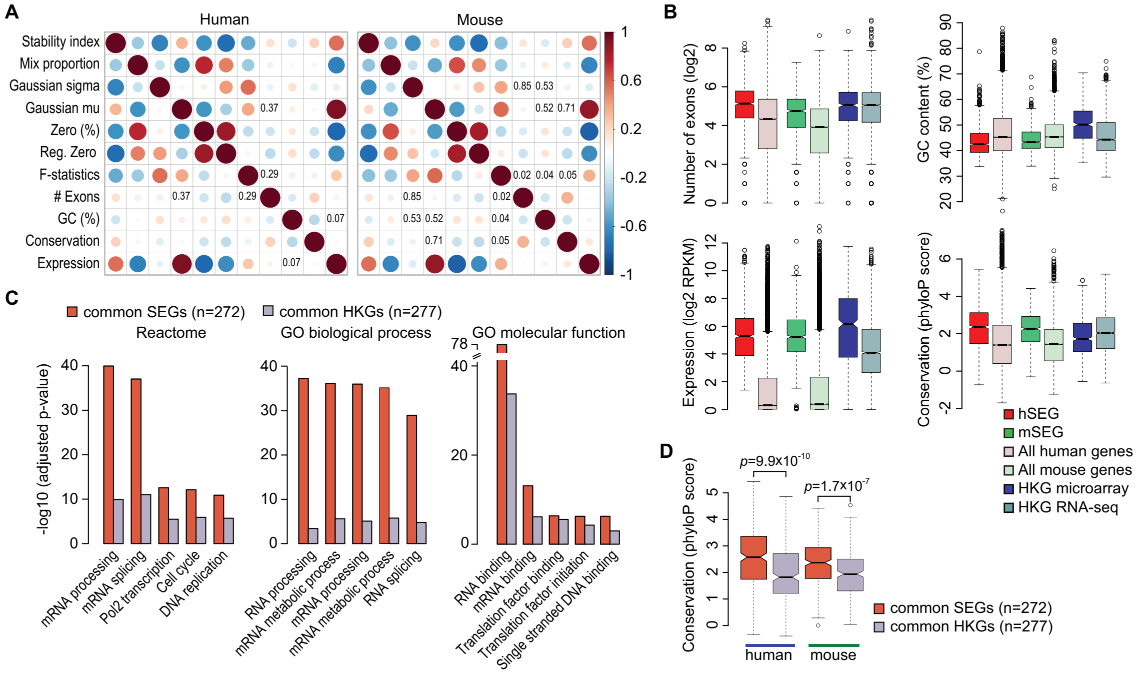

Figure 5. Characterization of stability index with sequence and gene characteristics. (A) Pearson correlation analyses of human and mouse gene stability features with respect to genomic structural and evolutionary gene features. p-values greater than 0.001 are displayed. (B) Boxplots of various gene characteristics for SEGs, HKGs and all expressed genes. (C) Over-representation analyses of SEGs that are common between hSEG and mSEG (common SEGs); and HKGs that are common between HKG microarray and HKG RNA-seq (common HKGs), using Gene Ontology (GO) and Reactome databases. (D) Comparison of conservation for common SEGs and common HKGs in human and mouse genomes. p-values were calculated from a two-sided Wilcox rank sum test.

housekeeping functions across various cell and tissue types has also led to their definition as "housekeeping genes" (HKGs). While the existence of true HKGs whose expression are universally constant across all cells and systems is a subject of debate [42, 47], their practical usage as control genes for experimental data normalization is well appreciated.

Recent advances in single-cell transcriptome profiling using scRNA-seq have highlighted the phenomenal amount of gene expression stochasticity and heterogeneity in single cells. Compared to bulk transcriptome data that aggregate millions of cells to obtain a single gene expression measure, scRNA-seq data allows the expression dynamics of each gene within individual cells to be monitored, and therefore enables the identification of genes that are truly expressed at a steady level in individual cells across tissues and developmental stages. By modeling from large-scale scRNA-seq datasets, we quantified the relative expression stability of genes on the single-cell levels. We showed that the SEGs derived based on their stability indices are considerably more stable in not only the scRNA-seq datasets from which they are identified but also independent scRNA-seq datasets that profiles various cell types and biological systems.

Our analysis demonstrated that despite the high variability in single-cell gene expression, a subset of genes is inherently more stable in expression than other genes within individual cells. Their sequence and gene structural properties are strongly reminiscent of HKGs defined from bulk transcriptome, suggesting their essential roles in maintaining basic cellular functions on the individual cell level.

While a heuristic cutoff was used to select a set of SEGs, we note that the main purpose is primarily for evaluation and comparison with HKGs. The proposed framework provides a continuous stability index for each gene and therefore allows the selection of a desired number of genes based on the stability index according to specific applications. Moreover, the proposed framework can be applied in a data dependent manner to rank genes based on their expression stability in a given scRNA-seq dataset. This relaxes the rigid binary definition of HKGs and enables a more practical definition of stable expression in different experimental contexts. Hence, the proposed method is particularly useful for defining stable or "control" genes in various scRNA-seq experiments, which is often a key step in normalizing such data [48, 49]. Indeed, the utility of SEGs on scRNA-seq data normalization has already been demonstrated by our recent study on integrating multiple scRNA-seq datasets [33]. Nevertheless, the choice of dataset for deriving SEGs is important. Datasets used for SEG identification should contain normal cell types and profile heterogeneous tissues and cell types because data only containing homogeneous cell types cannot provide the foundation for identifying genes stably expressed in different tissues and cell types, and data contain abnormal cell types such as cancers may derive genes that are abnormally stable in cancers.

As mentioned above, generalizability of SEGs is dependent on the diversity of cell types profiled in a scRNA-seq experiment. Various cell atlas profiling initiatives such as the Human Cell Atlas (https://www.humancellatlas.org) is currently under way to comprehensively characterize the transcriptome of every human cell. Information from such resources in conjunction with our computational framework will provide an even more precise assessment of gene expression stability in single cells that will enrich subsequent avenues of research including characterizing heterogeneity and stability of single-cell transcriptomes and their use for technical data normalization and standardization.

Taken together, this comprehensive evaluation study demonstrates the utility of measuring gene expression stability at the single-cell level and marks a shift in paradigm for selecting genes that are stably expressed in single cells for practical applications.

Methods

Evaluating the stability of gene lists

To assess the expression stability of each gene list in various cell types and biological systems, the k-means algorithm was utilized to cluster each scRNA-seq data to its pre-defined number of clusters and an array of evaluation metrics were applied to compute the concordance with respect to the pre-defined ("gold standard") class labels. Evaluation metrics include the adjusted Rand index (ARI), Purity, the Fowlkes-Mallows index (FM) and the Jaccard index.

Let $U = \{u_1, u_2, \ldots, u_P\}$ denote the true partition across P classes and $V = \{v_1, v_2, \ldots, v_K\}$ denote the partition produced from k-means clustering ($K = P$). Let a be the number of pairs of cells correctly partitioned into the same class by the clustering method; b be the number of pairs of cells partitioned into the same cluster but in fact belong to different classes; c be the number of pairs of cells partitioned into different clusters but belongs to the same class; and d be the number of pairs of cells correctly partitioned into different clusters. Then the Adjusted Rand Index [50], the Jaccard index [51], and the Fowlkes-Mallows index [52] can be defined as

$$\text{ARI} = \frac{2(ad - bc)}{(a + b)(b + d) + (a + c)(c + d)};$$

$$\text{Jaccard} = a/(a + b + c);$$

$$\text{FM} = \sqrt{\left(a/(a + b)\right)\left(a/(a + c)\right)};$$

and the Purity [53] can be calculated as

$$\text{Purity} = \frac{1}{N}\sum_{i}\max_{j}|u_i \cap v_j|$$

where N is the total number of cells, i and j are the indices of clusters from clustering output u_i and pre-defined class label v_j.

For each dataset, we calculated and compared the above four metrics using (i) all expressed genes, (ii) HKGs defined using microarray data [16], (iii) HKGs defined using bulk RNA-seq data [10], and (iv) SEGs identified in this study. In order to account for potential effects of gene list length, we also generated random subsets with the same number of genes in our SEG lists first by randomly sampling from all expressed genes in the dataset, and second by randomly sampling from the HKG list defined by bulk RNA-seq. Since the k-means clustering algorithm is not deterministic and the random sampling process introduces variability, the above procedure was repeated 10 times to account for such variability.

Gene properties

To characterize SEGs identified in early human and mouse development datasets, we extracted gene sequence and structural features including the number of exons and percentage GC content in the gene body for human and mouse, respectively, using biomaRt [54]. Additionally, to characterize gene evolutionary conservation, phyloP scores were downloaded from the UCSC Genome Browser for mouse (mm10) and human (hg38) genomes. Exonic bases of each gene were determined based on GENCODE Genes for human (release 26) and mouse (release 14). The set of conservation scores for each gene was averaged for each gene. We assessed the concordance of gene expression stability index and each stability feature derived from single cells with structural features, conservation scores, and their expression across all genes for human and mouse using Pearson correlation coefficients. We also compared these features for SEGs and previously defined HKGs against all expressed genes in human and mouse, respectively.

Gene ontology enrichment analysis

To perform gene ontology enrichment analysis, we first defined SEGs that are shared between hSEG and mSEG as "common SEGs" and HKGs that are shared between HKG microarray and HKG RNA-seq as "common HKGs". The similar numbers of common SEGs (256) and common HKGs (277) allowed us to avoid any potential gene-set size bias in the enrichment analysis.

Over-representation of common SEGs or common HKGs was evaluated by comparing each set of genes against ontologies defined in Gene Ontology database [55] and those defined in Reactome database [56]. Fisher's exact test was used to assess statistical significance. Top-enriched ontologies from either common SEGs or common HKGs were combined for interpretation.

Availability of supporting data and materials

Declarations

List of abbreviations

scRNA-seq: Single-cell RNA-seq; HKGs: housekeeping genes; SEGs: stably expressed genes; SAGE: serial analysis of gene expression; hSEG: stably expressed genes derived from early human developmental dataset; mSEG: stably expressed genes derived from early human developmental dataset; HKG microarray: housekeeping genes defined using bulk microarray; HKG RNA-seq: housekeeping genes defined using bulk RNA-seq; PCA: principal component analysis; ARI: adjusted rand index; FM: Fowlkes-Mallows index.

Consent for publication

Not applicable

Competing Interests

The author(s) declare that they have no competing interests.

Funding

This work is supported by Australian Research Council (ARC)/Discovery Early Career Researcher Award (DE170100759) to P.Y., National Health and Medical Research Council (NHMRC)/Career Development Fellowship (1105271) to J.Y.H.Y., ARC/Discovery Project (DP170100654) grant to P.Y. and J.Y.H.Y., and NHMRC/Program Grant (1054618) to T.P.S.

Author's Contributions

PY conceived the study with input from JYHY. All authors contributed to the design, analytics, interpretation and the direction of the study. YL and PY led the analytics and AYW led the curation of the datasets. All authors wrote, reviewed, edited, and approved the final version of the manuscript.

Acknowledgments

The authors thank their colleagues at the School of Mathematics and Statistics, The University of Sydney, and Prof. Ze-Guang Han and Dr. Xianbin Su at Shanghai Jiao Tong University for informative discussion and valuable feedback.

References

1. Martinez-Jimenez CP, Eling N, Chen HC, Vallejos CA, Kolodziejczyk AA, Connor F, et al. Aging increases cell-to-cell transcriptional variability upon immune stimulation. Science 2017;355(6332):1433–1436.
2. Marinov GK, Williams BA, McCue K, Schroth GP, Gertz J, Myers RM, et al. From single-cell to cell-pool transcriptomes: stochasticity in gene expression and RNA splicing. Genome Research 2014;24(3):496–510.
3. Kolodziejczyk AA, Kim JK, Svensson V, Marioni JC, Teichmann SA. The technology and biology of single-cell RNA sequencing. Molecular Cell 2015;58(4):610–620.
4. Suter DM, Molina N, Gatfield D, Schneider K, Schibler U, Naef F. Mammalian genes are transcribed with widely different bursting kinetics. Science 2011;332(6028):472–474.
5. Fukaya T, Lim B, Levine M. Enhancer control of transcriptional bursting. Cell 2016;166(2):358–368.
6. Shalek AK, Satija R, Adiconis X, Gertner RS, Gaublomme JT, Raychowdhury R, et al. Single-cell transcriptomics reveals bimodality in expression and splicing in immune cells. Nature 2013;498(7453):236.
7. Kærn M, Elston TC, Blake WJ, Collins JJ. Stochasticity in gene expression: from theories to phenotypes. Nature Reviews Genetics 2005;6(6):451.
8. Hsiao LL, Dangond F, Yoshida T, Hong R, Jensen RV, Misra J, et al. A compendium of gene expression in normal human tissues reveals tissue-selective genes and distinct expression patterns of housekeeping genes. Physiological Genomics 2001;.
9. Butte AJ, Dzau VJ, Glueck SB. Further defining housekeeping, or "maintenance," genes Focus on "A compendium of gene expression in normal human tissues". Physiological Genomics 2001;7(2):95–96.
10. Eisenberg E, Levanon EY. Human housekeeping genes, revisited. Trends in Genetics 2013;29(10):569–574.
11. Koonin EV. Comparative genomics, minimal gene-sets and the last universal common ancestor. Nature Reviews Microbiology 2003;1(2):127.
12. Gil R, Silva FJ, Peretó J, Moya A. Determination of the core of a minimal bacterial gene set. Microbiology and Molecular Biology Reviews 2004;68(3):518–537.
13. Glass JI, Assad-Garcia N, Alperovich N, Yooseph S, Lewis MR, Maruf M, et al. Essential genes of a minimal bacterium. Proceedings of the National Academy of Sciences 2006;103(2):425–430.
14. Velculescu VE, Madden SL, Zhang L, Lash AE, Yu J, Rago C, et al. Analysis of human transcriptomes. Nature Genetics 1999;23(4):387.
15. Warrington J, Nair A, Mahadevappa M, Tsyganskaya M. Comparison of human adult and fetal expression and identification of 535 housekeeping/maintenance genes. Physiological Genomics 2000;2(3):143–147.
16. Eisenberg E, Levanon EY. Human housekeeping genes are compact. Trends in Genetics 2003;19(7):362–365.
17. De Jonge HJ, Fehrmann RS, de Bont ES, Hofstra RM, Gerbens F, Kamps WA, et al. Evidence based selection of housekeeping genes. PloS One 2007;2(9):e898.
18. Zhu J, He F, Song S, Wang J, Yu J. How many human genes can be defined as housekeeping with current expression data? BMC Genomics 2008;9(1):172.
19. Ramsköld D, Wang ET, Burge CB, Sandberg R. An abundance of ubiquitously expressed genes revealed by tissue transcriptome sequence data. PLoS Computational Biology 2009;5(12):e1000598.
20. Petropoulos S, Edsgärd D, Reinius B, Deng Q, Panula SP, Codeluppi S, et al. Single-cell RNA-seq reveals lineage and X chromosome dynamics in human preimplantation embryos. Cell 2016;165(4):1012–1026.
21. Deng Q, Ramsköld D, Reinius B, Sandberg R. Single-cell RNA-seq reveals dynamic, random monoallelic gene expression in mammalian cells. Science 2014;343(6167):193–196.
22. Consortium TM, et al. Single-cell transcriptomics of 20 mouse organs creates a Tabula Muris. Nature 2018;562:367–372.
23. Villani AC, Satija R, Reynolds G, Sarkizova S, Shekhar K, Fletcher J, et al. Single-cell RNA-seq reveals new types of human blood dendritic cells, monocytes, and progenitors. Science 2017;356(6335):eaah4573.
24. Chu LF, Leng N, Zhang J, Hou Z, Mamott D, Vereide DT, et al. Single-cell RNA-seq reveals novel regulators of human embryonic stem cell differentiation to definitive endoderm. Genome Biology 2016;17(1):173.
25. Tirosh I, Izar B, Prakadan SM, Wadsworth MH, Treacy D, Trombetta JJ, et al. Dissecting the multicellular ecosystem of metastatic melanoma by single-cell RNA-seq. Science 2016;352(6282):189–196.
26. Darmanis S, Sloan SA, Zhang Y, Enge M, Caneda C, Shuer LM, et al. A survey of human brain transcriptome diversity at the single cell level. Proceedings of the National Academy of Sciences 2015;112(23):7285–7290.
27. Zeisel A, Muñoz-Manchado AB, Codeluppi S, Lönnerberg P, La Manno G, Juréus A, et al. Cell types in the mouse cortex and hippocampus revealed by single-cell RNA-seq. Science 2015;347(6226):1138–1142.
28. Treutlein B, Brownfield DG, Wu AR, Neff NF, Mantalas GL, Espinoza FH, et al. Reconstructing lineage hierarchies of the distal lung epithelium using single-cell RNA-seq. Nature 2014;509(7500):371.
29. Scialdone A, Tanaka Y, Jawaid W, Moignard V, Wilson NK, Macaulay IC, et al. Resolving early mesoderm diversification through single-cell expression profiling. Nature 2016;535(7611):289.
30. Baron M, Veres A, Wolock SL, Faust AL, Gaujoux R, Vetere A, et al. A single-cell transcriptomic map of the human and mouse pancreas reveals inter-and intra-cell population structure. Cell Systems 2016;3(4):346–360.
31. Tang F, Barbacioru C, Wang Y, Nordman E, Lee C, Xu N, et al. mRNA-Seq whole-transcriptome analysis of a single cell. Nature Methods 2009;6(5):377.
32. Jaitin DA, Kenigsberg E, Keren-Shaul H, Elefant N, Paul F, Zaretsky I, et al. Massively parallel single-cell RNA-seq for marker-free decomposition of tissues into cell types. Science 2014;343(6172):776–779.
33. Lin Y, Ghazanfar S, Wang KYX, Gagnon-Bartsch JA, Lo KK, Su X, et al. scMerge leverages factor analysis, stable expression, and pseudoreplication to merge multiple single-cell RNA-seq datasets. Proceedings of the National Academy of Sciences 2019;116(20):9775–9784. https://www.pnas.org/content/116/20/9775.
34. Thellin O, Zorzi W, Lakaye B, De Borman B, Coumans B, Hennen G, et al. Housekeeping genes as internal standards: use and limits. Journal of Biotechnology 1999;75(2-3):291–295.
35. Robinson MD, Oshlack A. A scaling normalization method for differential expression analysis of RNA-seq data. Genome Biology 2010;11(3):R25.

36. Risso D, Ngai J, Speed TP, Dudoit S. Normalization of RNA-seq data using factor analysis of control genes or samples. Nature Biotechnology 2014;32(9):896.
37. Gagnon-Bartsch JA, Speed TP. Using control genes to correct for unwanted variation in microarray data. Biostatistics 2012;13(3):539–552.
38. Ghazanfar S, Bisogni AJ, Ormerod JT, Lin DM, Yang JY. Integrated single cell data analysis reveals cell specific networks and novel coactivation markers. BMC Systems Biology 2016;10(5):127.
39. Kharchenko PV, Silberstein L, Scadden DT. Bayesian approach to single-cell differential expression analysis. Nature Methods 2014;11(7):740.
40. Bhargava V, Head SR, Ordoukhanian P, Mercola M, Subramaniam S. Technical variations in low-input RNA-seq methodologies. Scientific reports 2014;4:3678.
41. Varadarajulu J, Schmitt A, Falkai P, Alsaif M, Turck CW, Martins-de Souza D. Differential expression of HINT1 in schizophrenia brain tissue. European Archives of Psychiatry and Clinical Neuroscience 2012;262(2):167–172.
42. Rubie C, Kempf K, Hans J, Su T, Tilton B, Georg T, et al. Housekeeping gene variability in normal and cancerous colorectal, pancreatic, esophageal, gastric and hepatic tissues. Molecular and Cellular Probes 2005;19(2):101–109.
43. Suzuki T, Higgins P, Crawford D, et al. Control selection for RNA quantitation. Biotechniques 2000;29(2):332–337.
44. Zhang L, Li WH. Mammalian housekeeping genes evolve more slowly than tissue-specific genes. Molecular Biology and Evolution 2004;21(2):236–239.
45. Zhu J, He F, Hu S, Yu J. On the nature of human housekeeping genes. Trends in Genetics 2008;24(10):481–484.
46. Sémon M, Mouchiroud D, Duret L. Relationship between gene expression and GC-content in mammals: statistical significance and biological relevance. Human Molecular Genetics 2004;14(3):421–427.
47. Arukwe A. Toxicological housekeeping genes: do they really keep the house? Environmental Science & Technology 2006;40(24):7944–7949.
48. Bacher R, Chu LF, Leng N, Gasch AP, Thomson JA, Stewart RM, et al. SCnorm: robust normalization of single-cell RNA-seq data. Nature Methods 2017;14(6):584.
49. Lun AT, Bach K, Marioni JC. Pooling across cells to normalize single-cell RNA sequencing data with many zero counts. Genome Biology 2016;17(1):75.
50. Rand WM. Objective criteria for the evaluation of clustering methods. Journal of the American Statistical Association 1971;66(336):846–850.
51. Milligan GW, Cooper MC. A study of the comparability of external criteria for hierarchical cluster analysis. Multivariate Behavioral Research 1986;21(4):441–458.
52. Fowlkes EB, Mallows CL. A method for comparing two hierarchical clusterings. Journal of the American Statistical Association 1983;78(383):553–569.
53. Amigó E, Gonzalo J, Artiles J, Verdejo F. A comparison of extrinsic clustering evaluation metrics based on formal constraints. Information Retrieval 2009;12(4):461–486.
54. Durinck S, Moreau Y, Kasprzyk A, Davis S, De Moor B, Brazma A, et al. BioMart and Bioconductor: a powerful link between biological databases and microarray data analysis. Bioinformatics 2005;21(16):3439–3440.
55. Consortium GO. Expansion of the Gene Ontology knowledgebase and resources. Nucleic Acids Research 2016;45(D1):D331–D338.
56. Croft D, Mundo AF, Haw R, Milacic M, Weiser J, Wu G, et al. The Reactome pathway knowledgebase. Nucleic acids research 2013;42(D1):D472–D477.

Response letter with graphical panels

Click here to access/download
Supplementary Material
Response_v4.docx

Click here to access/download
Supplementary Material
Supplementary Table 1.xlsx

Personal Cover Click here to access/download;Personal Cover;CoverLetter_v9.pdf

THE UNIVERSITY OF SYDNEY

School of Mathematics and Statistics

Faculty of Science,
Carslaw Building F07,
NSW 2006 AUSTRALIA
Telephone: +61 2 9351 3012
Fax: + 61 2 9351 4533
Email: pengyi.yang@sydney.edu.au

25 June 2019

Dear Editor Zhou,

Please find enclosed our revised manuscript (**GIGA-D-18-00467R1**).

We have now carried out suggested revisions in our manuscript. We hope that our point-by-point response to the reviewer enclosed with the submission addresses any remaining questions. Thank you again for considering our work for publication.

Yours faithfully,

Pengyi Yang and Jean Yang
(on behalf of all authors)
