# Supplementary material for: Evaluating stably expressed genes in single cells
Source: Gigascience. 2019 Sep 16;8(9):giz106. doi: 10.1093/gigascience/giz106 (PMC6748759; doi:10.1093/gigascience/giz106)
Supplement: giz106_Response_to_Reviewer_Comments_Original_Submission [file giz106_response_to_reviewer_comments_original_submission.pdf]

Author's Response To Reviewer Comments

Close

Reviewer Comments:

Reviewer #1:
In this manuscript, Lin and colleagues proposed a computational method to identify the stability expressed genes in single cell RNA-seq dataset. The authors downloaded two scRNA-seq dataset from the early human and mouse development, and performed their developed method, considered the mixture model Gamma-Gaussian distribution of the gene expression, to generated two SEG datasets. Then the authors did comparative analyses between the SEGs and HKGs defined previously from bulk samples, to demonstrate that SEGs are considerably more stable than HKGs. At last, the author extended the SEGs list to other eight additional datasets, and then detected some characteristics or features in SEG and comprise with those in HKGs.

In single cell transcriptome analysis, normalization and calibration are important, but difficult due to the gene expression stochasticity in single cell level. House keeping gene, rely on its stably expressed across tissues and species, always be used for normalization and calibration to reduce batch bias and background noises. SEGs proposed by the authors could be treated as a replacement of HKG for this purpose in single cell analysis. To do so, it's very important to generate a list of consistent and wildly stability expressed across different cell types, tissue, and even species. But obviously, this research serious lack this scope due to its limited samples and cell number. Moreover, for the comparative analysis between SEG and HKG on the human early development dataset, to me, the conclusion is not clarified. Because it seems that the authors selected TOP 1076 genes in SEG list, but TOP 3804 genes in HKG list previously generated in the citation (10). The huge difference of gene number maybe the most key factor in any statistical tests, so that any significant results for SEG could be overestimated.

Response: We thank this review for the insightful comments on our work. We note that, compared to the previously defined HKG lists, the human and mouse SEG lists generated from the human and mouse embryogenesis scRNA-seq datasets have been shown to be more effective for normalising and integrating multiple scRNA-seq datasets in our recently published work of scMerge (Lin et al. PNAS, 116:9775-9784, (2019) https://doi.org/10.1073/pnas.1820006116). We appreciate the criticism on the limitation of cell number in the two scRNA-seq datasets used to identify SEGs and we have now also utilised the 'mouse atlas' scRNA-seq data (SMART-Seq2) generated by the Tabula Muris Consortium (Nature 562, 367-372, 2018) for identifying SEGs in mouse.

We would like to emphasise that the purpose of this study is to evaluate the stability of genes on single-cell level as well as the proposed framework in identifying SEGs from scRNA-seq datasets. We do not suggest the current derived lists are final (albeit we have found them to be effective for normalising scRNA-seq datasets). The proposed framework (now implemented as part of the scMerge Bioconductor package (https://bioconductor.org/packages/release/bioc/html/scMerge.html)) can be applied to identify more refined SEGs from more comprehensive 'atlas' scRNA-seq datasets when they become available. This will allow increasingly high quality SEG lists to be defined/updated.

Major comments:
1) In comparative analysis between SEG and HKG, either increase the number of TOP SEG, or decrease HKG, to a comparable gene number in both sides. The 3804 HKGs, as described in citation (10), were generated by the criteria of reverse increasing order variance over tissues. In addition, it's important to do a future comparison between the TOP SEGs and the proposed 11 HKGs for calibration in citation (10).

Response: As suggested, we have decreased the HKG list defined using bulk RNA-seq to match our SEG lists and have also decreased the SEG lists to match the HKG list defined using bulk microarray. We found that the reduction of either list to match the other had relatively small effect on the clustering results as quantified by the four evaluation metrics. These new results are presented in Figure 4D and we have also updated all benchmark results in Table 2 using either full SEG lists or size matched lists in

the revised manuscript.

We have previously tested the 11 HKGs defined in citation (10) for scRNA-seq data normalisation and integration using scMerge. The boxplots on the right side (attached in the "response letter with graphical panels") compare the effects of different gene lists for Liver data normalisation and integration (see Figure 1 and Figure 2 of Lin et al. PNAS, 116:9775-9784, (2019)). The y axis represents the F1 score of Silhouette coefficients between cell type mixing and 1-batch mixing, where higher values are more desirable. The results from using 11 HKGs are lower than using the full lists of HKGs or SEGs.

2) Expending the single cell RNAseq data as much as possible, such as tumor microenvirenment atlas, organ atlas, blood atlas, and brain atlas that published in recent years.

Response: As suggested, we have now included the 'mouse atlas' scRNA-seq dataset generated by the Tabula Muris Consortium (Nature 562, 367-372, 2018) to identify SEGs and updated the SEG list for mouse in the revised manuscript as well as all associated analyses and figure presentations. Having done this, we wish to note that this study is not intended to derive final SEG lists but to provide and evaluate the proposed analytic methods for identifying SEGs. We envisage to update the SEG lists at regular intervals as more comprehensive datasets (e.g. 'human cell atlas') become available.

3) To clarify the conclusion "The stability indices also showed relatively high correlation between human and mouse (Figure 2D), suggesting gene expression stability is conserved across species". I don't think that R=0.58 could be prove that. The overlap of hSEG and mSEG makes up about 24% and 31% in human and mouse respectively. Lacking any statistical test and directly evidences to demonstrate this conclusion.

Response: We appreciate this concern. We have now performed a permutation test by randomly selecting from all quantified human genes and overlapping them with mouse SEGs and vice versa. The distribution of the permutation (b = 50,000) are shown below (attached in the "response letter with graphical panels"). The average percentage of overlaps are around 2%. These results suggest that overlaps of 25% and 30% (based on the updated mouse SEGs) in human and mouse, respectively, are statistically significant. We have revised the manuscript to clarify this point (section "Comparative analysis of SEGs identified in…"; paragraph 2).

Minor comments:
4) Key words, "single cell; scRNA-seq; Single-cell transcriptome; Bulk transcriptome", are similar concepts regarding in the scope of this manuscript.

Response: We have updated the key words, removing “Single-cell transcriptome” and adding “Gene expression variability”.

5) List all of the SEGs in SM according stable indices.

Response: As suggested, we have now listed all SEGs according to their stable indices in supplementary table 1.

6) Page 4, Figure 3D. Suggest to choose gene from 11 gens listed in table 1 at citation (10) as the canonical HKG, instead of GAPDH and ACTB.

Response: We thank the reviewer for this suggestion. The purpose of Figure 3D (now Figure 3E of the revised manuscript) is to demonstrate the bimodality of some HKGs. We, of course, do not suggest all HKGs are bimodal and the choice here is for demonstrating the additional layer of information scRNA-seq data could provide.

7) Page 4, Figure 3A. It's strange that the value of log2(gene expression) could be smaller than 1.

Response: The gene expression values x (e.g. FPKM or CPM) are log-transformed as log2(x+1). Therefore, the transformed values may be smaller than 1. We have clarified this in "scRNA-seq data processing" section on page 2 of the revised manuscript.

8) Page 5, line 50-51, where is the section 3.3?

Response: We apologise for this typo. We have corrected this in the revised manuscript.

9) Page 5, line 43-45 in right half: do the calculating the payloP score on a equivalent gene number for SEG and HKG. Moreover, as Figure 5B showed the conservation of hSEG and HKG RNA-seq didn't result in that much difference.

Response: As suggested, we have now compared the conservation score (phyloP) using similar number of genes. Specifically, we used the overlap human and mouse SEGs (n=272) and the overlap HKGs from RNA-seq and microarray data (n=277). We then compared the conservation scores of these two lists of genes calculated from human and mouse genomes. We found that SEGs identified in this study are significantly more conserved compared to HKGs defined previously. We have now included these new results in Figure 5D of the revised manuscript.

10) Present more detailed results of functional analyses in the section of "Gene stability index derived from single cells correlates with gene sequence and structural characteristics", such as the results of GO enrichment, and other pathway analyses.

Response: As suggested, we have now included more enrichment analyses using GO (biological process and molecular function) and Reactome pathway database. These results are now presented in Figure 5C of the revised manuscript.

Reviewer #2:
This paper aims to identify stably expressed genes (SEGs) from single cell RNA-seq data. Conceptually, these genes are more stable genes than house-keeping genes (HKGs) detected by microarray or bulk RNA-seq data and there are many scientific merits in this paper. But there are also concerns described below.

Response:
We thank the constructive comments from this reviewer. We have addressed each of the point in detail as below.

Major:
1. Motivation and impact: Identifying house-keeping genes has its own significance scientifically but its impact is not emphasized enough to me in this paper. It is stated that the finding can improve normalization. In microarray era, house-keeping genes were indeed useful for normalizing intensity signals. But this utility is diminished in RNA-seq and scRNA-seq. Current normalization methods of scRNA-seq data (e.g. Bacher et al. and Lin et al. cited in this paper) do not utilize house-keeping genes. The authors should spend effort to explain further the general scientific impact of identified house-keeping genes.

Response: We thank the reviewer for this suggestion. We note that our work on scMerge (Lin et al. PNAS, 116:9775-9784, (2019), https://doi.org/10.1073/pnas.1820006116) do utilises SEGs for scRNA-seq data normalisation. As suggested, we have now revised the manuscript to further highlight the utility of the SEGs identified from the proposed framework in scRNA-seq data normalisation and integration (Introduction, paragraph 4 and 5; and Discussion, paragraph 4).

2. Approach: The proposed algorithm raises concerns. (a) the selection of gamma-Gaussian model is not justified. Do real data support the model-fitting in most genes? Why the gamma tail is only on the left-end, not on the right-end? (b) Using 80 rank percentile and and minimal 60% in all criteria is an ad hoc selection. In general, there can be many ways to obtain SEGs. Is the current method better than, for example, previous methods for obtaining HKGs in bulk-RNA experiments? (c) Claiming a fixed set of house-keeping genes (a dichotomized decision) can be problematic. Providing a rank order with certain stability score is more helpful; for example, a biologist may be interested in obtaining the top 10 house-keeping genes for validation or internal control in experiment. (d) The description of w*=w\dcot minmax(\mu) is not clear.

Response: We appreciate these concerns. (a) The choice of the Gamma-Gaussian mixture model is explained in Ghazanfar et al. 2016 (https://doi.org/10.1186/s12918-016-0370-4). The reason for using a Gamma component for the left-end of the distribution is based on the non-negative values of gene expressions (log2(x+1)). To further investigate if a Gamma component will be more appropriate for the

right-end of the distribution, we have now performed additional experiments comparing (1) Gamma-Gaussian mixture (in blue) and (2) Gamma-Gamma mixture (in yellow) models across four datasets using Bayesian information criterion (BIC) to determine optimal model choice. We found that in most cases Gamma-Gaussian mixture is selected according to BIC, suggesting it provides a better fit compared to Gamma-Gamma mixture (attached in the "response letter with graphical panels"). We have now incorporated these results in Figure 1B of the revised manuscript.

(b, c) We agree that using an "ad hoc" cutoff is not ideal. Nevertheless, we note that the selection of a set of SEGs is primarily for evaluation purposes and our framework does generate stability index for each gene and therefore provides a continuum ranking of all genes. We have included the stability index as well as other stability statistics of all genes in Supplementary Table 1. Biologists can select a desired number of genes based on the stability index for validation or internal control of their experiments. (d) We have now added more explanation to clarify the regularization of proportion of zeros (w*) in the revised manuscript (page 3, paragraph 3).

3. Evaluation: (1) The evaluation in Figure 3A and Figure 4 has over-fitting. The SEGs are identified from the scRNA-seq data and evaluated in the same dataset, which generated great improvement of the new SEGs compared to the old HKGs. On the other hand, the magnitude of improvement in Table 2 is significantly lower because the evaluation is performed in independent datasets. The authors should acknowledge this artifact and avoid exaggeration in the description. (2) Since there're four datasets in both human and mouse, an ultimate solution is to obtain SEGs from random subsets of data sets (subsets of one, two or three data sets) and perform evaluation of comparing SEGs and HKGs in the remaining independent datasets. The purpose is to evaluate whether combining increasing number of datasets can generate better SEGs. And if the conclusion is true, the final claimed SEGs should be obtained from combining all four datasets, in human and mouse separately.

Response: (1) We are aware of the overfitting in testing the stability of SEGs defined from the human and mouse development datasets. As suggested, we have further emphasised this in the revised manuscript to avoid any over-interpretation (e.g. section "Genes are reproducibly ranked by their expression stability in single cells"; first paragraph). (2) We appreciate the suggestion to randomly combine datasets for defining SEGs. However, because these scRNA-seq datasets are generated from using different protocols, platforms and labs and quantified different numbers of genes at different depths in their original experiments, it is challenging to consolidate across all these datasets and normalise the unwanted factors prior to identifying SEGs. Instead, in the revised manuscript, we have incorporated the 'mouse atlas' scRNA-seq data (SMART-Seq2) generated by the Tabula Muris Consortium (Nature 562, 367-372, 2018) for identifying SEGs. Furthermore, the framework for SEG identification is now implemented as 'scSEGIndex' and form part of the scMerge Bioconductor package (https://bioconductor.org/packages/release/bioc/html/scMerge.html), allowing SEGs to be updated when more comprehensive scRNA-seq atlas become available.

4. Conceptually, SEGs detected by scRNA-seq should (very likely) be subset of HKGs detected by bulk RNA experiments but the reverse is not true. In other words, SEGs from scRNA-seq should be a subset of HKGs. But the currently analysis did not demonstrate this trend. Or do I miss anything? Again, if this is true, it'll be interesting to see combining more datasets will generate better SEGs, as a subset of HKGs.

Response: We agree with the reviewer that in the ideal world, SEGs should be a subset of HKGs. In fact, the current analysis indeed shows that more than 62% of the human SEGs (i.e. 676/1076) are also found previously as HKGs using bulk RNA experiments. Given the drastic difference in biological technologies and samples, and also different computational methodologies they are derived from, we found such an overlap is quite reassuring.

5. The authors could perform further analysis on the cross-species comparison. It is only stated that there's high concordance between human and mouse SEGs (Figure 2D, r=0.58). But from the scatter plot with correlation 0.58, it is hard to claim "high concordance". It would be helpful to see if combining more datasets generates higher concordance between human and mouse. This gives some clue whether the difference comes from intrinsic difference across species or because of the need of more datasets.

Response: As suggested, we have performed additional analyses using SEG index defined from 'mouse atlas' scRNA-seq dataset. These results are included in new Figure 2D of the manuscript. In summary, we didn’t find much difference in concordance between human and mouse gene stability index calculated from using mouse and human development datasets (r=0.58) or using mouse atlas and human

development datasets (r=0.54). The concordance of gene stability index calculated from mouse development dataset and mouse atlas is r=0.56. We also performed a permutation test comparing the percentages of SEGs overlapped between human and mouse with null distributions defined using human and mouse genes, respectively (see response 3 to reviewer #1). While we acknowledge the intrinsic difference among species, these results demonstrate that stability of genes in human and mouse are conserved. We have now revised the manuscript to discuss this point (page 3, paragraph 2).

Minor:
1. Similar to the above section (3.3)... (There is no section 3.3)

Response: We apologise for this typo. We have corrected this in the revised manuscript.

2. The magnitude in Figure 5A is not very clear. It's more helpful to show full information of p-value and statistics in supplement.

Response: As suggested, we have increased the size of Figure 5. For the correlation plots (Figure 5A), we have now also included p-value information for those that have a p-value greater than 0.001.

Close
