# Supplementary material for: Evaluating stably expressed genes in single cells
Source: Gigascience. 2019 Sep 16;8(9):giz106. doi: 10.1093/gigascience/giz106 (PMC6748759; doi:10.1093/gigascience/giz106)
Supplement: giz106_Response_to_Reviewer_Comments_Revision_1 [file giz106_response_to_reviewer_comments_revision_1.pdf]

Author's Response To Reviewer Comments

Close

Reviewer Comments:

Reviewer #1:
In this manuscript #GIGA-D-18-00467R1, authors applied a computational framework that they previous proposed to identify stably expressed genes (SEG) mainly from three scRNA-seq dataset (one from human and two from mouse). Authors did comparison analysis to prove that the SEGs defined by scRNA-seq are more stable than previously reported housekeeping(HSK) genes from bulk microarray or RNA-seq. Defining a credible SEGs is an interesting topic and important for normalization of scRNA-seq. Here are still some comments for this manuscript.

Major comments
1. It is obvious that the SEGs are different generated from different scRNA-seq datasets. As figure 3b showed, 830 and 916 mSEGs were identified from early mouse development data and union of mouse development and mouse atlas data respectively. It will be better to generate a more reliable SEGs by using several datasets. Or at least assess how different the SEGs from different dataset.

Response: We appreciate the suggestion to use several datasets for deriving SEGs. However, since different scRNA-seq datasets are often generated from using different protocols, platforms and quantified different numbers of genes at different depths in their original experiments, it is challenging to consolidate across multiple datasets and normalise the unwanted factors prior to identifying SEGs. More importantly, datasets used for SEG identification should contain normal cell types and profile heterogenous tissues and cell types because data only contain homogeneous cell types cannot provide the foundation for identifying genes stably expressed in different tissues/cell types, and data contain abnormal cell types such as cancers may derive genes that are abnormally stable in cancers. Therefore, it seems to us that it is preferable to limit the number of datasets and instead choose those that comprehensively profile normal tissues/cell types and developmental stages. We have noted this in the fourth paragraph in the discussion section of the revised manuscript.

To demonstrate the above point, we have now also used our framework to identify "hSEGs" from Multicellular metastatic melanoma dataset (see Table 1 of the manuscript). While this dataset contains a relatively large number of cells, it has resulted a much different stability index compared to those derived from human development dataset as shown in the scatter plot (attached in the "response letter with graphical panels"). When tested for stability using the evaluation dataset (Adult and fetal brain), these "hSEGs" derived from melanoma tissues are not better than using all genes and much less effective compare to those derived from human development dataset as can be seen in table below (attached in the "response letter with graphical panels").

2. In the section "Comparative analysis of SEGs identified in single cells and HKGs defined from bulk transcriptome", that the author did comparison between SEGs and HKGs only based on SEGs generating dataset is not enough to support the conclusion. Other evaluate datasets mentioned in table 1 should also be used to do the comparison.

Response: As we have demonstrated above, datasets that profile diverse tissue types and developmental stages are best suited for deriving SEGs. We hope this reviewer agrees with us that the comparative analysis of SEGs and HKGs should be performed using SEGs derived from the three SEG identification datasets. Drawing conclusions from using more restricted profiling datasets (e.g. peripheral blood) and/or datasets that profiling abnormal tissues (e.g. melanoma) may lead to incorrect conclusions.

3.It will be better to explain the selection criteria of SEG more clear, like why set the cutoff of 80 and 60.

Response: The selection of a set of SEGs is primarily for evaluation purposes and our framework

provides a continuum ranking of all genes by generating a stability index. As suggested, we have now noted this in the revised manuscript (Discussion section, paragraph 4).

Minor comments
1.There should be randomly sampling for several times in the analysis of figure 2C, so the range of the r should be mentioned in manuscript.

Response: As suggested, we have included the standard deviation of correlation from multiple sampling in figure 2C and updated the figure legend.

2.There are some incorrect spellings. Like figure legend of figure 3B "altas" should be "atlas"; (E), at the end figure legend of figure 3D should be deleted.

Response: We thank the reviewer for identifying these typos. We have corrected them.

3.It needs to label the test approach used in figure 5D.

Response: As suggested, we have stated the test approach in the legend of Figure 5D in the revised manuscript.

4. There is some problem with the format of reference 57.

Response: We have rectified this in the revised manuscript.

Close
