# Supplementary material for: Evaluating stably expressed genes in single cells
Source: Gigascience. 2019 Sep 16;8(9):giz106. doi: 10.1093/gigascience/giz106 (PMC6748759; doi:10.1093/gigascience/giz106)
Supplement: giz106_Response_to_Reviewer_Comments_Revision_2 [file giz106_response_to_reviewer_comments_revision_2.pdf]

Author's Response To Reviewer Comments

Close

Dear Dr Zhou,

We have revised the manuscript to (1) replace scSEGIndex links; (2) include ORCID identifiers; (3) include GigaDB link; and (4) include a "Availability of supporting source code and requirements" section. Please let us know if there is anything else we could do. Thank you for your assistance.

Kind regards,
Pengyi

Close
