# Supplementary material for: Evaluating stably expressed genes in single cells
Source: Gigascience. 2019 Sep 16;8(9):giz106. doi: 10.1093/gigascience/giz106 (PMC6748759; doi:10.1093/gigascience/giz106)
Supplement: giz106_Reviewer_1_Report_Original_Submission — Yong Hou -- 12/10/2018 Reviewed [file giz106_reviewer_1_report_original_submission.pdf]

Reviewer Report

Title: Evaluating stably expressed genes in single cells

Version: Original Submission **Date:** 12/10/2018

Reviewer name: Yong Hou

Reviewer Comments to Author:

In this manuscript, Lin and colleagues proposed a computational method to identify the stability expressed genes in single cell RNA-seq dataset. The authors downloaded two scRNA-seq dataset from the early human and mouse development, and performed their developed method, considered the mixture model Gamma-Gaussian distribution of the gene expression, to generated two SEG datasets. Then the authors did comparative analyses between the SEGs and HKGs defined previously from bulk samples, to demonstrate that SEGs are considerably more stable than HKGs. At last, the author extended the SEGs list to other eight additional datasets, and then detected some characteristics or features in SEG and comprise with those in HKGs.

In single cell transcriptome analysis, normalization and calibration are important, but difficult due to the gene expression stochasticity in single cell level. House keeping gene, rely on its stably expressed across tissues and species, always be used for normalization and calibration to reduce batch bias and background noises. SEGs proposed by the authors could be treated as a replacement of HKG for this purpose in single cell analysis. To do so, it's very important to generate a list of consistent and wildly stability expressed across different cell types, tissue, and even species. But obviously, this research serious lack this scope due to its limited samples and cell number. Moreover, for the comparative analysis between SEG and HKG on the human early development dataset, to me, the conclusion is not clarified. Because it seems that the authors selected TOP 1076 genes in SEG list, but TOP 3804 genes in HKG list previously generated in the citation (10). The huge difference of gene number maybe the most key factor in any statistical tests, so that any significant results for SEG could be overestimated.

Major comments:

1) In comparative analysis between SEG and HKG, either increase the number of TOP SEG, or decrease HKG, to a comparable gene number in both sides. The 3804 HKGs, as described in citation (10), were generated by the criteria of reverse increasing order variance over tissues. In addition, it's important to do a future comparison between the TOP SEGs and the proposed 11 HKGs for calibration in citation (10).

2) Expending the single cell RNAseq data as much as possible, such as tumor microenvirenment atlas, organ atlas, blood atlas, and brain atlas that published in recent years.

3) To clarify the conclusion "The stability indices also showed relatively high correlation between human and mouse (Figure 2D), suggesting gene expression stability is conserved across species". I don't think that R=0.58 could be prove that. The overlap of hSEG and mSEG makes up about 24% and 31% in human and mouse respectively. Lacking any statistical test and directly evidences to demonstrate this conclusion.

Minor comments:

4) Key words, "single cell; scRNA-seq; Single-cell transcriptome; Bulk transcriptome", are similar concepts regarding in the scope of this manuscript.
5) List all of the SEGs in SM according stable indices.
6) Page 4, Figure 3D. Suggest to choose gene from 11 gens listed in table 1 at citation (10) as the canonical HKG, instead of GAPDH and ACTB.
7) Page 4, Figure 3A. It's strange that the value of log2(gene expression) could be smaller than 1.
8) Page 5, line 50-51, where is the section 3.3?
9) Page 5, line 43-45 in right half: do the calculating the payloP score on a equivalent gene number for SEG and HKG. Moreover, as Figure 5B showed the conservation of hSEG and HKG RNA-seq didn't result in that much difference.
10) Present more detailed results of functional analyses in the section of "Gene stability index derived from single cells correlates with gene sequence and structural characteristics", such as the results of GO enrichment, and other pathway analyses.

Methods

Are the methods appropriate to the aims of the study, are they well described, and are necessary controls included? Choose an item.

Conclusions

Are the conclusions adequately supported by the data shown? Choose an item.

Reporting Standards

Does the manuscript adhere to the journal's guidelines on minimum standards of reporting? Choose an item.

Choose an item.

Statistics

Are you able to assess all statistics in the manuscript, including the appropriateness of statistical tests used? Choose an item.

Quality of Written English

Please indicate the quality of language in the manuscript: Choose an item.

Declaration of Competing Interests

Please complete a declaration of competing interests, considering the following questions:

- Have you in the past five years received reimbursements, fees, funding, or salary from an organisation that may in any way gain or lose financially from the publication of this manuscript, either now or in the future?
- Do you hold any stocks or shares in an organisation that may in any way gain or lose financially from the publication of this manuscript, either now or in the future?
- Do you hold or are you currently applying for any patents relating to the content of the manuscript?
- Have you received reimbursements, fees, funding, or salary from an organization that holds or has applied for patents relating to the content of the manuscript?
- Do you have any other financial competing interests?
- Do you have any non-financial competing interests in relation to this paper?

If you can answer no to all of the above, write 'I declare that I have no competing interests' below. If your reply is yes to any, please give details below.

I declare that I have no competing interests

I agree to the open peer review policy of the journal. I understand that my name will be included on my report to the authors and, if the manuscript is accepted for publication, my named report including any attachments I upload will be posted on the website along with the authors' responses. I agree for my report to be made available under an Open Access Creative Commons CC-BY license (http://creativecommons.org/licenses/by/4.0/). I understand that any comments which I do not wish to be included in my named report can be included as confidential comments to the editors, which will not be published.

Choose an item.

To further support our reviewers, we have joined with Publons, where you can gain additional credit to further highlight your hard work (see: https://publons.com/journal/530/gigascience). On publication of this paper, your review will be automatically added to Publons, you can then choose whether or not to claim your Publons credit. I understand this statement.

Yes Choose an item.
