# Supplementary material for: Evaluating stably expressed genes in single cells
Source: Gigascience. 2019 Sep 16;8(9):giz106. doi: 10.1093/gigascience/giz106 (PMC6748759; doi:10.1093/gigascience/giz106)
Supplement: giz106_Reviewer_1_Report_Revision_1 — Yong Hou -- 6/10/2019 Reviewed [file giz106_reviewer_1_report_revision_1.pdf]

Reviewer Report

Title: Evaluating stably expressed genes in single cells

Version: Revision 1 **Date:** 6/10/2019

Reviewer name: Yong Hou

Reviewer Comments to Author:

In this manuscript #GIGA-D-18-00467R1, authors applied a computational framework that they previous proposed to identify stably expressed genes (SEG) mainly from three scRNA-seq dataset (one from human and two from mouse). Authors did comparison analysis to prove that the SEGs defined by scRNA-seq are more stable than previously reported housekeeping(HSK) genes from bulk microarray or RNA-seq. Defining a credible SEGs is an interesting topic and important for normalization of scRNA-seq. Here are still some comments for this manuscript.

Major comments

1.It is obvious that the SEGs are different generated from different scRNA-seq datasets. As figure 3b showed, 830 and 916 mSEGs were identified from early mouse development data and union of mouse development and mouse atlas data respectively. It will be better to generate a more reliable SEGs by using several datasets. Or at least assess how different the SEGs from different dataset.

2.In the section "Comparative analysis of SEGs identified in single cells and HKGs defined from bulk transcriptome", that the author did comparison between SEGs and HKGs only based on SEGs generating dataset is not enough to support the conclusion. Other evaluate datasets mentioned in table 1 should also be used to do the comparison.

3.It will be better to explain the selection criteria of SEG more clear, like why set the cutoff of 80 and 60.

Minor comments

1.There should be randomly sampling for several times in the analysis of figure 2C, so the range of the r should be mentioned in manuscript.

2.There are some incorrect spellings. Like figure legend of figure 3B "altas" should be "atlas"; (E), at the end figure legend of figure 3D should be deleted.

3.It needs to label the test approach used in figure 5D.

4. There is some problem with the format of reference 57.

Methods

Are the methods appropriate to the aims of the study, are they well described, and are necessary controls included? Choose an item.

Conclusions

Are the conclusions adequately supported by the data shown? Choose an item.

Reporting Standards

Does the manuscript adhere to the journal's guidelines on minimum standards of reporting? Choose an item.

Choose an item.

Statistics

Are you able to assess all statistics in the manuscript, including the appropriateness of statistical tests used? Choose an item.

Quality of Written English

Please indicate the quality of language in the manuscript: Choose an item.

Declaration of Competing Interests

Please complete a declaration of competing interests, considering the following questions:

- Have you in the past five years received reimbursements, fees, funding, or salary from an organisation that may in any way gain or lose financially from the publication of this manuscript, either now or in the future?
- Do you hold any stocks or shares in an organisation that may in any way gain or lose financially from the publication of this manuscript, either now or in the future?
- Do you hold or are you currently applying for any patents relating to the content of the manuscript?
- Have you received reimbursements, fees, funding, or salary from an organization that holds or has applied for patents relating to the content of the manuscript?
- Do you have any other financial competing interests?
- Do you have any non-financial competing interests in relation to this paper?

If you can answer no to all of the above, write 'I declare that I have no competing interests' below. If your reply is yes to any, please give details below.

no

I agree to the open peer review policy of the journal. I understand that my name will be included on my report to the authors and, if the manuscript is accepted for publication, my named report including any attachments I upload will be posted on the website along with the authors' responses. I agree for my report to be made available under an Open Access Creative Commons CC-BY license (http://creativecommons.org/licenses/by/4.0/). I understand that any comments which I do not wish to be included in my named report can be included as confidential comments to the editors, which will not be published.

Choose an item.

To further support our reviewers, we have joined with Publons, where you can gain additional credit to further highlight your hard work (see: https://publons.com/journal/530/gigascience). On publication of this paper, your review will be automatically added to Publons, you can then choose whether or not to claim your Publons credit. I understand this statement.

Yes Choose an item.
