# Supplementary material for: Evaluating stably expressed genes in single cells
Source: Gigascience. 2019 Sep 16;8(9):giz106. doi: 10.1093/gigascience/giz106 (PMC6748759; doi:10.1093/gigascience/giz106)
Supplement: giz106_Reviewer_2_Report_Original_Submission — George Tseng -- 4/6/2019 Reviewed [file giz106_reviewer_2_report_original_submission.pdf]

**Reviewer Report**

**Title: Evaluating stably expressed genes in single cells**

**Version: Original Submission** **Date:** 4/6/2019

**Reviewer name: George Tseng**

**Reviewer Comments to Author:**

This paper aims to identify stably expressed genes (SEGs) from single cell RNA-seq data. Conceptually, these genes are more stable genes than house-keeping genes (HKGs) detected by microarray or bulk RNA-seq data and there are many scientific merits in this paper. But there are also concerns described below.

Major:

1. Motivation and impact: Identifying house-keeping genes has its own significance scientifically but its impact is not emphasized enough to me in this paper. It is stated that the finding can improve normalization. In microarray era, house-keeping genes were indeed useful for normalizing intensity signals. But this utility is diminished in RNA-seq and scRNA-seq. Current normalization methods of scRNA-seq data (e.g. Bacher et al. and Lin et al. cited in this paper) do not utilize house-keeping genes. The authors should spend effort to explain further the general scientific impact of identified house-keeping genes.
2. Approach: The proposed algorithm raises concerns. (a) the selection of gamma-Gaussian model is not justified. Do real data support the model-fitting in most genes? Why the gamma tail is only on the left-end, not on the right-end? (b) Using 80 rank percentile and and minimal 60% in all criteria is an ad hoc selection. In general, there can be many ways to obtain SEGs. Is the current method better than, for example, previous methods for obtaining HKGs in bulk-RNA experiments? (c) Claiming a fixed set of house-keeping genes (a dichotomized decision) can be problematic. Providing a rank order with certain stability score is more helpful; for example, a biologist may be interested in obtaining the top 10 house-keeping genes for validation or internal control in experiment. (d) The description of w*=w\dcot minmax(\mu) is not clear.
3. Evaluation: (1) The evaluation in Figure 3A and Figure 4 has over-fitting. The SEGs are identified from the scRNA-seq data and evaluated in the same dataset, which generated great improvement of the new SEGs compared to the old HKGs. On the other hand, the magnitude of improvement in Table 2 is significantly lower because the evaluation is performed in independent datasets. The authors should acknowledge this artifact and avoid exaggeration in the description. (2) Since there're four datasets in both human and mouse, an ultimate solution is to obtain SEGs from random subsets of data sets (subsets of one, two or three data sets) and perform evaluation of comparing SEGs and HKGs in the remaining independent datasets. The purpose is to evaluate whether combining increasing number of datasets can generate better SEGs. And if the conclusion is true, the final claimed SEGs should be obtained from combining all four datasets, in human and mouse separately.
4. Conceptually, SEGs detected by scRNA-seq should (very likely) be subset of HKGs detected by bulk RNA experiments but the reverse is not true. In other words, SEGs from scRNA-seq should be a subset of

HKGs. But the currently analysis did not demonstrate this trend. Or do I miss anything? Again, if this is true, it'll be interesting to see combining more datasets will generate better SEGs, as a subset of HKGs.
5. The authors could perform further analysis on the cross-species comparison. It is only stated that there's high concordance between human and mouse SEGs (Figure 2D, r=0.58). But from the scatter plot with correlation 0.58, it is hard to claim "high concordance". It would be helpful to see if combining more datasets generates higher concordance between human and mouse. This gives some clue whether the difference comes from intrinsic difference across species or because of the need of more datasets.
Minor:
1. Similar to the above section (3.3)... (There is no section 3.3)
2. The magnitude in Figure 5A is not very clear. It's more helpful to show full information of p-value and statistics in supplement.

**Methods**

Are the methods appropriate to the aims of the study, are they well described, and are necessary controls included? Choose an item.

**Conclusions**

Are the conclusions adequately supported by the data shown? Choose an item.

**Reporting Standards**

Does the manuscript adhere to the journal's guidelines on minimum standards of reporting? Choose an item.

Choose an item.

**Statistics**

Are you able to assess all statistics in the manuscript, including the appropriateness of statistical tests used? Choose an item.

**Quality of Written English**

Please indicate the quality of language in the manuscript: Choose an item.

**Declaration of Competing Interests**

Please complete a declaration of competing interests, considering the following questions:

- Have you in the past five years received reimbursements, fees, funding, or salary from an organisation that may in any way gain or lose financially from the publication of this manuscript, either now or in the future?

- Do you hold any stocks or shares in an organisation that may in any way gain or lose financially from the publication of this manuscript, either now or in the future?
- Do you hold or are you currently applying for any patents relating to the content of the manuscript?
- Have you received reimbursements, fees, funding, or salary from an organization that holds or has applied for patents relating to the content of the manuscript?
- Do you have any other financial competing interests?
- Do you have any non-financial competing interests in relation to this paper?

If you can answer no to all of the above, write 'I declare that I have no competing interests' below. If your reply is yes to any, please give details below.

I declare that I have no competing interests

I agree to the open peer review policy of the journal. I understand that my name will be included on my report to the authors and, if the manuscript is accepted for publication, my named report including any attachments I upload will be posted on the website along with the authors' responses. I agree for my report to be made available under an Open Access Creative Commons CC-BY license (http://creativecommons.org/licenses/by/4.0/). I understand that any comments which I do not wish to be included in my named report can be included as confidential comments to the editors, which will not be published.

Choose an item.

To further support our reviewers, we have joined with Publons, where you can gain additional credit to further highlight your hard work (see: https://publons.com/journal/530/gigascience). On publication of this paper, your review will be automatically added to Publons, you can then choose whether or not to claim your Publons credit. I understand this statement.

Yes Choose an item.
